# Manufacturing of Placenta- and Umbilical Cord-Derived Mesenchymal Stromal Cells: A Systematic Scoping Review of Process Parameters, Quality Attributes, and Evidence-to-Control Relationships

**DOI:** 10.3390/life16101740

**Published:** 2026-10-16

**Authors:** Bolat Sadykov, Abay Baigenzhin, Anastassiya Ganina, Madina Karimova, Aizhan Akhayeva, Anuar Zhunussov, Batyrbek Assembekov, Ildar Fakhradiyev

**Affiliations:** 1 Department of Medicine, S. D. Asfendiyarov Kazakh National Medical University, Almaty 050012, Kazakhstanassembekov.b@kaznmu.kz (B.A.); 2 National Scientific Medical Center, Astana 010009, Kazakhstan; 3 College of Medicine, Korea University, Seoul 02841, Republic of Korea

**Keywords:** mesenchymal stromal cells, umbilical cord, placenta, cell manufacturing, quality by design, critical quality attributes, cryopreservation, potency

## Abstract

The manufacturing of placenta- and umbilical cord-derived mesenchymal stromal cells (MSCs) varies across anatomical starting material, isolation, culture supplementation, expansion, scale-up, cryopreservation, and release testing. This variability limits comparability between products and complicates process transfer. The study aimed to map full-text evidence on manufacturing variables, process outcomes, candidate critical material attributes (CMAs), candidate critical process parameters (CPPs), critical quality attributes (CQAs), potency assays, and release strategies for human placenta- and umbilical cord-derived MSCs. A systematic scoping review was conducted using a Population–Concept–Context framework and reported according to PRISMA-ScR, with search reporting guided by PRISMA-S. Searches without publication-date restrictions were completed on 15 August 2026. Two reviewers independently screened records and full texts, and charted data were independently verified. Findings were mapped by manufacturing contrast and outcome domain; quantitative pooling was considered only when biological, process, and methodological comparability supported an interpretable common effect estimate. The primary Scopus/Web of Science search identified 18,860 records; 13,122 unique records were screened and 439 full-text reports were assessed. After 208 exclusions, 231 manufacturing reports remained; the PubMed/MEDLINE search and backward/forward citation searching contributed two and five additional unique eligible reports, respectively, after cross-source deduplication, yielding 238 included reports. The study-family sensitivity set contained 236 distinct study families after linked-report assessment. Tissue-source coding identified 183 umbilical cord/Wharton’s jelly reports, 46 placental reports, and nine multi-source perinatal reports. The most frequent domains were expansion/passaging/senescence (94/238; 39.5%), media/supplements (69; 29.0%), isolation/tissue processing (67; 28.2%), and GMP/quality/release (48; 20.2%). Direct-comparison sets included 2D versus 3D/bioreactor expansion (23 reports), explant versus enzymatic isolation (22), fresh/native versus cryopreserved material (19), HPL versus FBS (12), and hypoxia versus normoxia (10). Differences in intervention definition, tissue compartment, passage/timepoint, endpoint, and unit did not support a coherent common pooled effect. The mapped evidence supports an end-to-end development model and does not identify a universal manufacturing recipe. Anatomical starting material, isolation, culture conditions, cumulative population doublings, scale-up, cryopreservation, and potency-linked release should be treated as interdependent elements of product comparability. Formal designation of CMAs and CPPs remains product- and platform-specific and requires evidence that links process variation to relevant CQAs.

## 1. Introduction

Placenta and umbilical cord tissues are abundant perinatal sources of mesenchymal stromal cells and can be obtained without an additional invasive procedure for the donor. Their availability has supported cell banking, process development, and allogeneic manufacturing. However, labels such as “placenta-derived MSCs” and “umbilical cord-derived MSCs” do not define a single biological starting material. Amnion, chorion, chorionic villi, decidua, cord lining, Wharton’s jelly, perivascular regions, cord matrix, and the cord–placenta junction differ in cellular composition, maternal/fetal contribution, primary yield, and expansion behavior [1,2,3].

The ISCT minimal criteria define identity-related features of MSCs but do not establish manufacturing comparability [4,5,6]. Process-oriented assessment links starting-material attributes and process parameters to product quality. In Quality-by-Design terminology, a material or process parameter should be considered critical only when variation in that parameter has a demonstrated relationship with a relevant CQA and, therefore, requires control within a defined process [5,6]. For perinatal MSCs, candidate material attributes include anatomical compartment, donor/tissue characteristics, transport interval, and properties of biological supplements; candidate process parameters include dissection, enzyme exposure, seeding density, medium, oxygen environment, cumulative population doublings, bioreactor conditions, and freezing/thawing. CQAs extend beyond identity and viability to purity, sterility, genetic stability, post-thaw recovery, and mechanism-related potency.

Existing reviews have generally addressed MSC manufacturing across mixed tissue sources or focused on discrete stages such as two-dimensional expansion, Quality-by-Design classification, or bioreactor scale-up [5,7]. That literature is informative but does not resolve the end-to-end manufacturing problem specific to placenta and umbilical cord, where the anatomical source itself may be a major determinant of downstream process behavior. Regulatory guidance for cell-based products likewise emphasizes process understanding, control of variability, and product-specific potency instead of reliance on a single generic phenotypic definition [6,8,9].

The primary literature spans isolation strategies [10,11,12,13,14,15,16,17], anatomical compartments and source comparisons [1,2,3,18,19], culture media and xeno-free systems [10,20,21,22,23,24,25,26,27,28], scale-up platforms [29,30,31,32,33,34,35], cryopreservation and storage controls [26,36,37,38,39,40,41,42,43], and GMP/release controls [27,31,32,33,39,44,45,46,47,48]. The breadth of these questions favors systematic evidence mapping and structured comparative synthesis, not estimation of one overall intervention effect.

The objective of this systematic scoping review was to map manufacturing evidence for human placenta- and umbilical cord-derived MSCs and to map reported relationships between process variables and measurable process outcomes, CQAs, potency, release criteria, and post-thaw performance. A secondary objective was to translate the mapped evidence into an evidence-to-control model that distinguishes recurrent candidate CMAs/CPPs from parameters whose criticality remains product- or platform-specific.

## 2. Materials and Methods

### 2.1. Review Design and Reporting Framework

The review was conducted as a systematic scoping review because the evidence base spans heterogeneous manufacturing questions, process stages, and outcome types rather than a single common intervention effect. Reporting follows PRISMA-ScR [49], with search reporting guided by PRISMA-S [50] and PRISMA 2020 terminology used for record management and the flow diagram [51]. The review was not registered. Eligibility criteria and review procedures were defined before formal screening. Detailed review procedures and operational definitions are provided in Supplementary Tables S1A and S1B.

### 2.2. PCC Question

Primary question (Table 1): Which manufacturing variables and control strategies have been reported for human placenta- and umbilical cord-derived MSC production, and how are these variables associated with measurable process outcomes, CQAs, potency, release criteria, and post-thaw performance?

### 2.3. Eligibility Criteria

Reports were eligible when they met all of the following criteria:

Primary research using human placenta- or umbilical cord-derived MSCs with a defined tissue source or anatomical compartment.

A manufacturing variable, process-development parameter, stability intervention, GMP workflow, or release/CQA procedure was a principal study variable.

At least one extractable process outcome, CQA, potency measure, stability outcome, or release-quality outcome was reported.

Comparative or non-comparative process-development studies were eligible when manufacturing conditions and outcomes were sufficiently described to support process interpretation.

Mixed-source studies were eligible when placenta/umbilical cord results were separately extractable or when source comparison itself was the manufacturing question.

Protocol-oriented journal reports were eligible only when they contained original, extractable manufacturing results.

Reviews, commentaries, methods-only reports without original results, animal-only studies, extracellular-vesicle/secretome studies in which MSC manufacturing was not the study question, genetic engineering or disease-model studies without a manufacturing endpoint, differentiation/mechanistic studies without an eligible process outcome, clinical-treatment studies without manufacturing/comparability/release data, and duplicate publications were excluded. Biological or mechanistic studies that provided general context but did not evaluate an eligible manufacturing variable were not part of the formal evidence corpus and were used only as background literature where relevant.

No publication-date or language restriction was applied in the database queries. Potentially eligible non-English reports were translated for full-text assessment, and eligibility and extracted data were verified against the original-language article. Abstract-only records were excluded when they did not provide sufficient process detail to verify manufacturing conditions and extract prespecified outcomes. Gray-literature and regulatory/public manufacturing sources were used as supplementary discovery and contextual sources; inclusion in the formal corpus required a retrievable primary report with sufficient manufacturing detail for source verification and extraction.

### 2.4. Information Sources and Search Strategy

Database and supplementary searches were completed on 15 August 2026. Search concepts covered (1) mesenchymal stromal/stem cells; (2) placenta, umbilical cord, Wharton’s jelly, and identifiable perinatal compartments; and (3) manufacturing variables including isolation, explant/digestion, culture medium, platelet lysate, serum-/xeno-free conditions, passage, oxygen tension, scale-up, microcarriers/bioreactors, cryopreservation, GMP, potency, quality control, and release testing. Exact strategies and restrictions are provided in Supplementary Table S2. Scopus and Web of Science formed the primary record-level search. PubMed and backward/forward citation searching were supplementary routes and were deduplicated against the primary corpus. Because complete stage-level counts for the supplementary routes were not archived, only their verified unique eligible contributions are reported (two PubMed-only reports and five citation-search-only reports); missing counts were not reconstructed.

### 2.5. Record Management and Deduplication

Search exports were managed in Microsoft Excel for Microsoft 365, with reproducible DOI/title normalization and consistency checks implemented in Python 3.x. Deduplication was performed first by normalized DOI and, when a reliable DOI match was unavailable, by normalized title plus publication year with bibliographic concordance. A total of 5738 duplicate records were removed from the primary Scopus/Web of Science exports. Potential duplicates identified by normalized or fuzzy title matching were manually verified against authorship, year, journal, DOI, tissue source, and study characteristics before removal. Translated versions of the same report were treated as duplicates. Distinct companion publications providing materially different process information were retained and evaluated for study-family overlap. A version-controlled screening workbook preserved reviewer decisions and reconciliation outcomes.

### 2.6. Selection and Full-Text Assessment

Before formal screening, the eligibility criteria were piloted independently by two reviewers on a calibration sample of records. Screening commenced only after ambiguities were resolved and prespecified acceptable agreement had been achieved; Cohen’s kappa calculated during calibration indicated strong inter-reviewer agreement. Two reviewers then independently screened titles and abstracts and subsequently assessed potentially eligible full-text reports against the predefined criteria. Records considered potentially eligible by either reviewer proceeded to full-text assessment. Disagreements were resolved by consensus, with adjudication by a third reviewer when required. All 439 reports sought for retrieval were obtained and assessed in full text; no report was excluded because the complete article was unavailable. All 238 included reports were verified against the complete article, and supplementary files were consulted when relevant. The complete list of included reports is provided in Supplementary Table S3. A summary of exclusion reasons and the report-level decisions are presented in Supplementary Table S4A,B, respectively. No author contact was required to determine eligibility or verify the numerical results used in the primary synthesis.

### 2.7. Data Charting and Data Items

A piloted standardized charting form recorded citation/identifier, publication year, anatomical tissue source, study design, manufacturing stage, manufacturing-domain codes, direct-comparison flags, study-family linkage, comparator status, GMP context, and principal process or quality outcome for every included report. For reports contributing detailed process or numerical results, extraction additionally captured donor/tissue/batch numbers; dissection and isolation conditions; enzyme identity, concentration, and exposure time; culture medium and supplements; seeding density; passage and cumulative population doublings; 2D/3D platform and bioreactor details; expansion duration; yield, fold expansion, harvest efficiency, and viability; cryopreservation and post-thaw testing; identity/purity; sterility, mycoplasma, endotoxin, and genetic stability; potency assay; release thresholds; comparator; statistics; and evidence of external validation. Data were charted by one reviewer and independently verified against the full-text source and supplementary material by a second reviewer. Before full extraction, a random subset was independently double-extracted to calibrate the charting scheme; discrepancies were resolved by consensus and the form was refined before completion of the full corpus. Numerical values were not imputed, and unreported means or standard deviations were not reconstructed.

### 2.8. Linked Reports and Analytical Unit

Companion publications were not assumed to represent independent experiments. Potentially linked reports were assessed independently by two reviewers using author group, tissue source, manufacturing platform, donor/batch counts, study period, recruitment structure, passage series, and explicit cross-reference. Reports were treated as overlapping when predefined concordance criteria supported reuse of the same experimental units or cohort. Disagreements were resolved by consensus, with third-reviewer adjudication when necessary. Two overlapping publication families were collapsed to single study families for a study-family sensitivity check. A Russian original/English translation pair was treated as a duplicate. Other potentially related reports were retained separately when shared experimental units could not be established. The study-family sensitivity set contained 236 distinct families. For each manufacturing domain or direct-comparison category, a family was counted once when at least one linked report met the corresponding coding criterion. Compared with the report-level analysis, counts decreased from 94 to 93 for expansion/passaging/senescence, from 69 to 68 for media/supplements, and from 48 to 46 for GMP/quality/release. Counts for the other six domains and the domain ranking were unchanged. Counts for the five direct-comparison categories were also unchanged. Linked-report assessments and analytical decisions are detailed in Supplementary Table S5A; report-level and study-family counts are presented in Supplementary Table S5B,C.

### 2.9. Evidence Characterization and Design-Robustness Assessment

Formal source-level critical appraisal was not undertaken. For direct comparative and detailed quantitative reports, two reviewers independently characterized design robustness using biological replication, comparator definition, same-donor/matched design, outcome completeness, statistical reporting, process reproducibility, CQA/potency assessment, post-thaw assessment, and independent validation. Disagreements were resolved by consensus. These characteristics informed narrative weighting rather than eligibility and were not combined into a composite quality or certainty score. Operational definitions are provided in Supplementary Table S1B and comparison-level interpretation in Supplementary Table S6. For the 11 studies included in the source-mapped quantitative audit (Supplementary Table S7) and three additional comparative examples, Supplementary Table S8 provides source-level comparator, matching, and biological-unit descriptors. Technical repeats were distinguished from independent donors/batches, and uncontrolled process-development observations were interpreted as evidence of feasibility within the reported workflow rather than relative superiority.

### 2.10. Synthesis and Assessment of Quantitative Pooling

Reports could contribute to more than one manufacturing domain; domain frequencies are therefore non-mutually exclusive and should not be summed as independent observations. Direct comparative evidence was organized around five recurrent contrasts: HPL versus FBS, explant versus enzymatic isolation, fresh/native versus cryopreserved material, 2D versus 3D/bioreactor expansion, and hypoxia versus normoxia. For each contrast, biological, process, and methodological comparability was assessed with respect to tissue source, intervention definition, process stage, endpoint, measurement unit, passage/timepoint, and independence of experimental datasets. Owing to heterogeneity, comparative findings were descriptively mapped by manufacturing contrast and outcome domain. Relevant SWiM reporting elements were used to improve transparency where statistical pooling was inappropriate [52]. The comparison-level mapping and heterogeneity assessment are provided in Supplementary Table S6.

## 3. Results

### 3.1. Selection of Sources of Evidence

**Figure 1:**
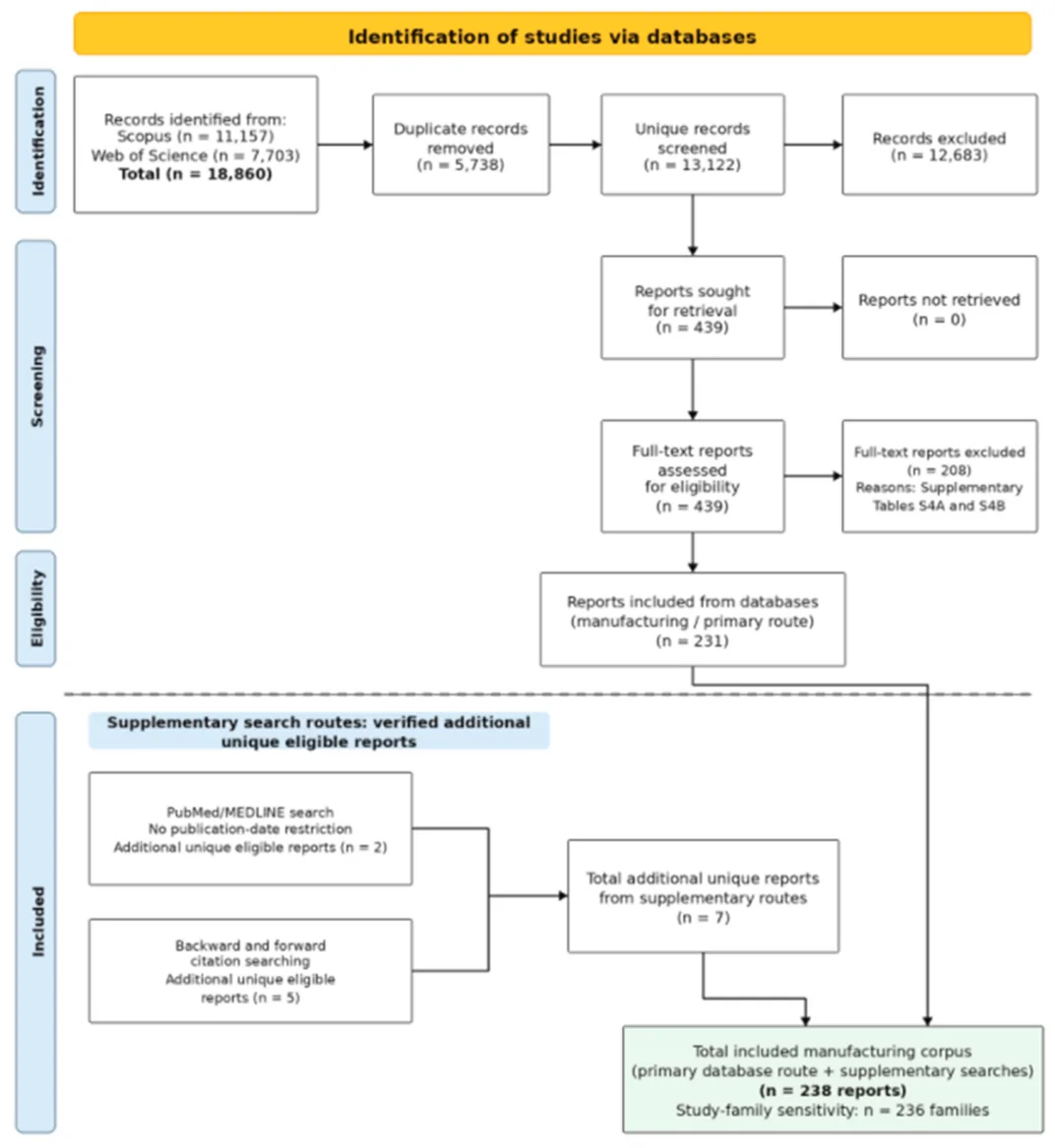
PRISMA-ScR flow for the primary Scopus/Web of Science search and the verified unique eligible contributions from supplementary PubMed and citation searching.

The Scopus and Web of Science searches identified 18,860 records: 11,157 in Scopus and 7703 in Web of Science Core Collection. After removal of 5738 duplicates, 13,122 unique records were screened and 12,683 were excluded at title/abstract level. All 439 reports sought for retrieval were obtained and assessed in full text; 208 were excluded with report-level reasons, leaving 231 manufacturing reports from the primary database route. The supplementary PubMed search and backward/forward citation searching contributed two and five additional unique eligible reports, respectively, after cross-source deduplication, yielding 238 included reports. Among the 208 full-text exclusions, 69 were biological or mechanistic reports without an eligible manufacturing-process variable or manufacturing-linked CQA outcome. The study-family sensitivity set contained 236 distinct families. The manufacturing-domain ranking and counts for the five direct-comparison categories were unchanged, although three manufacturing-domain counts decreased (Supplementary Table S5B,C). The selection process is shown in Figure 1.

### 3.2. Characteristics of the Manufacturing Corpus

The 238 included manufacturing reports were published from 2004 to 2026: 21 (8.8%) in 2004–2010, 75 (31.5%) in 2011–2015, 74 (31.1%) in 2016–2020, and 68 (28.6%) in 2021–2026. Full-text tissue-source coding identified 183 reports (76.9%) focused on umbilical cord/Wharton’s jelly, 46 (19.3%) on placenta/amnion/chorion/decidua, and nine (3.8%) on multiple perinatal sources. No included report remained in an “unspecified source” category after full-text verification. These characteristics are summarized in Table 2.

### 3.3. Manufacturing Evidence Map

**Figure 2:**
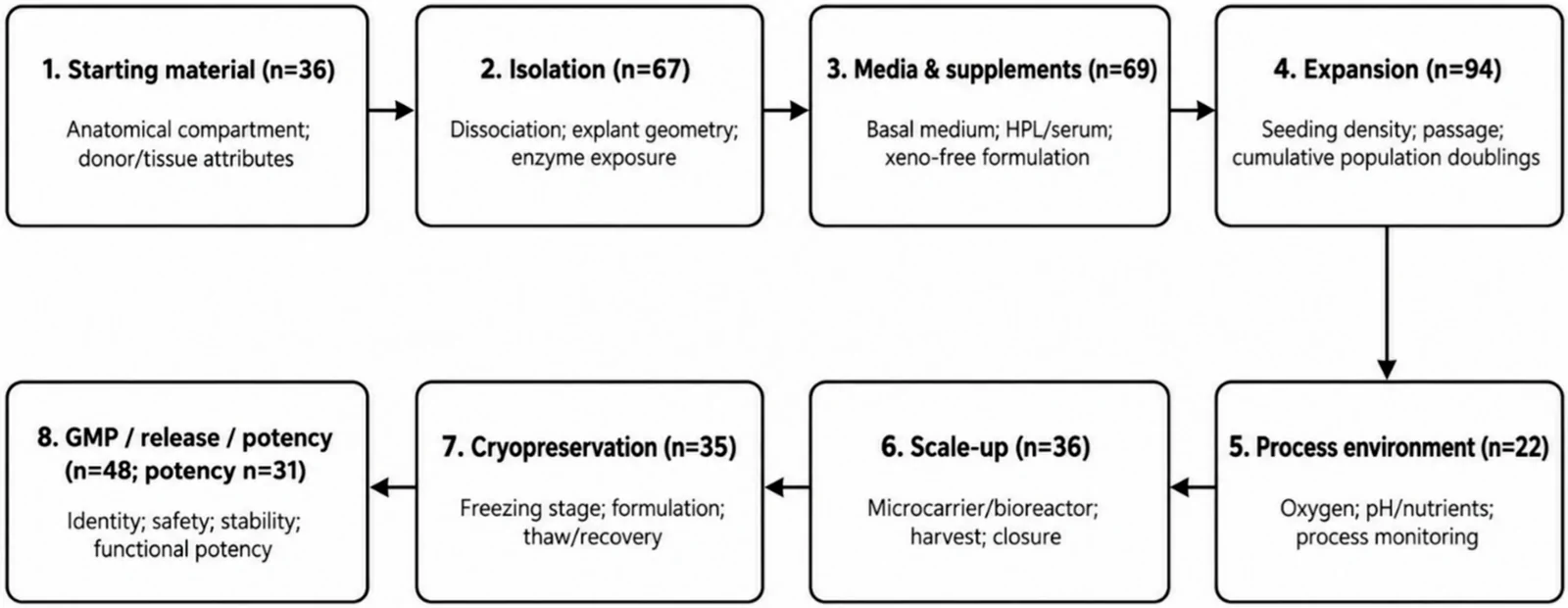
End-to-end manufacturing evidence map. Counts indicate the number of reports coded to each non-mutually exclusive manufacturing domain. Domain frequency represents evidence density and should not be interpreted as evidence strength, consistency, or formal criticality; product-specific evidence linking parameter variation to relevant CQAs is required before CMA/CPP designation.

The most frequent domains were expansion/passaging/senescence (94/238; 39.5%), media/supplements (69; 29.0%), isolation/tissue processing (67; 28.2%), GMP/quality/release (48; 20.2%), starting material/source (36; 15.1%), scale-up/3D/bioreactor (36; 15.1%), cryopreservation/storage (35; 14.7%), potency/functional CQA (31; 13.0%), and oxygen/process environment (22; 9.2%). Domain frequency was interpreted as evidence density and not as evidence strength, consistency, or criticality. A single report could contribute to several manufacturing domains; domain counts should therefore not be summed to estimate the number of independent studies. The distribution of evidence across manufacturing domains is shown in Figure 2.

### 3.4. Starting Material and Anatomical Compartment

The numerical statements reported in the process synthesis are source-mapped in Supplementary Table S7. A recurring full-text finding was that “umbilical cord” and “placenta” are insufficient manufacturing definitions. In direct comparison of umbilical cord zones, Wharton’s jelly yielded 4.61 ± 0.57 × 10^6^ fresh cells per centimeter of tissue, formed monolayers rapidly, and contained fewer contaminating populations than several alternative compartments [2]. Beeravolu et al. demonstrated reproducible separation of cord lining, Wharton’s jelly, the cord–placenta junction, and fetal placenta [1]. Placental compartment also affected product identity: Sardesai et al. showed that standard culture conditions could permit maternal-cell overgrowth, whereas controlled dissection and selective isolation improved reproducibility of fetal chorionic-villus MSC isolation [3]. Collectively, these observations identify anatomical compartment as a candidate material attribute warranting product-specific criticality assessment instead of being treated as a descriptive label.

### 3.5. Isolation and Primary Tissue Processing

No universal superiority of explant or enzymatic isolation was identified. Closed enzymatic processing in Smith et al. reduced open manipulations and generated more than a ten-fold increase in cells per centimeter compared with the preceding workflow [10], whereas enzyme-free whole-segment adhesion in De Bruyn et al. recovered MSCs from all 50 cords studied [13]. Enzyme-free homogenization produced initial yield and viability comparable with collagenase digestion but slower subsequent proliferation [12]. Lee et al. reported 3.92 ± 0.83 × 10^6^ cells/g with a 2–4 mm minimal-cube explant format, 10.2-fold greater than enzymatic digestion, with CD146 expression of 73.4% versus 25.7% [14]. In Wang et al., culture success was 37%, 37%, and 75% for explant, enzyme digestion, and enzyme-assisted explant workflows, respectively, while primary outgrowth occurred at 16.00 ± 3.65, 5.50 ± 2.08, and 4.71 ± 1.97 days [15]. Taken together, these studies suggest process-specific interactions among anatomical compartment, tissue geometry, enzyme composition, open/closed handling, and downstream culture; they do not identify a single ranked isolation method.

A 90-donor standardization study added operational information beyond small method comparisons: processing could begin up to 48 h after collection without evident loss of core MSC characteristics, vessel removal improved purity, and alpha-MEM with HPL improved reproducibility; reported purity was approximately 98.9% and viability exceeded 97% [20].

### 3.6. Culture Medium and Supplements

Twelve reports directly compared HPL with FBS. Human-derived and xeno-free systems frequently supported high proliferation, population doublings, viability, CFU-F activity, or process reproducibility [10,20,22,24,25,26,27], but effects were process-stage and formulation dependent. In the UCX ATMP process, Martins et al. found that replacing FBS with human serum or HPL reduced P0 yield at the isolation stage (*p* < 0.01), although the optimized clinical process subsequently achieved high recovery, viability, genetic stability, and immunomodulatory performance [33]. Swamynathan et al. reported a population-doubling time of 32.13 ± 3.95 h versus 45.24 ± 4.35 h with FBS and cumulative population doublings of 18.32 ± 0.38 versus 16.25 ± 0.26 in their serum-/xeno-free system [25]. These within-study findings do not define a universal HPL effect because basal medium, HPL source/pooling, concentration, heparin, seeding density, passage, and endpoint differ among processes.

### 3.7. Expansion, Passage, Senescence, and Process Environment

Expansion outcomes depended on seeding density, cumulative population doublings, passage number, culture duration, and process environment. Ten manufacturing reports directly compared hypoxia with normoxia; oxygen concentration, exposure duration, co-interventions, culture format, endpoints, and assessment timepoints varied substantially. A fixed passage number is therefore an incomplete process limit: the same passage can represent different cumulative population doublings and different histories of cell stress. For comparability, cumulative population doublings together with viability, senescence/genetic-stability markers, and product-relevant function provide a more informative process description than passage number alone.

### 3.8. Scale-Up, Microcarriers, and Bioreactors

Modern reports demonstrate technical scale-up across hollow-fiber, stirred-tank, rotating-bed, packed-bed, and microcarrier systems [29,30,31,32,33,34,35]. In a disposable rotating-bed bioreactor, Neumann et al. reported 24.6 ± 2.4 × 10^6^ cells after five days from a 3 × 10^6^-cell inoculum, corresponding to 8.2 ± 0.8-fold expansion and a population-doubling time of 39.6 ± 1.8 h; static-culture PDT was 31 h [34]. The result supports reproducible dynamic expansion, not faster growth than the static comparator. Osiecki et al. similarly demonstrated closed packed-bed isolation/expansion while placental MSC growth remained slower than matched 2D controls [35].

At a larger scale, a 3 L stirred-tank comparability study produced approximately 9.48 × 10^8^ cells over six days versus 9.73 × 10^8^ cells over eight days in planar culture, with viability above 90% and predefined CQAs maintained [31]. At 50 L, Soder et al. reported approximately 37 × 10^9^ cells during the seven-day 50 L expansion stage of a 13-day seed-train workflow, 27-fold expansion, and 95% harvest efficiency while maintaining phenotype, chromosomal stability, function, and sterility [32]. These studies demonstrate feasibility within the evaluated platforms but do not by themselves establish process superiority; harvest efficiency, degree of closure, CQA comparability, failed-batch frequency, and cost per released dose are also relevant.

### 3.9. Cryopreservation and Post-Thaw Comparability

Cryopreservation studies addressed three different intervention levels: starting-tissue cryopreservation, intermediate cell-bank freezing, and final-product thaw. These should not be treated as one common exposure. Serum-/xeno-free cryopreservation of cord tissue preserved post-thaw phenotype, immunosuppressive activity, and differentiation in one study [36]. A same-donor split model found that cryopreserved-tissue explantation yielded a purer initial population, while enriched cells from fresh-digestion and cryopreserved-explant pathways were similar in viability, growth, phenotype, and cytokine secretion [37]. Because the split pathways differed in both tissue preservation and isolation method, this comparison does not isolate the effect of freezing alone.

Long-term cell cryopreservation further showed that conventional identity measures can miss subtler changes. Zhang et al. reported cell count/viability of 1.00 × 10^7^/96.34% before cryopreservation versus 0.943 × 10^7^/93.81% after 12 months, with retained phenotype and normal karyotype but modest molecular changes [42]. Nguyen et al. reported high viability in freshly thawed and culture-rescued cells, but weaker T-cell suppression immediately after thaw [26]. The observation supports separate assessment of viability and immediate functional potency for off-the-shelf products. In Zhang et al., phenotype, differentiation, and karyotype were evaluated after re-expansion to passage 4; these measurements do not substitute for immediate post-thaw potency assessment [42].

### 3.10. GMP, CQAs, Potency, and Release

Frequently reported quality endpoints included identity phenotype, viability, sterility, mycoplasma, endotoxin, karyotype/genomic stability, and differentiation. Function-oriented assays included T-cell suppression, mixed lymphocyte reactions, immunomodulatory responses, angiogenic activity, cytokine/secretome profiles, and omics-based stability [20,25,26,27,31,32,44,45,46]. Across the corpus, quantitative mechanism-linked release thresholds and immediate post-thaw functional release testing were reported much less consistently than viability and identity. This gap is important because regulatory potency approaches are product-specific and are intended to reflect relevant biological activity instead of a generic MSC marker panel [8,9].

### 3.11. Direct Comparative Evidence

**Figure 3:**
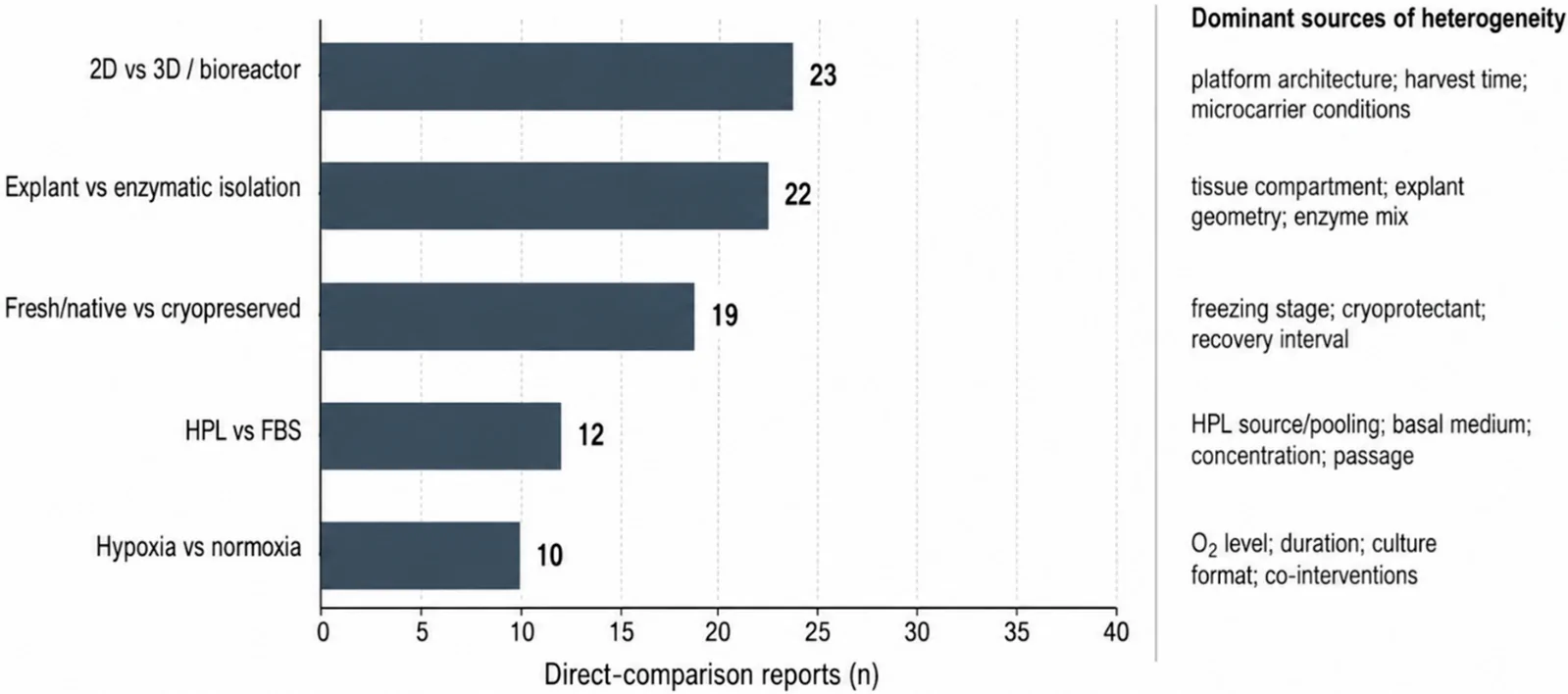
Direct comparative manufacturing evidence. Bar length indicates report count; annotations summarize the dominant biological, process, and methodological sources of heterogeneity. Counts describe evidence density at report level and are interpreted together with study-family handling, direction/consistency patterns, and design robustness.

The five recurrent manufacturing contrasts differed in tissue compartment, intervention definition, process stage, endpoint, unit, and assessment time. Comparative findings were therefore mapped descriptively by comparison family, outcome domain, direction/consistency pattern, and dominant effect modifiers (Table 3; Figure 3). Report counts are shown at report level. Linked-publication handling is detailed in Supplementary Table S5A, and corresponding study-family counts for the five direct-comparison categories are provided in Supplementary Table S5C.

Interpretive weight was greatest when the same donor or starting cell preparation was evaluated under defined alternative conditions with biological replication. Examples include the within-cord procedural comparisons of Smith et al. [10], the three-cord large-scale media comparison of Swamynathan et al. [25], and the paired thaw/recovery comparison of Nguyen et al. [26]. Such designs strengthen inference about the tested contrast, but small donor sets and co-interventions still limit generalization. The uncontrolled 50-cord isolation series of De Bruyn et al. [13] supports culture success within that workflow and does not establish superiority over enzymatic isolation. Endpoint-specific design features and denominators are presented in Supplementary Table S8.

### 3.12. Evidence-Informed Manufacturing Development Matrix

Table 4 translates the mapped relationships into evidence-informed development considerations. The term “candidate” is deliberate: the review identifies recurrent relationships that merit product-specific evaluation, whereas formal CMA/CPP designation requires risk assessment and demonstration that parameter variation affects relevant CQAs within a defined process [5,6].

## 4. Discussion

This review synthesizes 238 full-text manufacturing reports and reframes the literature as an interconnected control system, not a collection of isolated optimization experiments. The principal contribution is the end-to-end linkage of starting material, tissue processing, culture environment, expansion history, scale-up, cryopreservation, and release. Existing QbD analyses have highlighted the importance of process parameters in MSC expansion [5], and more recent work has mapped bioreactor-specific parameters and quality attributes [7]. The present synthesis extends that logic across the complete placenta/umbilical cord manufacturing chain, where source anatomy is itself a candidate material attribute and where clinically relevant post-thaw function must be considered separately from conventional identity and viability.

Differences among Wharton’s jelly, cord lining, perivascular regions, chorionic villi, decidua, and other compartments are not merely terminological. They can affect initial yield, purity, maternal/fetal composition, and growth behavior [1,2,3]. Manufacturing descriptions should therefore specify the anatomical source operationally, including dissection region, tissue amount, vascular structures, transport interval, and acceptance criteria. Without this control, downstream comparisons of media or expansion platforms may be confounded by starting-material differences.

The direct isolation literature does not support a universal ranking of explant and enzymatic methods. Closed enzymatic workflows can improve throughput and reduce open handling [10], whereas explant-based approaches can improve culture success or preserve particular phenotypes under specific compartment and geometry conditions [13,14,15]. Enzyme composition, explant size, anatomical compartment, contamination control, and downstream culture act as interacting process elements. The practical unit of comparison is therefore the complete isolation-to-expansion workflow, not the isolation label alone.

HPL, human serum, and defined/xeno-free media can support clinically relevant expansion, but the direct evidence does not justify a universal formulation-independent claim of HPL superiority. HPL source and pooling, basal medium, concentration, heparin use, seeding density, passage, and endpoint differ among studies. Martins et al. additionally showed that a supplement suitable for later clinical expansion may reduce early isolation yield within a specific workflow [33]. Raw-material qualification and lot control should therefore be linked to the intended process stage and CQA profile instead of being judged by proliferation alone.

The mapped evidence supports cumulative population doublings as a more informative descriptor of expansion history than passage number alone. Scale-up studies demonstrate that clinically relevant outputs can be achieved in closed or functionally closed systems [29,30,31,32,33,34,35], but absolute yield is an incomplete performance metric. Platform development should also consider harvest recovery, process duration, degree of closure, mass-transfer environment, CQA comparability, operator burden, failed-batch frequency, and cost per released dose. These considerations are consistent with the broader QbD principle that process understanding requires a demonstrated relationship between controlled parameters and product quality [6].

Starting-tissue freezing, intermediate bank freezing, and final-product thaw have different objectives and failure modes. Surface phenotype and viability can remain acceptable while molecular or functional state changes [41,42]. For an off-the-shelf final product, immediate post-thaw function is the clinically relevant assessment point. The weaker T-cell suppression reported immediately after thaw despite high viability [26] illustrates why viability cannot be assumed to represent potency. Recovery-culture assays may not accurately represent immediate functional readiness when the clinical product is administered directly after thaw.

A recurrent translational gap across the mapped literature is the limited use of validated, mechanism-linked, stability-indicating assays with predefined acceptance thresholds. T-cell suppression, mixed lymphocyte reactions, IDO-related responses, angiogenic assays, cytokine profiles, clonogenicity, and omics signatures interrogate different mechanisms. Regulatory guidance similarly treats potency as product-specific and linked to biological activity [8,9]. Surface phenotype supports identity but does not establish therapeutic equivalence [4]. For perinatal MSCs, potency strategies should therefore be developed around the intended mechanism of action and be sensitive to manufacturing perturbations, storage, and clinically relevant post-thaw conditions.

Collectively, the mapped evidence supports an interdependent manufacturing-development model and does not select a universal process. An evidence-informed development strategy would include an explicitly defined anatomical starting material, donor/tissue qualification and transport limits, standardized dissection/isolation, qualified clinical-grade media, defined seeding density and expansion limits, scalable closed processing, stage-specific cryopreservation/thaw assessment, and release testing that integrates identity, purity, viability, microbiological safety, genetic stability, and mechanism-relevant potency. Candidate material attributes and process parameters identified by literature mapping warrant product-specific risk assessment and experimental confirmation of their relationship to CQAs before formal criticality designation.

**Strengths and limitations**

Strengths include predefined review methods, independent duplicate screening with calibrated eligibility criteria, independent verification of charted data, PRESS-reviewed search strategies, complete record-level accounting for the primary Scopus/Web of Science route, transparent reporting of supplementary search contributions, complete retrieval of all full texts sought for assessment, explicit study-family handling, structured design-robustness characterization of the detailed comparative evidence, and a synthesis approach that separates evidence density from evidence strength and formal parameter criticality. The review also distinguishes starting-tissue cryopreservation, intermediate banking, and final-product thaw and avoids combining manufacturing comparisons that represent non-equivalent process questions.

This review has limitations at both the evidence-base and review-design levels. Experimental definitions, tissue compartments, passage/timepoints, units, and outcome definitions were heterogeneous, and many studies were small or single-center. Donor and batch denominators, failed-batch rates, raw-material lot effects, independent process transfer, quantitative potency thresholds, and cost per released dose were inconsistently reported.

At the review-design level, Embase was not searched, so eligible reports accessible through its database-specific coverage or indexing may have been missed despite complementary searches. Conference abstracts lacking sufficient manufacturing detail were ineligible, although conference records could have helped identify eligible full reports. Missing eligible sources could affect manufacturing-domain frequencies, apparent evidence gaps, and the interpretation of individual comparison families. The extent of any omission and its impact remain unknown. Domain frequencies therefore describe the included corpus; apparent gaps should not be interpreted as confirmed absence of research, and conclusions are confined to relationships supported within the mapped evidence. The direction of any resulting bias cannot be inferred from the present corpus.

Complete identification, screening, and full-text stage counts were not archived for the supplementary PubMed/MEDLINE search and citation-searching routes; these supplementary routes are therefore reported only by their verified unique eligible additions instead of reconstructed complete PRISMA branches. The focused source-level design table covers 14 key reports supporting the detailed synthesis; design characterization does not constitute formal critical appraisal or a certainty grade across all 238 reports. Incompletely specified donor/batch denominators, uncertain pairing, and technical repeats limit interpretation of some comparative findings. These limitations constrain certainty about cross-center comparability and the transfer of laboratory optimization findings into validated clinical or commercial manufacturing.

## 5. Conclusions

Perinatal MSC manufacturing is best understood as an interconnected control system. Across 238 full-text manufacturing reports, anatomical starting material, isolation workflow, media and supplements, expansion history, process environment, scale-up, cryopreservation stage, and release testing were associated with differences in process performance and reported product attributes. The evidence does not support a universal isolation method, culture supplement, or expansion platform. Product development should define the anatomical source, control the isolation-to-expansion history, report cumulative population doublings, evaluate scale-up and cryopreservation at the intended process stage, and link release to product-relevant functional potency. Future work should prioritize cross-center process transfer, failed-batch denominators, mechanism-linked potency thresholds, immediate post-thaw function, and cost per released dose.

## Figures and Tables

**Table 1:** PCC framework and operational definitions.

PCC Element	Operational Definition	Examples
Population	Human MSCs derived from placenta or umbilical cord tissues with a defined anatomical source or compartment.	Wharton’s jelly, cord lining/matrix, perivascular tissue, amnion, chorion, chorionic villi, decidua, cord–placenta junction.
Concept	Manufacturing variables and their relationship with process outcomes, candidate CMAs/CPPs, CQAs, potency, or release testing.	Dissection, enzyme exposure, medium/supplement, seeding density, passage/CPD, oxygen, microcarriers, bioreactors, cryopreservation/thaw, release assay.
Context	Experimental, translational, GMP, cell-bank, and clinical-manufacturing settings in any country.	Research-scale optimization, comparability, scale-up, clinical-grade manufacturing.

**Table 2:** Characteristics of the included manufacturing corpus (n = 238).

Characteristic	Category	n (%)
Publication period	2004–2010	21 (8.8%)
	2011–2015	75 (31.5%)
	2016–2020	74 (31.1%)
	2021–2026	68 (28.6%)
Primary tissue source	Umbilical cord/Wharton’s jelly	183 (76.9%)
	Placenta/amnion/chorion/decidua	46 (19.3%)
	Multiple perinatal sources	9 (3.8%)
Analytical unit	Included reports	238
	Distinct study families in sensitivity set	236
Manufacturing domain	Expansion/passaging/senescence	94 (39.5%)
	Media/supplements	69 (29.0%)
	Isolation/tissue processing	67 (28.2%)
	GMP/quality/release	48 (20.2%)
	Starting material/source	36 (15.1%)
	Scale-up/3D/bioreactor	36 (15.1%)
	Cryopreservation/storage	35 (14.7%)
	Potency/functional CQA	31 (13.0%)
	Oxygen/process environment	22 (9.2%)

**Table 3:** Structured synthesis of direct manufacturing comparisons.

Contrast	Reports	Principal Outcomes	Dominant Modifiers	Direction/Consistency Pattern
2D vs. 3D/bioreactor	23	Yield, fold expansion, PDT, harvest recovery, CQA comparability	Platform architecture, microcarrier, mixing/mass transfer, harvest time, matched control	Feasibility was demonstrated across evaluated platforms, but productivity and CQA differences were platform-specific and not directionally uniform.
Explant vs. enzymatic isolation	22	Primary yield, culture success, outgrowth time, phenotype	Anatomical compartment, explant geometry, enzyme identity/concentration, hybrid workflows	Mixed; neither approach was consistently superior across yield, outgrowth time, culture success, and phenotype.
Fresh/native vs. cryopreserved	19	Yield, viability, growth, phenotype, function	Tissue vs. cell-bank vs. final-product freezing, cryoprotectant, storage duration, recovery period	Identity/viability were often preserved, whereas growth, function, and recovery depended on cryopreservation stage and recovery interval.
HPL vs. FBS	12	Proliferation, PDT, yield, CFU-F, phenotype/function	HPL pooling/source, basal medium, concentration, heparin, seeding density, passage	Human-derived supplementation often supported robust expansion, but superiority over FBS was not consistent across formulations and process stages.
Hypoxia vs. normoxia	10	Proliferation, phenotype, senescence, immunomodulatory function	Oxygen level, duration, culture format, co-interventions, endpoint/timepoint	Mixed and context-dependent across proliferation, senescence, phenotype, and immunomodulatory outcomes.

**Table 4:** Evidence-informed manufacturing development matrix.

Manufacturing Stage	Candidate Control Variable	Process/CQA Relationship Supported by Mapped Evidence	Evidence-Informed Development Consideration
Starting material	Anatomical compartment; tissue amount; transport/processing interval; donor/tissue attributes	Source compartment is associated with primary yield, purity, maternal/fetal composition, and growth behavior.	Consider operational specification of the anatomical source and product-specific acceptance criteria for starting material.
Isolation	Dissection; explant geometry; enzyme identity/concentration/exposure; open/closed handling	Isolation conditions affect yield, time to outgrowth, culture success, phenotype, and contamination risk.	Evaluate the complete isolation workflow rather than ranking explant and enzymatic methods independently of source and downstream culture.
Media/supplements	Basal medium; HPL/serum source, pooling, concentration; xeno-free formulation; heparin	Supplement choice can affect isolation yield, proliferation, reproducibility, and functional readouts.	Qualify raw materials and lots where relevant, and assess media effects across the complete process rather than short-term proliferation alone.
Expansion/environment	Seeding density; passage policy; cumulative population doublings; oxygen; culture duration	Expansion history influences proliferation, senescence, genetic stability, and function.	Assess CPD together with relevant CQA trends when defining process limits; passage number alone may be insufficient.
Scale-up	Bioreactor architecture; microcarrier; agitation/mass transfer; inoculation; harvest timing/recovery	Large-scale cell output is feasible while growth rate and CQA comparability remain platform-dependent.	Establish product-specific evidence for scale-down models, process comparability, harvest recovery, and transferability before inferring platform equivalence.
Cryopreservation/thaw	Freezing stage; formulation; cooling/storage; thaw; recovery interval	Phenotype/viability can be preserved while immediate functional potency changes.	Evaluate cryopreservation at the intended manufacturing stage and assess clinically relevant post-thaw function.
Release/potency	Identity, purity, viability, sterility, genomic stability, product-relevant potency	Generic phenotype does not establish functional equivalence; quantitative release thresholds remain incompletely harmonized.	Consider mechanism-linked, stability-indicating potency assays and product-specific acceptance ranges for release.

## Data Availability

All data supporting the findings of this review are available within the article and its Supplementary Materials.

## Supplementary Materials

The following supporting information can be downloaded at: https://www.mdpi.com/article/10.3390/life16101740/s1, Table S1A: Supplementary review methods; Table S1B: Operational definitions for evidence-characterization domains; Table S2: Search strategies; Table S3: Bibliographic and manufacturing-domain characteristics of all 238 included reports; Table S4A: Distribution of full-text exclusions by primary reason (n = 208); Table S4B: Report-level full-text exclusions (n = 208). Table S5A: Linked reports and study-family sensitivity handling; Table S5B: Manufacturing-domain frequencies at report and study-family levels; Table S5C: Direct-comparison counts at report and study-family levels; Table S6: Direct-comparison mapping and heterogeneity assessment; Table S7: Source-mapped quantitative audit for statements cited in the main process synthesis; Table S8: Source-level experimental-design features of key reports supporting the detailed process synthesis.

## Abbreviations

2D	Two-dimensional
3D	Three-dimensional
alpha-MEM	Alpha modification of Minimum Essential Medium
ATMP	Advanced therapy medicinal product
CD146	Cluster of differentiation 146
CFU-F	Colony-forming unit–fibroblast
CMA	Critical material attribute
CPD	Cumulative population doublings
CPP	Critical process parameter
CQA	Critical quality attribute
DOI	Digital object identifier
FBS	Fetal bovine serum
GMP	Good Manufacturing Practice
HPL	Human platelet lysate
IDO	Indoleamine 2,3-dioxygenase
ISCT	International Society for Cellular Therapy
MEDLINE	Medical Literature Analysis and Retrieval System Online
MSC	Mesenchymal stromal cell
P0	Passage 0
PCC	Population–Concept–Context
PDT	Population doubling time
PRESS	Peer Review of Electronic Search Strategies
PRISMA	Preferred Reporting Items for Systematic Reviews and Meta-Analyses
PRISMA-ScR	Preferred Reporting Items for Systematic Reviews and Meta-Analyses Extension for Scoping Reviews
PRISMA-S	Preferred Reporting Items for Systematic Reviews and Meta-Analyses literature search extension
QbD	Quality by Design
SWiM	Synthesis Without Meta-analysis
UCX	Human umbilical cord tissue-derived mesenchymal stromal cells
