# Supplementary material for: Manufacturing of Placenta- and Umbilical Cord-Derived Mesenchymal Stromal Cells: A Systematic Scoping Review of Process Parameters, Quality Attributes, and Evidence-to-Control Relationships
Source: Life (Basel). 2026 Oct 16;16(10):1740. doi: 10.3390/life16101740 (PMC13706292; doi:10.3390/life16101740)
Supplement: Supplementary file 1 [file life-16-01740-s001.zip › life-4575915-supplementary.pdf]

Supplementary Materials

Table S1A. Supplementary review methods.

Methods element	Review procedure
Design	Systematic scoping review of human placenta- and umbilical cord-derived MSC manufacturing evidence.
PCC—Population	Human MSCs from placenta, umbilical cord, Wharton's jelly, cord lining/matrix/perivascular regions, cord-placenta junction, amnion, chorion, chorionic villi, or decidua.
PCC—Concept	Manufacturing variables, process outcomes, candidate CMAs/CPPs, CQAs, potency, stability, and release testing.
PCC—Context	Experimental, translational, GMP, cell-bank, and clinical-manufacturing settings in any country.
Eligibility focus	A manufacturing variable, process-development parameter, stability intervention, GMP workflow, or release/CQA procedure had to be a principal study variable with at least one extractable process or quality outcome.
Full-text assessment	All 439 reports sought for retrieval were obtained and reviewed in full text; no report was excluded because the complete article was unavailable. All 238 included reports were verified against the complete article; supplementary files were used where relevant.
Context-only literature	Biological/mechanistic studies without an eligible manufacturing-process endpoint were excluded from the formal corpus and used only as background literature where relevant.
Charting	A piloted standardized charting form covered citation, year, study design, tissue source, manufacturing stage/domain, comparator status, study-family linkage, GMP context, principal outcome, and, where relevant, process parameters, CQAs, potency, release, and validation.
Missing data	NR indicates not reported in the full-text source. Numerical values were not imputed and missing means/SDs were not reconstructed.
Study-family handling	Potential companion reports were independently assessed by two reviewers using predefined author, source, platform, donor/batch, period, recruitment, passage-series, and explicit-linkage criteria. Disagreements were resolved by consensus or third-reviewer adjudication. The study-family sensitivity set contained 236 families. The manufacturing-domain ranking and counts for the five direct-comparison categories were unchanged (Tables S5B and S5C).
Evidence characterization	The full corpus was mapped descriptively by manufacturing domain and outcome. Detailed design-robustness characterization was concentrated on direct comparative and quantitative reports; source-level comparator, matching, and biological-unit descriptors for 14 key reports are summarized in Table S8. No composite quality or certainty score was used.
Quantitative synthesis	Comparative findings were mapped by manufacturing contrast and outcome domain. Pooling was reserved for situations with biological, process, and methodological comparability sufficient for an interpretable common effect estimate; relevant SWiM reporting elements were used when pooling was inappropriate.
Non-English reports	No language filter was applied. Potentially eligible non-English reports were translated for full-text assessment, with eligibility decisions and extracted data verified against the original-language source.

Abstract-only records	Abstract-only records were excluded when they did not provide sufficient process detail to verify manufacturing conditions and extract prespecified outcomes.
Search peer review	Electronic strategies were developed with an information specialist and independently peer-reviewed using the PRESS framework before execution.
Record management/software	Search exports and screening decisions were maintained in a version-controlled Microsoft Excel for Microsoft 365 review database. DOI/title normalization, consistency checks, and reproducible analysis were implemented in Python 3.x.
Calibration	Before formal screening, two reviewers independently applied the eligibility criteria to a calibration sample. Ambiguities were resolved before full screening; prespecified acceptable agreement was achieved and Cohen's kappa indicated strong agreement.
Selection process	Two reviewers independently screened titles/abstracts and full texts. Records considered potentially eligible by either reviewer advanced to full text. Disagreements were resolved by consensus, with third-reviewer adjudication when required.
Charting verification	Data were charted by one reviewer and independently verified against the full-text source by a second reviewer. A random subset was independently double-extracted before full extraction to calibrate the framework; discrepancies were resolved by consensus.
Deduplication verification	Potential duplicates identified through DOI, normalized title/year, or fuzzy title matching were manually verified against authorship, year, journal, DOI, tissue source, and study characteristics before removal.
Author contact	No author contact was required to determine eligibility or verify numerical results used in the primary synthesis.
Design-robustness assessment	Formal source-level critical appraisal was not undertaken. Direct comparative and detailed quantitative reports underwent independent two-reviewer characterization of design robustness using biological replication, comparator definition, same-donor/matched design, outcome completeness, statistical reporting, process reproducibility, CQA/potency assessment, post-thaw assessment, and independent validation. These characteristics informed narrative weighting rather than eligibility and were not combined into a composite quality or certainty score. Table S8 makes these design features explicit for the 11 studies in the quantitative audit (Table S7) and three additional comparative examples. Non-comparative observations support process feasibility; they do not establish relative superiority.
Gray/regulatory sources	Gray-literature and regulatory/public manufacturing sources were used as supplementary discovery/context sources. Formal inclusion required a retrievable primary report with sufficient manufacturing detail for source verification and extraction.
Database-coverage limitation	Embase was not searched. Eligible reports may therefore have been missed despite the complementary search routes. The magnitude and direction of any effect on domain frequencies, apparent evidence gaps, or comparison-family interpretation were not quantified (main text, Strengths and Limitations Section).

Table S1B. Operational definitions for evidence-characterization domains.

Domain	Operational definition	Coding rule	Possible values
Biological replication	Explicit biological units supporting the reported process observation.	Code from reported donor, tissue, batch, or independent biological replicate information. Technical repeats, wells, and counting aliquots do not increase independent donor/batch n; overall study n is distinguished from endpoint-level n.	Reported/NR
Direct comparator	Within-study comparison of two or more manufacturing conditions.	Code yes only when comparator conditions are evaluated in the same report.	Yes/No
Quantitative process outcome	Numerical process-performance measure.	Includes yield, growth rate, PDT, CPD, viability, harvest recovery, culture success, or process duration.	Yes/No
CQA assessment	Assessment of a product-quality attribute relevant to identity, purity, safety, stability, or function.	Code when at least one prespecified CQA is measured.	Yes/No
GMP context	Manufacturing explicitly described as GMP-compliant, clinical-grade, or conducted within a regulated manufacturing workflow.	Code from the authors' stated manufacturing context.	Yes/No
Release threshold	Predefined quantitative or categorical acceptance criterion used for release or process disposition.	Code only when an explicit threshold/range is reported.	Reported/NR
Potency testing	Functional or mechanism-relevant assay intended to reflect biological activity.	Code when potency-related functional testing is reported beyond generic identity phenotype.	Yes/No
Post-thaw assessment	Quality or functional testing after thaw at an explicitly defined timepoint or recovery interval.	Distinguish immediate post-thaw testing from recovery-culture testing when reported.	Immediate/ Recovery/ Both/No
Independent validation	Reproduction or confirmation of a process relationship in a separate donor/batch set, validation run, center, or explicitly independent experimental series.	Code only when independence is explicit in the report.	Yes/No/ Unclear

Design robustness	Degree to which a direct comparison minimizes biological and process confounding.	Prioritize same-donor paired or matched-process comparisons over unmatched cross-donor, cross-center, or cross-platform comparisons when interpreting direction and consistency. A non-comparative process-development series supports feasibility within the tested workflow, without supporting a claim of superiority over another process. Source-level examples are provided in Table S8.	Same-donor paired/ Matched/ Independent unmatched/ Unclear/Non-comparative

Table S2. Search strategies

Source	Platform	Exact strategy/method	Restrictions	Source-level accounting/final contribution
Scopus	Elsevier Scopus	TITLE-ABS-KEY(("mesenchymal stromal cell*" OR "mesenchymal stem cell*" OR "multipotent stromal cell*" OR MSC OR MSCs) AND (placenta* OR placental OR amnion* OR chorion* OR decidua* OR "umbilical cord" OR "Wharton's jelly" OR "Wharton jelly" OR "cord lining" OR "cord matrix" OR "cord-placenta junction" OR perivascular OR subamnion*) AND (manufactur* OR process* OR isolation OR explant* OR enzymatic OR digestion OR expansion OR culture* OR medium OR "platelet lysate" OR xeno-free OR serum-free OR hypoxi* OR passage* OR scale-up OR microcarrier* OR bioreactor* OR cryopreserv* OR thaw* OR GMP OR "good manufacturing practice" OR potency OR "quality control" OR "release test*" OR "batch release"))	No date or language filter in query	11,157 records identified before cross-source deduplication.
Web of Science Core Collection	Clarivate Web of Science	TS=(("mesenchymal stromal cell*" OR "mesenchymal stem cell*" OR "multipotent stromal cell*" OR MSC OR MSCs) AND (placenta* OR placental OR amnion* OR chorion*	No date or language filter in query	7,703 records identified before cross-source deduplication.

		OR decidua* OR "umbilical cord" OR "Wharton's jelly" OR "Wharton jelly" OR "cord lining" OR "cord matrix" OR "cord-placenta junction" OR perivascular OR subamnion*) AND (manufactur* OR process* OR isolation OR explant* OR enzymatic OR digestion OR expansion OR culture* OR medium OR "platelet lysate" OR xeno-free OR serum-free OR hypoxi* OR passage* OR scale-up OR microcarrier* OR bioreactor* OR cryopreserv* OR thaw* OR GMP OR "good manufacturing practice" OR potency OR "quality control" OR "release test*" OR "batch release"))		
PubMed	NCBI PubMed	(("Mesenchymal Stem Cells"[Mesh] OR "mesenchymal stromal cell*"[tiab] OR "mesenchymal stem cell*"[tiab] OR "multipotent stromal cell*"[tiab]) AND ("Placenta"[Mesh] OR "Umbilical Cord"[Mesh] OR "Wharton Jelly"[Mesh] OR placenta*[tiab] OR placental[tiab] OR amnion*[tiab] OR chorion*[tiab] OR decidua*[tiab] OR "umbilical cord"[tiab] OR "Wharton's jelly"[tiab] OR "Wharton jelly"[tiab] OR "cord lining"[tiab] OR "cord matrix"[tiab] OR "cord-placenta junction"[tiab] OR perivascular[tiab] OR subamnion*[tiab]) AND (manufactur*[tiab] OR process*[tiab] OR isolation[tiab] OR explant*[tiab] OR enzymatic[tiab] OR digestion[tiab] OR expansion[tiab] OR culture*[tiab] OR medium[tiab] OR "platelet lysate"[tiab] OR xeno-free[tiab] OR serum-free[tiab] OR hypoxi*[tiab] OR passage*[tiab] OR scale-up[tiab] OR microcarrier*[tiab] OR bioreactor*[tiab] OR cryopreserv*[tiab] OR thaw*[tiab] OR GMP[tiab] OR "good manufacturing practice"[tiab] OR potency[tiab] OR "quality control"[tiab] OR "release testing"[tiab] OR "batch release"[tiab])) NOT (animals[mh] NOT humans[mh])	Human filter only through NOT animals-only expression	Two reports in the final corpus were identified uniquely through this supplementary PubMed search after deduplication against the primary Scopus/Web of Science corpus. This is the additional contribution, not the total number of PubMed records retrieved; complete stage-level counts were not archived.

Backward and forward citation searching	Reference lists/related publications	Prespecified backward and forward citation searching of eligible reports meeting predefined GMP/release, HPL/xeno-free, cryopreservation, and scale-up criteria; reference lists and related-publication links were screened using the same eligibility criteria.	No additional date or language restriction	Five reports in the final corpus were identified uniquely through citation searching after deduplication. This is the additional contribution, not the total number assessed; complete stage-level counts were not archived.

Table S3. Bibliographic and manufacturing-domain characteristics of all 238 included reports.

Author (year)	Title	Journal/source	Tissue source	Manufacturing domain(s)	DOI
Arulgunasekaran, Ruban Hentry (2026)	Tamarindus indica L. Seed Kernel Extract as a Sustainable Xenofree Coating for Human Umbilical Cord-Derived Mesenchymal Stem Cell Expansion: Comprehensive Structural, Functional, and Biocompatibility Evaluation for Optimized Stem Cell Culture	*Journal of Polymers and the Environment*	Umbilical cord/ Wharton's jelly	Media/supplements; Expansion/passages/senescence; Potency/functional CQA	10.1007/s10924-026-03788-5
Lee, Min Ji (2026)	Comparative Transcriptomic Profiling of Mesenchymal Stem Cells from Distinct Tissue Origins and Isolation Methods Highlights the Stability and Immunomodulatory Signature of	*Tissue Engineering and Regenerative Medicine*	Umbilical cord/ Wharton's jelly	Starting material/source; Isolation/tissue processing; Potency/functional CQA	10.1007/s13770-025-00765-2

	Umbilical Cord-Derived Smumf Cells				
Huang, Tse-Pu (2026)	Identifying key bioprocess variables using explainable machine learning to enhance culture efficiency and viability of umbilical cord-derived mesenchymal stem cells	*International Journal of Medical Sciences*	Umbilical cord/ Wharton's jelly	Expansion/passages/senescence	10.7150/ijms.127764
Biondo, Mattia (2026)	Isolation protocols and comparative characterization of human mesenchymal stem cells derived from amnion and umbilical cord for regenerative medicine applications: a monocentric study	*Placenta*	Multiple perinatal sources	Starting material/source; Isolation/tissue processing	10.1016/j.placenta.2026.04.026
Sheikh, Akram (2026)	The Efficacy of Umbilical Cord (UC) Serum, Human Platelet Lysate, and the Synergistic Effect of Nano-curcumin and Crocin as Supplements in the Proliferation and Survival of Human UC-derived Mesenchymal Stromal Cells (MSCs)	*Current Stem Cell Research & Therapy*	Umbilical cord/ Wharton's jelly	Media/supplements; Expansion/passages/senescence	10.2174/011574888x372692250918112149

Pan, Ying (2025)	Alternating 2D and 3D culture reduces cell size and extends the lifespan of placenta-derived mesenchymal stem cells	*Frontiers in Bioengineering and Biotechnology*	Placenta/ amnion/ chorion/ decidua	Scale-up/3D/bioreactor	10.3389/fbioe.2025.1632810
Babu, Seetha S. (2025)	Bioactive human platelet lysate gel for enhanced proliferation of human umbilical cord tissue derived mesenchymal stem cells	*Cell and Tissue Banking*	Umbilical cord/ Wharton's jelly	Media/supplements; Expansion/passages/senescence	10.1007/s10561-025-10175-2
Copuroglu, Mehmet (2025)	Clinical feasibility and cost efficiency of perinatal mesenchymal stem cell production under GMP conditions	*Scientific Reports*	Multiple perinatal sources	GMP/quality/release	10.1038/s41598-025-32738-9
Canas-Arboleda, Mariana (2025)	Comprehensive analysis of secretome and transcriptome stability of Wharton jelly mesenchymal stromal cells during good manufacturing practice-compliant production	*Cytotherapy*	Umbilical cord/ Wharton's jelly	GMP/quality/release; Potency/functional CQA	10.1016/j.jcyt.2024.08.008
Copuroglu, Mehmet (2025)	Comprehensive evaluation of the clinical feasibility of using perinatal medical waste as a source for fetal mesenchymal stem cell	*Cell and Tissue Banking*	Multiple perinatal sources	Starting material/source; Cryopreservation/storage; GMP/quality/release	10.1007/s10561-025-10204-0

	banking under good manufacturing practice conditions				
Soder, Rupal P. (2025)	Large Scale Manufacturing of Wharton's Jelly Mesenchymal Stromal Cells for Clinical Use	*Stem Cells International*	Umbilical cord/ Wharton’s jelly	Scale-up/3D/bioreactor; GMP/quality/release	10.1155/sci/5167739
Chu, Wanglong (2025)	Optimization of Culture Media for Human Umbilical Cord-Derived Mesenchymal Stem Cell Production	*Stem Cells International*	Umbilical cord/ Wharton’s jelly	Media/supplements; GMP/quality/release	10.1155/sci/4806605
Svajger, Urban (2025)	Pooled umbilical cord-derived MSCs retain key phenotypic and immunomodulatory properties – A path toward consistent manufacturing	*Tissue & Cell*	Umbilical cord/ Wharton’s jelly	GMP/quality/release; Potency/functional CQA	10.1016/j.tice.2025.103036
Wang P. (2025)	Quercetin enhances the cold storage effect on human umbilical cord mesenchymal stem cells in Celsior solution	*Cryobiology*	Umbilical cord/ Wharton’s jelly	Cryopreservation/storage	10.1016/j.cryobiol.2025.105299
Dewi, Sianty (2025)	Rapid isolation and characterization of Wharton's jelly-derived mesenchymal stem cells maintained in	*Universa Medicina*	Umbilical cord/ Wharton’s jelly	Isolation/tissue processing; Media/supplements	10.18051/univmed.2025.v44.65-72

	fresh-prepared human AB-serum				
Desterke, Christophe (2025)	Strong Transcriptional Signature of Mesenchymal Stromal Cells from Perinatal Tissues Compared with Adult Bone Marrow Sources Independent of Donors or Culture Conditions	*Stem Cells and Development*	Multiple perinatal sources	Starting material/source	10.1177/15473287251388411
Chu, Wanglong (2024)	A GMP-compliant manufacturing method for Wharton's jelly-derived mesenchymal stromal cells	*Stem Cell Research & Therapy*	Umbilical cord/ Wharton’s jelly	GMP/quality/release	10.1186/s13287-024-03725-0
Niknam B. (2024)	A Simple High Yield Technique for Isolation of Wharton's Jelly-derived Mesenchymal Stem Cell	*Avicenna Journal of Medical Biotechnology*	Umbilical cord/ Wharton’s jelly	Isolation/tissue processing	10.18502/ajmb.v16i2.14860
Bzinkowska, Aleksandra (2024)	Assessment of the Dose-Dependent Effect of Human Platelet Lysate on Wharton's Jelly-Derived Mesenchymal Stem/Stromal Cells Culture for Manufacturing Protocols	*Stem cells and Cloning: Advances and Applications*	Umbilical cord/ Wharton’s jelly	Media/supplements; GMP/quality/release	10.2147/sccaa.s471118
Couto, Pedro Silva (2024)	Biological differences between adult and perinatal human	*Cytotherapy*	Umbilical cord/ Whart	Starting material/source; Expansion/passages/senescence; GMP/quality/release;	10.1016/j.jcyt.2024.05.020

	mesenchymal stromal cells and their impact on the manufacturing processes		on’s jelly	Potency/functional CQA	
Lopez-Fernandez, Alba (2024)	Comparability exercise of critical quality attributes of clinical-grade human mesenchymal stromal cells from the Wharton's jelly: single-use stirred tank bioreactors versus planar culture systems	*Cytotherapy*	Umbilical cord/ Wharton’s jelly	Scale-up/3D/bioreactor; GMP/quality/release	10.1016/j.jcyt.2023.08.008
Hassan, Muhammad Najib Fathi (2025)	Comparing the growth kinetics and characteristics of Wharton's jelly derived mesenchymal stem cells expanded using different culture mediums	*Cytotechnology*	Umbilical cord/ Wharton’s jelly	Media/supplements	10.1007/s10616-024-00682-7
Zhu, Shuoji (2024)	Effect of chitin-architected spatiotemporal three-dimensional culture microenvironments on human umbilical cord-derived mesenchymal stem cells	*Bioactive Materials*	Umbilical cord / Wharton’s jelly	Scale-up/3D/bioreactor	10.1016/j.bioactmat.2024.01.014
Pingping Z. (2024)	Effects of spheroid culture in a rotating bioreactor on secretion of	*Chinese Journal of Tissue Engineering Research*	Placenta/ amnion/ chorion/	Scale-up/3D/bioreactor	10.12307/2024.177

	inflammatory factors by human placenta-derived mesenchymal stem cells		decidua		
Zhao, Lan (2024)	Evaluation of the impact of customized serum-free culture medium on the production of clinical-grade human umbilical cord mesenchymal stem cells: insights for future clinical applications	*Stem Cell Research & Therapy*	Umbilical cord/ Wharton's jelly	Media/supplements; GMP/quality/release	10.1186/s13287-024-03949-0
Rajput, Shafiqa Naeem (2024)	Expansion of human umbilical cord derived mesenchymal stem cells in regenerative medicine	*World Journal of Stem Cells*	Umbilical cord/ Wharton's jelly	Expansion/passages/senescence	10.4252/wjsc.v16.i4.410
Shokati, Ameneh (2024)	Good manufacturing practices production of human placental derived mesenchymal stem cells for therapeutic applications: focus on multiple sclerosis	*Molecular Biology Reports*	Placenta/ amnion/ chorion/ decidua	GMP/quality/release	10.1007/s11033-024-09372-1
Nakao, Mitsuyoshi (2024)	Harvesting methods of umbilical cord-derived mesenchymal stem cells from culture modulate cell properties and functions	*Regenerative Therapy*	Umbilical cord/ Wharton's jelly	Expansion/passages/senescence; Potency/functional CQA	10.1016/j.reth.2024.05.010

Lopez-Fernandez, Alba (2024)	Identification of critical process parameters for expansion of clinical grade human Wharton's jelly-derived mesenchymal stromal cells in stirred-tank bioreactors	*Biotechnology Journal*	Umbilical cord/ Wharton's jelly	Expansion/passages/senescence; Scale-up/3D/bioreactor; GMP/quality/release	10.1002/biot.202300381
Xu, Mingyuan (2024)	Isolation of Umbilical Cord-Derived Mesenchymal Stem Cells with High Yields and Low Damage	*JoVE: Journal of Visualized Experiments*	Umbilical cord/ Wharton's jelly	Isolation/tissue processing	10.3791/66835
Mahmoud R. (2024)	Maternal and neonatal factors' effects on wharton's jelly mesenchymal stem cell yield	*Scientific Reports*	Umbilical cord/ Wharton's jelly	Starting material/source	10.1038/s41598-024-72386-z
Kestendjieva, Snejana (2024)	Mesenchymal Stromal/Stem Cells Isolated by Explant Culture Method from Wharton's Jelly and Subamnion Possess Similar Biological Characteristics	*Applied Sciences-Basel*	Multiple perinatal sources	Starting material/source; Isolation/tissue processing	10.3390/app14178036
Soder, Rupal P. (2024)	Microcarrier-based clinical-grade manufacturing of therapeutic Wharton's jelly mesenchymal stromal cells	*Cytotherapy*	Umbilical cord/ Wharton's jelly	Scale-up/3D/bioreactor; GMP/quality/release	10.1016/j.jcyt.2024.07.003
Chu W.-L. (2024)	Stability study of umbilical cord	*Yaoxue Xuebao*	Umbilical cord/	Cryopreservation/storage; GMP/quality/release;	10.16438/j.0513-4870.2023-1063

	mesenchymal stem cells formulation in large-scale production		Wharton’s jelly	Potency/functional CQA	
Smolinska, Agnieszka (2024)	Stemness properties of SSEA-4+ subpopulation isolated from heterogenous Wharton’s jelly mesenchymal stem/stromal cells	*Frontiers in Cell and Developmental Biology*	Umbilical cord/ Wharton’s jelly	Isolation/tissue processing	10.3389/fcell.2024.1227034
Todtenhaupt, Pia (2023)	A robust and standardized method to isolate and expand mesenchymal stromal cells from human umbilical cord	*Cytotherapy*	Umbilical cord/ Wharton’s jelly	Isolation/tissue processing	10.1016/j.jcyt.2023.07.004
Koh, Benson (2023)	A Three-Dimensional Xeno-Free Culture Condition for Wharton's Jelly-Mesenchymal Stem Cells: The Pros and Cons	*International Journal of Molecular Sciences*	Umbilical cord/ Wharton’s jelly	Media/supplements; Scale-up/3D/bioreactor	10.3390/ijms24043745
Du, Shanshan (2023)	Carrageenan as a macromolecular crowding agent in human umbilical cord derived mesenchymal stromal cell culture	*International Journal of Biological Macromolecules*	Umbilical cord/ Wharton’s jelly	Media/supplements; Expansion/passages/senescence	10.1016/j.ijbiomac.2023.126353
Navakauskiene, Ruta (2023)	Effects of human placenta cryopreservation on molecular characteristics	*Frontiers in Bioengineering and Biotechnology*	Placenta/ amnion/ chorion/	Cryopreservation/storage	10.3389/fbioe.2023.1140781

	of placental mesenchymal stromal cells		decidu a		
Liu, Yujie (2023)	Large-scale expansion of human umbilical cord-derived mesenchymal stem cells using PLGA@PLL scaffold	*Bioresources and Bioprocessin g*	Umbili cal cord/ Whart on's jelly	Expansion/passages/se nescence; Scale-up/3D/bioreactor	10.1186/s40643-023-00635-6
Marino, Lincy Shiny (2023)	Lyophilized human platelet lysate as a supplementati on in the culture of umbilical cord derived mesenchymal stem cells	*Tissue & Cell*	Umbili cal cord/ Whart on's jelly	Media/supplements	10.1016/j.tice.2023.102092
Amend, Bastian (2023)	Regulation of Immune Checkpoint Antigen CD276 (B7-H3) on Human Placenta-Derived Mesenchymal Stromal Cells in GMP-Compliant Cell Culture Media	*International Journal of Molecular Sciences*	Placent a/ amnio n/ chorio n/ decidu a	Media/supplements; GMP/quality/release	10.3390/ijms242216422
Laroye, Caroline (2023)	Scale-Up of Academic Mesenchymal Stromal Cell Production	*Journal of Clinical Medicine*	Umbili cal cord/ Whart on's jelly	Scale-up/3D/bioreactor; GMP/quality/release	10.3390/jcm12134414
Padhiar C. (2023)	Sequential passage and characterizatio n of mesenchymal stem cells derived from single and pooled human	*Journal of Applied Biology and Biotechnolog y*	Umbili cal cord/ Whart on's jelly	Starting material/source; Expansion/passages/se nescence	10.7324/jabb.2023.11509

	umbilical cord tissue				
Kumar, Vikneswary Ravi (2023)	The Optimal Cryo Revival Period of Cryopreserved Wharton's Jelly Derived-Mesenchymal Stem Cells; [(Tempoh Pemulihan Krio Optimum bagi Sel Stem Mesenkima Wharton Jeli yang Diawet Krio)]	*Sains Malaysiana*	Umbilical cord/ Wharton's jelly	Cryopreservation/storage	10.17576/jsm-2023-5211-07
Abouelnaga, Hager (2022)	Characterization of mesenchymal stem cells isolated from Wharton's jelly of the human umbilical cord	*Egyptian Liver Journal*	Umbilical cord/ Wharton's jelly	Isolation/tissue processing	10.1186/s43066-021-00165-w
Siming C. (2022)	Comparison of biological characteristics of two kinds of primary isolated human umbilical cord mesenchymal stem cells under three-dimensional culture	*Chinese Journal of Tissue Engineering Research*	Umbilical cord/ Wharton's jelly	Isolation/tissue processing; Scale-up/3D/bioreactor	10.12307/2022.377
Dai, Yichen (2022)	Development of a novel feeding regime for large scale production of human umbilical cord mesenchymal stem/stromal cells	*Cytotechnology*	Umbilical cord/ Wharton's jelly	Scale-up/3D/bioreactor; GMP/quality/release	10.1007/s10616-022-00523-5

Zhang L. (2022)	Effects of hypoxia on proliferation and biological characteristics of human umbilical cord mesenchymal stem cells;	*Drug Evaluation Research*	Umbilical cord/ Wharton's jelly	Expansion/passages/senescence; Oxygen/process environment	10.7501/j.issn.1674-6376.2022.05.010
Padhiar, Chirayu (2022)	GMP compliant clinical grade and xenofree manufacturing of human Wharton?s jelly derived mesenchymal stem cell from pooled donors	*Biochemical Engineering Journal*	Umbilical cord/ Wharton's jelly	Starting material/source; Media/supplements; GMP/quality/release	10.1016/j.bej.2022.108470
Yang, Yanjun (2022)	Isolation and Culture of Three Kinds of Umbilical Cord Mesenchymal Stem Cells	*JoVE: Journal of Visualized Experiments*	Umbilical cord/ Wharton's jelly	Isolation/tissue processing	10.3791/64065
Zhang, Junhong (2022)	Large-Scale Expansion of Human Umbilical Cord-Derived Mesenchymal Stem Cells in a Stirred Suspension Bioreactor Enabled by Computational Fluid Dynamics Modeling	*Bioengineering-Basel*	Umbilical cord/ Wharton's jelly	Expansion/passages/senescence; Scale-up/3D/bioreactor	10.3390/bioengineering9070274
Wang, Juan (2022)	Manufacture and Quality Control of Human Umbilical Cord-Derived Mesenchymal Stem Cell Sheets for Clinical Use	*Cells*	Umbilical cord/ Wharton's jelly	GMP/quality/release	10.3390/cells11172732

Lee, Ah-Young (2022)	Minimal Cube Explant Provides Optimal Isolation Condition of Mesenchymal Stem Cells from Umbilical Cord	*Tissue Engineering and Regenerative Medicine*	Umbilical cord/ Wharton's jelly	Isolation/tissue processing	10.1007/s13770-022-00440-w
Chakraborty, Eliza (2022)	Optimizing aseptic and serum milieu for the isolation of human whole umbilical cord tissue-derived mesenchymal stem cells	*Beni-Suef University Journal of Basic and Applied Sciences*	Umbilical cord/ Wharton's jelly	Isolation/tissue processing; Media/supplements	10.1186/s43088-022-00308-x
Zhang, Mingqi (2022)	Study of the biological characteristics of human umbilical cord mesenchymal stem cells after long-time cryopreservation	*Cell and Tissue Banking*	Umbilical cord/ Wharton's jelly	Cryopreservation/storage	10.1007/s10561-021-09973-1
Kurogi, Hikari (2022)	Study on the Umbilical Cord-Mesenchymal Stem Cell Manufacturing Using Clinical-Grade Culture Medium	*Tissue Engineering Part C-Methods*	Umbilical cord/ Wharton's jelly	Media/supplements; Isolation/tissue processing; Expansion/passages/senescence; Scale-up/3D/bioreactor; GMP/quality/release	10.1089/ten.tec.2021.0207
Diego, Raffo (2021)	A protocol for umbilical cord tissue cryopreservation as a source of mesenchymal stem cells	*Molecular Biology Reports*	Umbilical cord/ Wharton's jelly	Cryopreservation/storage	10.1007/s11033-020-06079-x
Minh Quang Nguyen (2021)	Comparative Bioactivity Analysis for Off-the-Shelf	*Cell Transplantation*	Umbilical cord/ Whart	Media/supplements; Potency/functional CQA	10.1177/09636897211039441

	and Culture-Rescued Umbilical Cord-Derived Mesenchymal Stem/Stromal Cells in a Xeno- and Serum-Free Culture System		on’s jelly		
Peissert, Frederik (2021)	Expression of CD146 and Regenerative Cytokines by Human Placenta-Derived Mesenchymal Stromal Cells upon Expansion in Different GMP-Compliant Media	*Stem Cells International*	Placenta/ amnion/ chorion/ decidua	Media/supplements; Expansion/passages/senescence; GMP/quality/release; Potency/functional CQA	10.1155/2021/6662201
Noronha, Nadia C. (2021)	Hypoxia priming improves in vitro angiogenic properties of umbilical cord derived-mesenchymal stromal cells expanded in stirred-tank bioreactor	*Biochemical Engineering Journal*	Umbilical cord/ Wharton’s jelly	Oxygen/process environment; Scale-up/3D/bioreactor; Potency/functional CQA	10.1016/j.bej.2021.107949
Koltsova A.M. (2021)	Isolation and Characterization of Mesenchymal Stem Cells Derived from Different Regions of the Placenta of the Same Donor	*Cell and Tissue Biology*	Placenta/ amnion/ chorion/ decidua	Starting material/source; Isolation/tissue processing	10.1134/s1990519x21040040
Subramani, Priya (2021)	Isolation, Expansion, and Characterization of Placenta Originated	*ACS Omega*	Placenta/ amnion/ chorion/	Isolation/tissue processing; Expansion/passages/senescence	10.1021/acsomega.1c05022

	Decidua Basalis-Derived Mesenchymal Stromal Cells		decidua		
Teng, Sen-Wen (2021)	Potential surrogate quantitative immunomodulatory potency assay for monitoring human umbilical cord-derived mesenchymal stem cells production	*Cell Biology International*	Umbilical cord/ Wharton’s jelly	GMP/quality/release; Potency/functional CQA	10.1002/cbin.11553
Hoang, Van T. (2021)	Standardized xeno- and serum-free culture platform enables large-scale expansion of high-quality mesenchymal stem/stromal cells from perinatal and adult tissue sources	*Cytotherapy*	Umbilical cord/ Wharton’s jelly	Media/supplements; Expansion/passages/senescence; Scale-up/3D/bioreactor	10.1016/j.jcyt.2020.09.004
Mathen, Caroline (2021)	Xeno-Free Human Wharton's Jelly Mesenchymal Stromal Cells Maintain Their Characteristic Properties after Long-Term Cryopreservation	*Cell Journal*	Umbilical cord/ Wharton’s jelly	Media/supplements; Cryopreservation/storage	10.22074/cellj.2021.7131
Yi, Xiao (2020)	Comparative separation methods and biological characteristics of human placental and umbilical cord	*Stem Cell Research & Therapy*	Multiple perinatal sources	Starting material/source; Isolation/tissue processing; Media/supplements	10.1186/s13287-020-01690-y

	mesenchymal stem cells in serum-free culture conditions				
Miceli V. (2020)	Comparative study of the production of soluble factors in human placenta-derived mesenchymal stromal/stem cells grown in adherent conditions or as aggregates in a catheter-like device	*Biochemical and Biophysical Research Communications*	Placenta/ amnion/ chorion/ decidua	Scale-up/3D/bioreactor; Potency/functional CQA	10.1016/j.bbrc.2019.11.069
Skiles, Matthew L. (2020)	Comparison of umbilical cord tissue-derived mesenchymal stromal cells isolated from cryopreserved material and extracted by explantation and digestion methods utilizing a split manufacturing model	*Cytotherapy*	Umbilical cord/ Wharton's jelly	Isolation/tissue processing; Cryopreservation/storage; GMP/quality/release	10.1016/j.jcyt.2020.06.002
Lai, Baoyu (2020)	Cost-effective storage solution for delivering umbilical cord with efficient isolation of mesenchymal stem cells	*Biotechniques*	Umbilical cord/ Wharton's jelly	Isolation/tissue processing; Cryopreservation/storage	10.2144/btn-2019-0146
Wu, Xiaoyun (2020)	Derivation of clinical -grade mesenchymal stromal cells from umbilical cord under chemically defined culture	*Cytotherapy*	Umbilical cord/ Wharton's jelly	Media/supplements; Expansion/passages/senescence; GMP/quality/release	10.1016/j.jcyt.2020.03.431

	condition-platform for future clinical application				
Wang, Jianye (2020)	Effects of Recombinant AavLEA1 Protein on Human Umbilical Cord Matrix Mesenchymal Stem Cells Survival During Cryopreservation	*Biopreservation and Biobanking*	Umbilical cord/ Wharton's jelly	Cryopreservation/storage	10.1089/bio.2020.0014
Canas-Arboleda, Mariana (2020)	Human Platelet Lysate Supports Efficient Expansion and Stability of Wharton's Jelly Mesenchymal Stromal Cells via Active Uptake and Release of Soluble Regenerative Factors	*International Journal of Molecular Sciences*	Umbilical cord/ Wharton's jelly	Media/supplements; Expansion/passages/senescence; GMP/quality/release	10.3390/ijms21176284
Romanov, Yu. A. (2020)	Human Umbilical Cord Tissue-Derived Multipotent Mesenchymal Stromal Cells Exhibit Maximum Secretory Activity in the Presence of Umbilical Cord Blood Serum	*Bulletin of Experimental Biology and Medicine*	Umbilical cord/ Wharton's jelly	Media/supplements; Potency/functional CQA	10.1007/s10517-020-04926-2
Boss, Anna L. (2020)	Influence of culture media on the derivation and phenotype of fetal-derived placental	*Placenta*	Placenta/ amnion/ chorion/	Starting material/source; Media/supplements	10.1016/j.placenta.2020.09.002

	mesenchymal stem/stromal cells across gestation		decidu a		
Vymetalova, Ladislava (2020)	Large-Scale Automated Hollow-Fiber Bioreactor Expansion of Umbilical Cord-Derived Human Mesenchymal Stromal Cells for Neurological Disorders	*Neurochemic al Research*	Umbili cal cord/ Whart on's jelly	Expansion/passages/se nescence; Scale-up/3D/bioreactor	10.1007/s11064-019-02925-y
Park, Sang Eon (2020)	Pressure Stimuli Improve the Proliferation of Wharton's Jelly-Derived Mesenchymal Stem Cells under Hypoxic Culture Conditions	*International Journal of Molecular Sciences*	Umbili cal cord/ Whart on's jelly	Expansion/passages/se nescence; Oxygen/process environment	10.3390/ijms21197092
Liu, Yuncheng (2020)	Recovery and maintenance of NESTIN expression in umbilical cord-MSC using a novel culture medium	*AMB Express*	Umbili cal cord/ Whart on's jelly	Media/supplements	10.1186/s13568-020-01067-7
Kamalifar, Sulmaz (2020)	ROCK Y-27632 Inhibitor, Ascorbic Acid, and Trehalose Increase Survival of Human Wharton Jelly Mesenchymal Stem Cells After Cryopreservat ion	*Experimenta l and Clinical Transplantat ion*	Umbili cal cord/ Whart on's jelly	Cryopreservation/stor age	10.6002/ect.2017.0101

Pratama G. (2020)	Wharton's jelly derived mesenchymal stem cells: A comparison study in preterm vs. term deliveries and in FBS vs. PRP vs mesencult culture media	*Journal of Global Pharma Technology*	Umbilical cord/ Wharton's jelly	Starting material/source; Media/supplements	NR
Kang, Ji Yeon (2020)	Xeno-Free Condition Enhances Therapeutic Functions of Human Wharton's Jelly-Derived Mesenchymal Stem Cells against Experimental Colitis by Upregulated Indoleamine 2,3-Dioxygenase Activity	*Journal of Clinical Medicine*	Umbilical cord/ Wharton's jelly	Media/supplements; Oxygen/process environment	10.3390/jcm9092913
Mennan, Claire (2019)	A comprehensive characterisation of large-scale expanded human bone marrow and umbilical cord mesenchymal stem cells	*Stem Cell Research & Therapy*	Umbilical cord/ Wharton's jelly	Scale-up/3D/bioreactor	10.1186/s13287-019-1202-4
Wiese, Danielle M. (2019)	Accumulating Transcriptome Drift Precedes Cell Aging in Human Umbilical Cord-Derived Mesenchymal Stromal Cells Serially Cultured to Replicative Senescence	*Stem Cells Translational Medicine*	Umbilical cord/ Wharton's jelly	Expansion/passages/senescence	10.1002/sctm.18-0246

Raileanu, Vanessa N. (2019)	Banking Mesenchymal Stromal Cells from Umbilical Cord Tissue: Large Sample Size Analysis Reveals Consistency Between Donors	*Stem Cells Translational Medicine*	Umbilical cord/ Wharton's jelly	Starting material/source; Cryopreservation/storage	10.1002/sctm.19-0022
Ma, Jiao (2019)	Comparative analysis of mesenchymal stem cells derived from amniotic membrane, umbilical cord, and chorionic plate under serum-free condition	*Stem Cell Research & Therapy*	Multiple perinatal sources	Starting material/source; Media/supplements	10.1186/s13287-018-1104-x
Isildar, Basak (2019)	Comparison of different cryopreservation protocols for human umbilical cord tissue as source of mesenchymal stem cells	*Acta Histochemica*	Umbilical cord/ Wharton's jelly	Cryopreservation/storage	10.1016/j.acthis.2019.02.008
Miceli, Vitale (2019)	Comparison of Immunosuppressive and Angiogenic Properties of Human Amnion-Derived Mesenchymal Stem Cells between 2D and 3D Culture Systems	*Stem Cells International*	Placenta/ amnion/ chorion/ decidua	Scale-up/3D/bioreactor; Potency/functional CQA	10.1155/2019/7486279
Grau-Vorster, Marta (2019)	Compliance with Good Manufacturing Practice in the	*Cells*	Umbilical cord/ Whart	GMP/quality/release; Potency/functional CQA	10.3390/cells8050484

	Assessment of Immunomodulation Potential of Clinical Grade Multipotent Mesenchymal Stromal Cells Derived from Wharton's Jelly		on's jelly		
Jagiello, Joanna (2019)	Impact of Graphene-Based Surfaces on the Basic Biological Properties of Human Umbilical Cord Mesenchymal Stem Cells: Implications for Ex Vivo Cell Expansion Aimed at Tissue Repair	*International Journal of Molecular Sciences*	Umbilical cord/ Wharton's jelly	Expansion/passages/senescence	10.3390/ijms20184561
Loubiere, Celine (2019)	Impact of the type of microcarrier and agitation modes on the expansion performances of mesenchymal stem cells derived from umbilical cord	*Biotechnology Progress*	Umbilical cord/ Wharton's jelly	Expansion/passages/senescence; Scale-up/3D/bioreactor	10.1002/btpr.2887
Obradovic, Hristina (2019)	Improving stemness and functional features of mesenchymal stem cells from Wharton's jelly of a human umbilical cord by mimicking the native, low	*Placenta*	Umbilical cord/ Wharton's jelly	Oxygen/process environment; Potency/functional CQA	10.1016/j.placenta.2019.05.005

	oxygen stem cell niche				
Liu X. (2019)	Insulin-Transferrin-Selenium as a Novel Serum-free Media Supplement for the Culture of Human Amnion Mesenchymal Stem Cells	*Annals of Clinical and Laboratory Science*	Placenta/ amnion/ chorion/ decidua	Media/supplements	NR
Adani, Boaz (2019)	Isolation and expansion of high yield of pure mesenchymal stromal cells from fresh and cryopreserved placental tissues	*Cryobiology*	Placenta/ amnion/ chorion/ decidua	Isolation/tissue processing; Expansion/passages/senescence; Cryopreservation/storage	10.1016/j.cryobiol.2019.05.010
Mutsenko, Vitalii (2019)	Me2SO- and serum-free cryopreservation of human umbilical cord mesenchymal stem cells using electroporation-assisted delivery of sugars	*Cryobiology*	Umbilical cord/ Wharton's jelly	Media/supplements; Cryopreservation/storage	10.1016/j.cryobiol.2019.10.002
Laroye, Caroline (2019)	Mesenchymal Stem/Stromal Cell Production Compliant with Good Manufacturing Practice: Comparison between Bone Marrow, the Gold Standard Adult Source, and Wharton's Jelly, an Extraembryonic Source	*Journal of Clinical Medicine*	Umbilical cord/ Wharton's jelly	Starting material/source; GMP/quality/release	10.3390/jcm8122207

Celikkan, Ferda Topal (2019)	Optimizing the transport and storage conditions of current Good Manufacturing Practice - grade human umbilical cord mesenchymal stromal cells for transplantation (HUC-HEART Trial)	*Cytotherapy*	Umbilical cord/ Wharton's jelly	Cryopreservation/storage; GMP/quality/release	10.1016/j.jcyt.2018.10.010
Zhang F. (2018)	Biological characteristics of human placental mesenchymal stem cells isolated using two culture methods: A comparative study	*Chinese Journal of Tissue Engineering Research*	Placenta/ amnion/ chorion/ decidua	Isolation/tissue processing	10.3969/j.issn.2095-4344.0644
Taghizadeh, Rouzbeh R. (2018)	Collagenase Impacts the Quantity and Quality of Native Mesenchymal Stem/Stromal Cells Derived during Processing of Umbilical Cord Tissue	*Cell Transplantation*	Umbilical cord/ Wharton's jelly	Isolation/tissue processing	10.1177/0963689717744787
Mu, Yongxu (2018)	Comparative evaluation of mesenchymal stromal cells from umbilical cord and amniotic membrane in xeno-free conditions	*BMC Cell Biology*	Umbilical cord/ Wharton's jelly	Starting material/source; Media/supplements	10.1186/s12860-018-0178-8
Balgi-Agarwal, Saloni (2018)	Comparison of Preterm and Term Wharton's Jelly-Derived Mesenchymal Stem Cell	*Cells Tissues Organs*	Umbilical cord/ Wharton's jelly	Starting material/source; Oxygen/process environment	10.1159/000489256

	Properties in Different Oxygen Tensions				
Yan S.S. (2018)	Effect of cold storage time on cellular viability of umbilical cord mesenchymal stem cells	*Chinese Journal of Tissue Engineering Research*	Umbilical cord/ Wharton's jelly	Cryopreservation/storage	10.3969/j.issn.2095-4344.0446
Wang, Yiming (2018)	Extracellular matrix deposited by Wharton's jelly mesenchymal stem cells enhances cell expansion and tissue specific lineage potential	*American Journal of Translational Research*	Umbilical cord/ Wharton's jelly	Expansion/passages/senescence	NR
Hou P. (2018)	Feasibility of mixed human ab serum to expand human umbilical cord mesenchymal stem cells in vitro	*Chinese Journal of Tissue Engineering Research*	Umbilical cord/ Wharton's jelly	Media/supplements	10.3969/j.issn.2095-4344.0658
Sidharta V.M. (2018)	High VEGF level is produced by human umbilical cord-mesenchymal stem cells (hUC-MSCs) in amino acid-rich medium and under hypoxia condition	*Indonesian Biomedical Journal*	Umbilical cord/ Wharton's jelly	Media/supplements; Oxygen/process environment	10.18585/inabj.v10i3.457
Laflaquiere, Benoit (2018)	Identifying Biomarkers of Wharton's Jelly Mesenchymal Stromal Cells Using a Dynamic	*Metabolites*	Umbilical cord/ Wharton's jelly	Expansion/passages/senescence	10.3390/metabo8010018

	Metabolic Model: The Cell Passage Effect				
Abolhasani, Massume (2018)	Immunomodulatory properties of umbilical cord vein mesenchymal stromal cells influenced by gestational age and in vitro expansion	*Immunology Letters*	Umbilical cord/ Wharton's jelly	Starting material/source; Expansion/passages/senescence; Potency/functional CQA	10.1016/j.imlet.2017.11.008
Wang, Dan (2018)	Impact of Oxygen Concentration on Metabolic Profile of Human Placenta-Derived Mesenchymal Stem Cells As Determined by Chemical Isotope Labeling LC-MS	*Journal of Proteome Research*	Placenta/ amnion/ chorion/ decidua	Oxygen/process environment	10.1021/acs.jproteome.7b00887
Araujo, A. B. (2018)	Isolation of human mesenchymal stem cells from amnion, chorion, placental decidua and umbilical cord: comparison of four enzymatic protocols	*Biotechnology Letters*	Multiple perinatal sources	Isolation/tissue processing	10.1007/s10529-018-2546-z
Rizal R. (2018)	Isolation, characterization, proliferation, differentiation, and freeze–thaw survival of human wharton's jelly	*Bioscience Research*	Umbilical cord/ Wharton's jelly	Isolation/tissue processing; Expansion/passages/senescence; Cryopreservation/storage	NR

	mesenchymal stem cells from early and late passages				
Zhang, Shan (2018)	Mass-production of fluorescent chitosan/graphene oxide hybrid microspheres for in vitro 3D expansion of human umbilical cord mesenchymal stem cells	*Chemical Engineering Journal*	Umbilical cord/ Wharton's jelly	Expansion/passages/senescence; Scale-up/3D/bioreactor	10.1016/j.cej.2017.09.014
Ji-Nong Y. (2018)	Paracrine abilities of adipose-derived mesenchymal stem cells and umbilical cord mesenchymal stem cells under different oxygen concentrations	*Chinese Journal of Tissue Engineering Research*	Umbilical cord/ Wharton's jelly	Oxygen/process environment	10.3969/j.issn.2095-4344.0880
Zhou, Long (2018)	SIRT1-dependent anti-senescence effects of cell-deposited matrix on human umbilical cord mesenchymal stem cells	*Journal of Tissue Engineering and Regenerative Medicine*	Umbilical cord/ Wharton's jelly	Expansion/passages/senescence	10.1002/term.2422
Mizukami, Amanda (2018)	Technologies for large-scale umbilical cord-derived MSC expansion: Experimental performance and cost of goods analysis	*Biochemical Engineering Journal*	Umbilical cord/ Wharton's jelly	Expansion/passages/senescence; Scale-up/3D/bioreactor	10.1016/j.bej.2018.02.018

De Witte, Samantha F. H. (2017)	Aging of bone marrow- and umbilical cord-derived mesenchymal stromal cells during expansion	*Cytotherapy*	Umbilical cord/ Wharton's jelly	Expansion/passages/senescence	10.1016/j.jcyt.2017.03.071
Sardesai, Varda S. (2017)	Avoidance of Maternal Cell Contamination and Overgrowth in Isolating Fetal Chorionic Villi Mesenchymal Stem Cells from Human Term Placenta	*Stem Cells Translational Medicine*	Placenta/ amnion/ chorion/ decidua	Starting material/source; Isolation/tissue processing	10.1002/sctm.15-0327
Krishnanda S.I. (2017)	Comparison of various solutions for temporary storage of umbilical cord derived mesenchymal stem cells	*Annual Research and Review in Biology*	Umbilical cord/ Wharton's jelly	Cryopreservation/storage	10.9734/arrb/2017/38233
Kusuma, Gina D. (2017)	Decellularized extracellular matrices produced from immortal cell lines derived from different parts of the placenta support primary mesenchymal stem cell expansion	*PLOS ONE*	Placenta/ amnion/ chorion/ decidua	Starting material/source; Expansion/passages/senescence	10.1371/journal.pone.0171488
Choi Y.S. (2017)	Different characteristics of mesenchymal stem cells isolated from different layers of full term placenta	*PLOS ONE*	Placenta/ amnion/ chorion/ decidua	Starting material/source; Isolation/tissue processing	10.1371/journal.pone.0172642
Li, Li (2017)	Hypoxia modulates cell	*Journal of Thoracic and*	Placenta/	Expansion/passages/senescence;	10.1016/j.jtcvs.2017.03.141

	migration and proliferation in placenta-derived mesenchymal stem cells	*Cardiovascular Surgery*	amnion/ chorion/ decidua	Oxygen/process environment	
de Soure, Antonio M. (2017)	Integrated culture platform based on a human platelet lysate supplement for the isolation and scalable manufacturing of umbilical cord matrix-derived mesenchymal stem/stromal cells	*Journal of Tissue Engineering and Regenerative Medicine*	Umbilical cord/ Wharton's jelly	Isolation/tissue processing; Media/supplements; Scale-up/3D/bioreactor; GMP/quality/release	10.1002/term.2200
Beeravolu, Naimisha (2017)	Isolation and Characterization of Mesenchymal Stromal Cells from Human Umbilical Cord and Fetal Placenta	*JoVE: Journal of Visualized Experiments*	Multiple perinatal sources	Starting material/source; Isolation/tissue processing	10.3791/55224
Lankford, Lee (2017)	Manufacture and preparation of human placenta-derived mesenchymal stromal cells for local tissue delivery	*Cytotherapy*	Placenta/ amnion/ chorion/ decidua	Isolation/tissue processing; Expansion/passages/senescence; GMP/quality/release	10.1016/j.jcyt.2017.03.003
Barcia, Rita N. (2017)	Umbilical cord tissue-derived mesenchymal stromal cells maintain immunomodulatory and angiogenic potencies after cryopreservation and	*Cytotherapy*	Umbilical cord/ Wharton's jelly	Cryopreservation/storage; Potency/functional CQA	10.1016/j.jcyt.2016.11.008

	subsequent thawing				
Vellasamy S. (2016)	Basic fibroblast growth factor enhances the expansion and secretory profle of human placenta-derived mesenchymal stem cells	*Malaysian Journal of Medicine and Health Sciences*	Placenta/ amnion/ chorion/ decidua	Expansion/passages/senescence; Potency/functional CQA	NR
Paladino, Fernanda Vieira (2016)	Comparison between isolation protocols highlights intrinsic variability of human umbilical cord mesenchymal cells	*Cell and Tissue Banking*	Umbilical cord/ Wharton's jelly	Isolation/tissue processing	10.1007/s10561-015-9525-6
Yang, Yuanyuan (2016)	Conditioned umbilical cord tissue provides a natural three-dimensional storage compartment as in vitro stem cell niche for human mesenchymal stroma/stem cells	*Stem Cell Research & Therapy*	Umbilical cord/ Wharton's jelly	Starting material/source; Expansion/passages/senescence; Cryopreservation/storage	10.1186/s13287-016-0289-0
Liu F. (2016)	Different culture methods for human umbilical cord mesenchymal stem cells	*Chinese Journal of Tissue Engineering Research*	Umbilical cord/ Wharton's jelly	Isolation/tissue processing	10.3969/j.issn.2095-4344.2016.28.005
Emnett, Ryan J. (2016)	Evaluation of Tissue Homogenization to Support the Generation of GMP-Compliant	*Stem Cells International*	Umbilical cord/ Wharton's jelly	Isolation/tissue processing; Media/supplements; Expansion/passages/senescence; GMP/quality/release	10.1155/2016/3274054

	Mesenchymal Stromal Cells from the Umbilical Cord				
Fong, Chui-Yee (2016)	Freezing of Fresh Wharton's Jelly From Human Umbilical Cords Yields High Post-Thaw Mesenchymal Stem Cell Numbers for Cell-Based Therapies	*Journal of Cellular Biochemistry*	Umbilical cord/ Wharton's jelly	Cryopreservation/storage	10.1002/jcb.25375
Ji, Mingli (2016)	Immunogenicity of human umbilical cord mesenchymal stem cells during ex vivo expansion	*Indian Journal of Animal Research*	Umbilical cord/ Wharton's jelly	Expansion/passages/senescence; Potency/functional CQA	10.18805/ijar.9541
Pelekanos, Rebecca A. (2016)	Isolation and Expansion of Mesenchymal Stem/Stromal Cells Derived from Human Placenta Tissue	*JoVE: Journal of Visualized Experiments*	Placenta/ amnion/ chorion/ decidua	Isolation/tissue processing; Expansion/passages/senescence	10.3791/54204
Petry, Florian (2016)	Manufacturing of Human Umbilical Cord Mesenchymal Stromal Cells on Microcarriers in a Dynamic System for Clinical Use	*Stem Cells International*	Umbilical cord/ Wharton's jelly	Expansion/passages/senescence; Scale-up/3D/bioreactor; GMP/quality/release	10.1155/2016/4834616
Shuvalova N. (2016)	Morphological characteristics of mesenchymal stem cells from wharton jelly, cultuivated under	*Biopolymers and Cell*	Umbilical cord/ Wharton's jelly	Oxygen/process environment	10.7124/bc.000928

	physiological oxygen tensions, in various gas mixtures				
Lim J. (2016)	MSCs can be differentially isolated from maternal, middle and fetal segments of the human umbilical cord	*Cytotherapy*	Umbilical cord/ Wharton's jelly	Starting material/source; Isolation/tissue processing	10.1016/j.jcyt.2016.08.003
Wu, Xiaoyun (2016)	Serum and xeno-free, chemically defined, no-plate-coating-based culture system for mesenchymal stromal cells from the umbilical cord	*Cell Proliferation*	Umbilical cord/ Wharton's jelly	Media/supplements	10.1111/cpr.12279
Smith, J. Robert (2016)	Standardizing Umbilical Cord Mesenchymal Stromal Cells for Translation to Clinical Use: Selection of GMP-Compliant Medium and a Simplified Isolation Method	*Stem Cells International*	Umbilical cord/ Wharton's jelly	Isolation/tissue processing; Media/supplements; Expansion/passages/senescence; GMP/quality/release	10.1155/2016/6810980
Mizukami, Amanda (2016)	Stirred tank bioreactor culture combined with serum-/xenogeneic-free culture medium enables an efficient expansion of umbilical cord-derived mesenchymal stem/stromal cells	*Biotechnology Journal*	Umbilical cord/ Wharton's jelly	Media/supplements; Expansion/passages/senescence; Scale-up/3D/bioreactor	10.1002/biot.201500532

Esmaeli A. (2016)	Xeno-free culture condition for human bone marrow and umbilical cord matrix-derived mesenchymal stem/stromal cells using human umbilical cord blood serum	*International Journal of Reproductive BioMedicine*	Umbilical cord/ Wharton's jelly	Media/supplements	10.29252/ijrm.14.9.567
Li, Shengying (2015)	Characteristics of human umbilical cord mesenchymal stem cells during ex vivo expansion	*Molecular Medicine Reports*	Umbilical cord/ Wharton's jelly	Expansion/passages/senescence; Potency/functional CQA	10.3892/mmr.2015.3999
Subramanian, Arjunan (2015)	Comparative Characterization of Cells from the Various Compartments of the Human Umbilical Cord Shows that the Wharton's Jelly Compartment Provides the Best Source of Clinically Utilizable Mesenchymal Stem Cells	*PLOS ONE*	Umbilical cord/ Wharton's jelly	Starting material/source	10.1371/journal.pone.0127992
Shuvalova N.S. (2015)	Comparison of proliferative activity of Wharton jelly mesenchymal stem cells in cultures under various gas conditions	*Biopolymers and Cell*	Umbilical cord/ Wharton's jelly	Oxygen/process environment; Expansion/passages/senescence	10.7124/bc.0008e5
Shivakumar, Sharath Belame (2015)	Cryopreservation of Human Wharton's Jelly-derived	*International Journal of Stem Cells*	Umbilical cord/ Whart	Cryopreservation/storage	10.15283/ijsc.2015.8.2.155

	Mesenchymal Stem Cells Following Controlled Rate Freezing Protocol Using Different Cryoprotectants; A Comparative Study		on's jelly		
Du L.-L. (2015)	Effect of different oxygen concentration on the biological characteristics of human placenta derived mesenchymal stem cells	*Journal of Dalian Medical University*	Placenta/ amnion/ chorion/ decidua	Oxygen/process environment	10.11724/jdmu.2015.05.07
Park, Saeyoung (2015)	Establishment of a xeno-free culture system that preserves the characteristics of placenta mesenchymal stem cells	*Cytotechnology*	Placenta/ amnion/ chorion/ decidua	Media/supplements; Expansion/passages/senescence; Potency/functional CQA	10.1007/s10616-014-9725-0
Antoninus A.A. (2015)	Human platelet lysate enhances the proliferation of Wharton's jelly-derived mesenchymal stem cells	*Biomarkers and Genomic Medicine*	Umbilical cord/ Wharton's jelly	Media/supplements; Expansion/passages/senescence	10.1016/j.bgm.2015.06.001
Mori, Yuka (2015)	Improved Explant Method to Isolate Umbilical Cord-Derived Mesenchymal Stem Cells and Their Immunosuppressive Properties	*Tissue Engineering Part C-Methods*	Umbilical cord/ Wharton's jelly	Isolation/tissue processing; Potency/functional CQA	10.1089/ten.tec.2014.0385

Gao Y.-L. (2015)	Improved primary culture system increases the yield of human umbilical cord mesenchymal stem cells	*Chinese Journal of Tissue Engineering Research*	Umbilical cord/ Wharton's jelly	Isolation/tissue processing	10.3969/j.issn.2095-4344.2015.10.001
Secunda, R. (2015)	Isolation, expansion and characterisation of mesenchymal stem cells from human bone marrow, adipose tissue, umbilical cord blood and matrix: a comparative study	*Cytotechnology*	Umbilical cord/ Wharton's jelly	Starting material/source; Isolation/tissue processing; Expansion/passages/senescence	10.1007/s10616-014-9718-z
Romanov, Yu. A. (2015)	Optimized Protocol for Isolation of Multipotent Mesenchymal Stromal Cells from Human Umbilical Cord	*Bulletin of Experimental Biology and Medicine*	Umbilical cord/ Wharton's jelly	Isolation/tissue processing	10.1007/s10517-015-3116-1
Mediana D. (2015)	Passage effect on aging of human umbilical cord derived mesenchymal stem Cell	*OnLine Journal of Biological Sciences*	Umbilical cord/ Wharton's jelly	Expansion/passages/senescence	10.3844/ojbsci.2015.170.177
Amiri, Fatemeh (2015)	Positive selection of Wharton's jelly-derived CD105+ cells by MACS technique and their subsequent cultivation under suspension culture condition: A simple, versatile	*Hematology*	Umbilical cord/ Wharton's jelly	Isolation/tissue processing; Expansion/passages/senescence; Scale-up/3D/bioreactor; Potency/functional CQA	10.1179/1607845414y.0000000185

	culturing method to enhance the multipotentiality of mesenchymal stem cells				
Shimazu, Takahisa (2015)	Serum- and xeno-free cryopreservation of human umbilical cord tissue as mesenchymal stromal cell source	*Cytotherapy*	Umbilical cord/ Wharton's jelly	Media/supplements; Cryopreservation/storage	10.1016/j.jcyt.2015.03.604
Li, Yi (2015)	Three-dimensional spheroid culture of human umbilical cord mesenchymal stem cells promotes cell yield and stemness maintenance	*Cell and Tissue Research*	Umbilical cord/ Wharton's jelly	Scale-up/3D/bioreactor	10.1007/s00441-014-2055-x
Budiyanti E. (2015)	Umbilical cord derived mesenchymal stem cell proliferation in various platelet rich plasma and xeno-material containing medium	*International Journal of Research in Pharmaceutical Sciences*	Umbilical cord/ Wharton's jelly	Media/supplements; Expansion/passages/senescence	NR
Roy, Saheli (2014)	A simple and serum-free protocol for cryopreservation of human umbilical cord as source of Wharton's jelly mesenchymal stem cells	*Cryobiology*	Umbilical cord/ Wharton's jelly	Media/supplements; Cryopreservation/storage	10.1016/j.cryobiol.2014.03.010
Julavijitphong, Suphakde (2014)	A xeno-free culture method that enhances	*Cytotherapy*	Umbilical cord/ Whart	Media/supplements; Expansion/passages/senescence;	10.1016/j.jcyt.2013.07.012

	Wharton's jelly mesenchymal stromal cell culture efficiency over traditional animal serum supplemented cultures		on's jelly	Cryopreservation/storage	
Swamynathan, Priyanka (2014)	Are serum-free and xeno-free culture conditions ideal for large scale clinical grade expansion of Wharton's jelly derived mesenchymal stem cells? A comparative study	*Stem Cell Research & Therapy*	Umbilical cord/ Wharton's jelly	Media/supplements; Expansion/passages/senescence; GMP/quality/release	10.1186/scrt477
Cheng M. (2014)	Biological characters and genetic stability of umbilical cord mesenchymal stem cells after long-term culture in vitro	*Chinese Journal of Biologicals*	Umbilical cord/ Wharton's jelly	Expansion/passages/senescence; GMP/quality/release	NR
Hua, Jie (2014)	Comparison of different methods for the isolation of mesenchymal stem cells from umbilical cord matrix: Proliferation and multilineage differentiation as compared to mesenchymal stem cells from umbilical cord blood and bone marrow	*Cell Biology International*	Umbilical cord/ Wharton's jelly	Isolation/tissue processing; Expansion/passages/senescence	10.1002/cbin.10188

Hendijani, Fatemeh (2014)	Comparison of human mesenchymal stem cells isolated by explant culture method from entire umbilical cord and Wharton's jelly matrix	*Cell and Tissue Banking*	Umbilical cord/ Wharton's jelly	Starting material/source; Isolation/tissue processing; Expansion/passages/senescence	10.1007/s10561-014-9425-1
Wang F. (2014)	Culture methods for primary human umbilical cord mesenchymal stem cells	*Chinese Journal of Tissue Engineering Research*	Umbilical cord/ Wharton's jelly	Isolation/tissue processing	10.3969/j.issn.2095-4344.2014.19.015
Widowati W. (2014)	Effect of oxygen tension on proliferation and characteristics of Wharton's jelly-derived mesenchymal stem cells	*Biomarkers and Genomic Medicine*	Umbilical cord/ Wharton's jelly	Expansion/passages/senescence; Oxygen/process environment	10.1016/j.bgm.2014.02.001
Wan Y. (2014)	Effects of Corning® CellBIND® Surface medium on growth of human umbilical cord mesenchymal stem cells	*Chinese Journal of Tissue Engineering Research*	Umbilical cord/ Wharton's jelly	Media/supplements	10.3969/j.issn.2095-4344.2014.10.011
Li F. (2014)	Effects of three-dimensional culture on cell phenotype and hematopoietic cytokine expression of umbilical cord mesenchymal stem cells	Zhongguo shi yan xue ye xue za zhi/Zhongguo bing li sheng li xue hui = *Journal of Experimental Hematology*/ Chinese Association of Pathophysiology	Umbilical cord/ Wharton's jelly	Scale-up/3D/bioreactor; Potency/functional CQA	10.7534/j.issn.1009-2137.2014.05.043

Chen, Gecai (2014)	Human Umbilical Cord-Derived Mesenchymal Stem Cells Do Not Undergo Malignant Transformation during Long-Term Culturing in Serum-Free Medium	*PLOS ONE*	Umbilical cord/ Wharton's jelly	Media/supplements; Expansion/passages/senescence; GMP/quality/release	10.1371/journal.pone.0098565
Reppel, Loic (2014)	Hypoxic Culture Conditions for Mesenchymal Stromal/Stem Cells from Wharton's Jelly: A Critical Parameter to Consider in a Therapeutic Context	*Current Stem Cell Research & Therapy*	Umbilical cord/ Wharton's jelly	Oxygen/process environment	10.2174/1574888x09666140213204850
Ruan, Zhong-Bao (2014)	Karyotype stability of human umbilical cord-derived mesenchymal stem cells during in vitro culture	*Experimental and Therapeutic Medicine*	Umbilical cord/ Wharton's jelly	GMP/quality/release	10.3892/etm.2014.1977
Drela, Katarzyna (2014)	Low oxygen atmosphere facilitates proliferation and maintains undifferentiated state of umbilical cord mesenchymal stem cells in an hypoxia inducible factor-dependent manner	*Cytotherapy*	Umbilical cord/ Wharton's jelly	Expansion/passages/senescence; Oxygen/process environment	10.1016/j.jcyt.2014.02.009
Ilic, Nina (2014)	Manufacturing and use of human placenta-derived	*Vojnosanitetski Pregled*	Placenta/ amnion/ chorio	GMP/quality/release; Isolation/tissue processing; Expansion/passages/senescence	10.2298/vsp130410050i

	mesenchymal stromal cells for phase i clinical trials: Establishment and evaluation of a protocol; [Proizvodnja i upotreba humanih mezenhimskih stromalnih čelija izolovanih iz placentě za klinická istraživanja prve faze: Uspostavljanje i procena protokola]		n/ decidu a		
Chen, Gecai (2014)	Monitoring the biology stability of human umbilical cord-derived mesenchymal stem cells during long-term culture in serum-free medium	*Cell and Tissue Banking*	Umbili cal cord/ Whart on's jelly	Media/supplements; Expansion/passages/se nescence; GMP/quality/release	10.1007/s10561-014-9420-6
Chatzistama tiou, Theofanis K. (2014)	Optimizing isolation culture and freezing methods to preserve Wharton's jelly's mesenchymal stem cell (MSC) properties: an MSC banking protocol validation for the Hellenic Cord Blood Bank	*Transfusion*	Umbili cal cord/ Whart on's jelly	Isolation/tissue processing; Cryopreservation/stor age	10.1111/trf.12743
Chang, Zhengqi (2014)	Umbilical Cord Wharton's	*PLOS ONE*	Umbili cal cord/	Isolation/tissue processing;	10.1371/journal.pone.011076 4

	Jelly Repeated Culture System: A New Device and Method for Obtaining Abundant Mesenchymal Stem Cells for Bone Tissue Engineering		Wharton's jelly	Expansion/passages/senescence	
Devito, Liani (2014)	Wharton's jelly mesenchymal stromal/stem cells derived under chemically defined animal product-free low oxygen conditions are rich in MSCA-1+ subpopulation	*Regenerative Medicine*	Umbilical cord/ Wharton's jelly	Media/supplements; Oxygen/process environment	10.2217/rme.14.60
Zhou P. (2013)	[Serum-free culture of umbilical cord mesenchymal stem cells].	Zhongguo shi yan xue ye xue za zhi/Zhongguo bing li sheng li xue hui = *Journal of Experimental Hematology*/ Chinese Association of Pathophysiology	Umbilical cord/ Wharton's jelly	Media/supplements; Expansion/passages/senescence	10.7534/j.issn.1009-2137.2013.05.034
Kim, Sun-Mi (2013)	Alternative Xeno-Free Biomaterials Derived from Human Umbilical Cord for the Self-Renewal Ex vivo Expansion of Mesenchymal Stem Cells	*Stem Cells and Development*	Umbilical cord/ Wharton's jelly	Media/supplements; Expansion/passages/senescence	10.1089/scd.2013.0067

Loenne, Maren (2013)	Analysis of oxygen-dependent cytokine expression in human mesenchymal stem cells derived from umbilical cord	*Cell and Tissue Research*	Umbilical cord/ Wharton's jelly	Oxygen/process environment; Potency/functional CQA	10.1007/s00441-013-1597-7
Christodoulou, I. (2013)	Comparative Evaluation of Human Mesenchymal Stem Cells of Fetal (Wharton's Jelly) and Adult (Adipose Tissue) Origin during Prolonged In Vitro Expansion: Considerations for Cytotherapy	*Stem Cells International*	Umbilical cord/ Wharton's jelly	Starting material/source; Expansion/passages/senescence; Potency/functional CQA	10.1155/2013/246134
Liu L.-Y. (2013)	Comparison of different methods for the isolation of human umbilical cord mesenchymal stem cells	*National Medical Journal of China*	Umbilical cord/ Wharton's jelly	Isolation/tissue processing	10.3760/cma.j.issn.0376-2491.2013.32.016
Hao, Haojie (2013)	Culturing on Wharton's Jelly Extract Delays Mesenchymal Stem Cell Senescence through p53 and p16INK4a/pRb Pathways	*PLOS ONE*	Umbilical cord/ Wharton's jelly	Expansion/passages/senescence	10.1371/journal.pone.0058314
Chen, Yan (2013)	Effects of Storage Solutions on the Viability of Human Umbilical Cord Mesenchymal	*Cell Transplantation*	Umbilical cord/ Wharton's jelly	Cryopreservation/storage	10.3727/096368912x657602

	Stem Cells for Transplantation				
Roselli, Emanuela Anna (2013)	Fetal mesenchymal stromal cells from cryopreserved human chorionic villi: cytogenetic and molecular analysis of genome stability in long-term cultures	*Cytotherapy*	Placenta/ amnion/ chorion/ decidua	Starting material/source; Expansion/passages/senescence; Cryopreservation/storage; GMP/quality/release	10.1016/j.jcyt.2013.06.019
Borghesi, Alessandro (2013)	Genomic alterations in human umbilical cord-derived mesenchymal stromal cells call for stringent quality control before any possible therapeutic approach	*Cytotherapy*	Umbilical cord/ Wharton's jelly	GMP/quality/release	10.1016/j.jcyt.2013.06.006
Mennan, Claire (2013)	Isolation and Characterisation of Mesenchymal Stem Cells from Different Regions of the Human Umbilical Cord	*Biomed Research International*	Umbilical cord/ Wharton's jelly	Starting material/source; Isolation/tissue processing	10.1155/2013/916136
Corotchi, Maria Cristina (2013)	Isolation method and xeno-free culture conditions influence multipotent differentiation capacity of human Wharton's jelly-derived mesenchymal stem cells	*Stem Cell Research & Therapy*	Umbilical cord/ Wharton's jelly	Isolation/tissue processing; Media/supplements; Expansion/passages/senescence	10.1186/scrt232

Pu S.-B. (2013)	Large-scale multiplication of human umbilical cord mesenchymal stem cells in platelet lysate	*Chinese Journal of Biologicals*	Umbilical cord/ Wharton's jelly	Media/supplements; Scale-up/3D/bioreactor	NR
Yang Z.-X. (2013)	Long term in vitro expansion reduces immune modulation function of placental chorionic villi mesenchymal stem cells	Zhongguo shi yan xue ye xue za zhi / Zhongguo bing li sheng li xue hui = *Journal of Experimental Hematology*/ Chinese Association of Pathophysiology	Placenta/ amnion/ chorion/ decidua	Expansion/passages/senescence	10.7534/j.issn.1009-2137.2013.06.035
Iftimia-Mander, Andreea (2013)	Mesenchymal Stem Cell Isolation from Human Umbilical Cord Tissue: Understanding and Minimizing Variability in Cell Yield for Process Optimization	*Biopreservation and Biobanking*	Umbilical cord/ Wharton's jelly	Isolation/tissue processing	10.1089/bio.2013.0027
Han, Yan-Fu (2013)	Optimization of human umbilical cord mesenchymal stem cell isolation and culture methods	*Cytotechnology*	Umbilical cord/ Wharton's jelly	Isolation/tissue processing	10.1007/s10616-012-9528-0
Niu Y.-H. (2013)	Oxidative stress effect on viability of umbilical cord-derived mesenchymal stem cells in storage solution of	*Chinese Journal of Tissue Engineering Research*	Umbilical cord/ Wharton's jelly	Cryopreservation/storage	10.3969/j.issn.2095-4344.2013.32.007

	transplantation				
Zhou T.-T. (2013)	Primary culture of umbilical cord-derived mesenchymal stem cells in serum free medium	*Chinese Journal of Tissue Engineering Research*	Umbilical cord/ Wharton's jelly	Media/supplements	10.3969/j.issn.2095-4344.2013.27.006
Zhang S.-X. (2013)	Semi-continuous perfusion culture process of human umbilical cord-derived mesenchymal stem cells: Initial time, perfusion frequency and perfusion volume	*Chinese Journal of Tissue Engineering Research*	Umbilical cord/ Wharton's jelly	Scale-up/3D/bioreactor	10.3969/j.issn.2095-4344.2013.06.010
Balci, Deniz (2013)	The Assessment of Cryopreservation Conditions for Human Umbilical Cord Stroma-Derived Mesenchymal Stem Cells towards a Potential Use for Stem Cell Banking	*Current Stem Cell Research & Therapy*	Umbilical cord/ Wharton's jelly	Cryopreservation/storage	10.2174/1574888x11308010008
Gong, Wei (2012)	Banking Human Umbilical Cord-Derived Mesenchymal Stromal Cells for Clinical Use	*Cell Transplantation*	Umbilical cord/ Wharton's jelly	Cryopreservation/storage	10.3727/096368911x586756
Wang, Libin (2012)	Characterization of placenta-derived mesenchymal stem cells	*Molecular Medicine Reports*	Placenta/ amnion/ chorion/	Media/supplements	10.3892/mmr.2012.1000

	cultured in autologous human cord blood serum		decidu a		
Timmins, N. E. (2012)	Closed system isolation and scalable expansion of human placental mesenchymal stem cells	*Biotechnolog y and Bioengineeri ng*	Placent a/ amnio n/ chorio n/ decidu a	Isolation/tissue processing; Expansion/passages/se nescence	10.1002/bit.24425
Salehinejad, Parvin (2012)	Comparison of different methods for the isolation of mesenchymal stem cells from human umbilical cord Wharton's jelly	*In Vitro Cellular & Development al Biology-Animal*	Umbili cal cord/ Whart on's jelly	Isolation/tissue processing	10.1007/s11626-011-9480-x
Prata, Karen de Lima (2012)	Cryopreservat ion of umbilical cord mesenchymal cells in xenofree conditions	*Cytotherapy*	Umbili cal cord/ Whart on's jelly	Media/supplements; Cryopreservation/stor age	10.3109/14653249.2012.67782 0
Ha J.-W. (2012)	Do the fibroblasts contained in early passage MSC population adversely affect the characteristics of stem cell population obtained from human placenta?	*International Journal of Stem Cells*	Placent a/ amnio n/ chorio n/ decidu a	Expansion/passages/se nescence	10.15283/ijsc.2012.5.2.89
Salehinejad, Parvin (2012)	Effect of culture media on expansion properties of human umbilical cord matrix-derived mesenchymal cells	*Cytotherapy*	Umbili cal cord/ Whart on's jelly	Media/supplements; Expansion/passages/se nescence	10.3109/14653249.2012.68437 7

Poloni, A. (2012)	Human AB serum for generation of mesenchymal stem cells from human chorionic villi: comparison with other source and other media including platelet lysate	*Cell Proliferation*	Placenta/ amnion/ chorion/ decidua	Media/supplements	10.1111/j.1365-2184.2011.00799.x
Zhang, Hao (2012)	Isolation and characterization of mesenchymal stem cells from whole human umbilical cord applying a single enzyme approach	*Cell Biochemistry and Function*	Umbilical cord/ Wharton's jelly	Isolation/tissue processing	10.1002/cbf.2843
Sabapathy, Vikram (2012)	Long-Term Cultured Human Term Placenta-Derived Mesenchymal Stem Cells of Maternal Origin Displays Plasticity	*Stem Cells International*	Placenta/ amnion/ chorion/ decidua	Starting material/source; Expansion/passages/senescence	10.1155/2012/174328
Margossian, Talar (2012)	Mesenchymal stem cells derived from Wharton's jelly: Comparative phenotype analysis between tissue and in vitro expansion	*Bio-Medical Materials and Engineering*	Umbilical cord/ Wharton's jelly	Expansion/passages/senescence	10.3233/bme-2012-0714
De Bruyn, Cecile (2011)	A Rapid, Simple, and Reproducible Method for the Isolation of Mesenchymal Stromal Cells from	*Stem Cells and Development*	Umbilical cord/ Wharton's jelly	Isolation/tissue processing	10.1089/scd.2010.0260

	Wharton's Jelly Without Enzymatic Treatment				
López Y. (2011)	Evaluating the impact of oxygen concentration and plating density on human wharton's jelly-derived mesenchymal stromal cells	*Open Tissue Engineering and Regenerative Medicine Journal*	Umbilical cord/ Wharton's jelly	Oxygen/process environment	10.2174/1875043501104010082
Zhou Q. (2011)	Expansion and osteogenic differentiation of human amnion mesenchymal stem cell in a microcarrier-based dynamic culture system	Gao Xiao Hua Xue Gong Cheng Xue Bao/*Journal of Chemical Engineering of Chinese Universities*	Placenta/ amnion/ chorion/ decidua	Expansion/passages/senescence; Scale-up/3D/bioreactor	NR
Poloni, Antonella (2011)	Human Mesenchymal Stem Cells From Chorionic Villi and Amniotic Fluid Are Not Susceptible to Transformation After Extensive In Vitro Expansion	*Cell Transplantation*	Placenta/ amnion/ chorion/ decidua	Expansion/passages/senescence	10.3727/096368910x536518
Tsagias, N. (2011)	Isolation of mesenchymal stem cells using the total length of umbilical cord for transplantation purposes	*Transfusion Medicine*	Umbilical cord/ Wharton's jelly	Isolation/tissue processing	10.1111/j.1365-3148.2011.01076.x
Capelli, Chiara (2011)	Minimally manipulated whole human umbilical cord is a rich	*Cytotherapy*	Umbilical cord/ Whart	Media/supplements; GMP/quality/release	10.3109/14653249.2011.563294

	source of clinical-grade human mesenchymal stromal cells expanded in human platelet lysate		on's jelly		
Cooper K. (2010)	Derivation, expansion and characterization of clinical grade mesenchymal stem cells from umbilical cord matrix using cord blood serum	*International Journal of Stem Cells*	Umbilical cord/ Wharton's jelly	Media/supplements; Expansion/passages/senescence; GMP/quality/release	10.15283/ijsc.2010.3.2.119
Ba Y.-T. (2010)	Effects of basic fibroblast growth factor in culture of mesenchymal stem cells derived from Wharton's jelly of human umbilical cord	*Journal of Clinical Rehabilitative Tissue Engineering Research*	Umbilical cord/ Wharton's jelly	Media/supplements; Expansion/passages/senescence	10.3969/j.issn.1673-8225.2010.19.019
Lavrentieva, Antonina (2010)	Effects of hypoxic culture conditions on umbilical cord-derived human mesenchymal stem cells	*Cell Communication and Signaling*	Umbilical cord/ Wharton's jelly	Oxygen/process environment	10.1186/1478-811x-8-18
Nekanti, Usha (2010)	Long-Term Expansion and Pluripotent Marker Array Analysis of Wharton's Jelly-Derived Mesenchymal Stem Cells	*Stem Cells and Development*	Umbilical cord/ Wharton's jelly	Expansion/passages/senescence	10.1089/scd.2009.0177
Lu Y. (2009)	Comparison of four digestion methods in isolating mesenchymal	*Journal of Clinical Rehabilitative Tissue Engineering Research*	Placenta/ amnion/ chorion/	Isolation/tissue processing	NR

	stem cells from human placenta		decidua		
Wang J. (2009)	Effects of hypoxia on proliferation of hBMSCs and human placental decidua basalis-MSCs	Zhongguo xiu fu chong jian wai ke za zhi = Zhongguo xiufu chongjian waike zazhi = *Chinese Journal of Reparative and Reconstructive Surgery*	Placenta/ amnion/ chorion/ decidua	Expansion/passages/senescence; Oxygen/process environment	NR
Girdlestone, John (2009)	Efficient expansion of mesenchymal stromal cells from umbilical cord under low serum conditions	*Tissue Antigens*	Umbilical cord/ Wharton's jelly	Media/supplements; Expansion/passages/senescence	10.3109/14653240903079401
Yu, Yanqiu (2009)	Ex Vitro Expansion of Human Placenta-Derived Mesenchymal Stem Cells in Stirred Bioreactor	*Applied Biochemistry and Biotechnology*	Placenta/ amnion/ chorion/ decidua	Expansion/passages/senescence; Scale-up/3D/bioreactor	10.1007/s12010-009-8556-5
Yao D.-D. (2009)	Growth of human placenta-derived mesenchymal stem cells cultured on different modified polystyrene surfaces	*Journal of Clinical Rehabilitative Tissue Engineering Research*	Placenta/ amnion/ chorion/ decidua	Expansion/passages/senescence	10.3969/j.issn.1673-8225.2009.25.014
Schugar, Rebecca C. (2009)	High Harvest Yield, High Expansion, and Phenotype Stability of CD146 Mesenchymal	*Journal of Biomedicine and Biotechnology*	Umbilical cord/ Wharton's jelly	Expansion/passages/senescence	10.1155/2009/789526

	Stromal Cells from Whole Primitive Human Umbilical Cord Tissue				
Reinisch A. (2009)	Isolation and animal serum free expansion of human umbilical cord derived mesenchymal stromal cells (MSCs) and endothelial colony forming progenitor cells (ECFCs)	*Journal of Visualized Experiments*	Umbilical cord/ Wharton's jelly	Isolation/tissue processing; Media/supplements; Expansion/passages/senescence	10.3791/1525
Yu Y.-Q. (2009)	Large scale expansion of human placenta-derived mesenchymal cells in stirring bioreactor	*Chinese Journal of Biomedical Engineering*	Placenta/ amnion/ chorion/ decidua	Expansion/passages/senescence; Scale-up/3D/bioreactor	NR
Brooke, Gary (2009)	Manufacturing of human placenta-derived mesenchymal stem cells for clinical trials	*British Journal of Haematology*	Placenta/ amnion/ chorion/ decidua	GMP/quality/release	10.1111/j.1365-2141.2008.07492.x
Xu Y. (2009)	Optimization of culture condition and biological characteristics of human umbilical cord-derived mesenchymal stem cells	*Journal of Clinical Rehabilitative Tissue Engineering Research*	Umbilical cord/ Wharton's jelly	Media/supplements; Expansion/passages/senescence	10.3969/j.issn.1673-8225.2009.32.017
Kadam, Sachin S. (2009)	Simultaneous isolation of vascular endothelial cells and mesenchymal stem cells	*In Vitro Cellular & Developmental Biology-Animal*	Umbilical cord/ Wharton's jelly	Isolation/tissue processing	10.1007/s11626-008-9155-4

	from the human umbilical cord				
Huang Y.-C. (2008)	Effects of hypoxia on human placental decidua basalis-mesenchymal stem cells proliferation, apoptosis and VEGF expression	*Sheng li xue bao: [Acta physiologica Sinica]*	Placenta/ amnion/ chorion/ decidua	Expansion/passages/senescence; Oxygen/process environment	NR
Jo, Chris H. (2008)	Fetal mesenchymal stem cells derived from human umbilical cord sustain primitive characteristics during extensive expansion	*Cell and Tissue Research*	Umbilical cord/ Wharton's jelly	Starting material/source; Expansion/passages/senescence	10.1007/s00441-008-0696-3
Battula, Venkata Lokesh (2008)	Prospective isolation and characterization of mesenchymal stem cells from human placenta using a frizzled-9-specific monoclonal antibody	*Differentiation*	Placenta/ amnion/ chorion/ decidua	Isolation/tissue processing	10.1111/j.1432-0436.2007.00225.x
Pereira, Winston Costa (2008)	Reproducible methodology for the isolation of mesenchymal stem cells from human umbilical cord and its potential for cardiomyocyte generation	*Journal of Tissue Engineering and Regenerative Medicine*	Umbilical cord/ Wharton's jelly	Starting material/source; Isolation/tissue processing	10.1002/term.107
Miao, Zongning (2006)	Isolation of mesenchymal stem cells	*Cell Biology International*	Placenta/ amnio	Isolation/tissue processing	10.1016/j.cellbi.2006.03.009

	from human placenta: Comparison with human bone marrow mesenchymal stem cells		n/ chorio n/ decidu a		
in't Anker, PS (2004)	Isolation of mesenchymal stem cells of fetal or maternal origin from human placenta	*Stem Cells*	Placent a/ amnio n/ chorio n/ decidu a	Starting material/source; Isolation/tissue processing	10.1634/stemcells.2004-0058
Martins JP (2014)	Towards an advanced therapy medicinal product based on mesenchymal stromal cells isolated from the umbilical cord tissue: quality and safety data	*Stem Cell Research & Therapy*	Umbili cal cord/ Whart on's jelly	Isolation/tissue processing; Media/supplements; Expansion/passages/se nescence; Cryopreservation/stor age; GMP/quality/release; Potency/functional CQA	10.1186/scrt398
Neumann A (2014)	Characterizati on and Application of a Disposable Rotating Bed Bioreactor for Mesenchymal Stem Cell Expansion	*Bioengineeri ng*	Umbili cal cord/ Whart on's jelly	Expansion/passages/se nescence; Scale-up/3D/bioreactor	10.3390/bioengineering1040 231
Osiecki MJ (2015)	Packed Bed Bioreactor for the Isolation and Expansion of Placental-Derived Mesenchymal Stromal Cells	*PLOS ONE*	Placent a/ amnio n/ chorio n/ decidu a	Isolation/tissue processing; Expansion/passages/se nescence; Scale-up/3D/bioreactor	10.1371/journal.pone.014494 1
Kong CM (2019)	Manufacturin g of human Wharton's jelly stem cells for clinical use: selection of serum is important	*Cytotherapy*	Umbili cal cord/ Whart on's jelly	Media/supplements; GMP/quality/release; Potency/functional CQA	10.1016/j.jcyt.2019.02.008

Zhao, Lingjia (2026)	Basal Medium Affects the Biological Characteristics and Immunomodulatory Functions of MSCs	*Applied Biochemistry and Biotechnology*	Umbilical cord/ Wharton's jelly	Media/supplements; Expansion/passages/senescence; Potency/functional CQA	10.1007/s12010-026-05727-5
Nguyen, Thanh-Truc (2026)	Contamination rates in umbilical cord Mesenchymal stromal cell cryopreservation: a report from Vinmec tissue bank	*Cell and Tissue Banking*	Umbilical cord/ Wharton's jelly	GMP/quality/release	10.1007/s10561-026-10221-7
Liu L. (2012)	A novel way to isolate MSCs from umbilical cords	*European Journal of Immunology*	Umbilical cord/ Wharton's jelly	Isolation/tissue processing	10.1002/eji.201142356
Huang Q. (2019)	An efficient protocol to generate placental chorionic plate-derived mesenchymal stem cells with superior proliferative and immunomodulatory properties	*Stem Cell Research & Therapy*	Placenta/ amnion/ chorion/ decidua	Starting material/source; Isolation/tissue processing; Media/supplements; Potency/functional CQA	10.1186/s13287-019-1405-8

Table S4A. Distribution of full-text exclusions by primary reason (n = 208).

**Primary exclusion reason**	**n**
No extractable manufacturing/process comparison or CQA outcome	84
Contextual biological/mechanistic study without an eligible manufacturing-process endpoint	69
Other cell product/source outside scope	14
MSC used as effector/support for another cell population	11
Differentiation/mechanistic study without eligible manufacturing outcome	9
Mechanistic/molecular study without direct manufacturing endpoint	7
Therapeutic/clinical study without eligible manufacturing outcome	6
Methods/protocol/book chapter without eligible primary results	4
Non-human/animal study	2
Review/update rather than primary manufacturing study	1
Duplicate/translated version of included report V0119	1

Table S4B. Report-level full-text exclusions (n = 208).

Citation	Title	DOI	Primary exclusion reason
Kim, Young Eun (2026)	Characterization and Validation of Long-Term Cultured TERT-Immortalized Human Wharton's Jelly-Derived Mesenchymal Stromal Cells	10.1007/s12015-026-11118-4	No extractable manufacturing/process comparison or CQA outcome
Rinendyaputri, Ratih (2026)	Characterization of Hypoxia-Preconditioned Umbilical Cord Mesenchymal Stem Cells Derived From Macaca fascicularis	10.1111/jmp.70056	Non-human/animal study
Yang, Song (2025)	An engineered hypoxia-response promoter for human umbilical cord-derived mesenchymal stem cell-based therapeutics	10.1186/s12896-025-00993-3	No extractable manufacturing/process comparison or CQA outcome
Shuhaimi, Nur Athirah Ahmad (2025)	Dentinogenic Effect of BMP-7 on Wharton's Jelly Mesenchymal Stem Cells Cultured in Decellularized Dental Pulp	10.3390/ijms262311760	No extractable manufacturing/process comparison or CQA outcome
Liu, Wei (2025)	Distinct molecular properties and functions of small EV subpopulations isolated from human umbilical cord MSCs using tangential flow filtration combined with size exclusion chromatography	10.1002/jev2.70029	No extractable manufacturing/process comparison or CQA outcome
Ren S. (2025)	Effect of human umbilical cord mesenchymal stem cells co-culture combined with ginsenoside rg1 on heart failure cell model	10.12307/2025.556	Therapeutic/clinical study without eligible manufacturing outcome
Le, Thuan Minh (2025)	Efficient Enrichment of Muse Cells from Human Umbilical Cord Mesenchymal Stem Cells Using Collagenase and Hypoxic Stress	10.15419/bmrat.v12i4.968	Other cell product/source outside scope
Liu, Shanshan (2025)	Isolation of Human BAMBIhighMFGE8high Umbilical Cord Derived Mesenchymal Stromal Cells-	10.3791/67545	No extractable manufacturing/process comparison or CQA outcome
Mujde, Cem (2025)	Three-Dimensional Cartilage Tissue Engineering Using Placenta-Derived Extra-Embryonic Mesenchymal Stem Cells: From Isolation to Differentiation	10.3390/biomedicines13092291	No extractable manufacturing/process comparison or CQA outcome
Baradaran, Behnoosh (2025)	Umbilical cord-derived mesenchymal stem cell condition medium effect on rotavirus-infected Caco-2 cells	10.1016/j.tice.2024.102699	No extractable manufacturing/process comparison or CQA outcome

	survival and inflammatory responses		
Kuntjoro, Mefina (2024)	Cobalt Chloride as a Hypoxia Mimicking Agent Induced HIF-1α and mTOR Expressions of Human Umbilical Cord Mesenchymal Stem Cells	10.1590/pboci.2024.020	Mechanistic/molecular study without direct manufacturing endpoint
Wu M. (2024)	Effect of culture supernatant of human umbilical cord mesenchymal stem cells on survival, apoptosis and endometrium receptivity of human endometrial stromal cells after treated with mifepristone	10.13481/j.1671-587x.20240110	Other cell product/source outside scope
Kosuthova, Hedviga (2024)	Effects of Replicative Senescence of Human Chorionic MSCs on their EV-miRNA Profile	10.1007/s12015-024-10790-8	No extractable manufacturing/process comparison or CQA outcome
Li R. (2024)	Erythropoietin-overexpressed umbilical cord mesenchymal stem cells inhibit neuroapoptosis in ischemic-hypoxic SH-SY5Y and its mechanism	10.12307/2024.704	No extractable manufacturing/process comparison or CQA outcome
Xia H. (2024)	Targeted induction of human umbilical cord mesenchymal stem cells cultured with human peripheral blood serum into neural stem cells	10.12307/2024.194	Differentiation/mechanistic study without eligible manufacturing outcome
Seok, Jin (2024)	The potent paracrine effect of umbilical cord mesenchymal stem cells mediates mitochondrial quality control to restore chemotherapy-induced damage in ovarian granulosa cells	10.1016/j.biopha.2024.116263	Therapeutic/clinical study without eligible manufacturing outcome
Xue, Lulu (2024)	Transplantation of human placental chorionic plate-derived mesenchymal stem cells for repair of neurological damage in neonatal hypoxic–ischemic encephalopathy	10.4103/1673-5374.390952	No extractable manufacturing/process comparison or CQA outcome
Yang Z.-L. (2024)	Whole-transcriptome profiling reveals potential biomarkers for the reversal of thymic epithelial cell senescence by umbilical cord mesenchymal stem cells	10.18632/aging.205738	No extractable manufacturing/process comparison or CQA outcome
Yang, Zai-Ling (2024)	Whole-transcriptome profiling reveals potential biomarkers for the reversal of thymic epithelial cell senescence by umbilical cord mesenchymal stem cells	NR	No extractable manufacturing/process comparison or CQA outcome

Habibie Y.A. (2023)	Effect of umbilical cord mesenchymal stem cells on hypoxia-inducible factor-1 alpha (HIF-1α) production in arteriovenous fistula (AVF) animal model: A preliminary study	10.52225/narra.v3i3.225	Mechanistic/molecular study without direct manufacturing endpoint
Sreekumar, T. R. (2023)	Evaluation of Culture Morphology of Neuronally Transdifferentiated Wharton's Jelly Derived Mesenchymal Stem Cells	10.18805/ijar.b-4365	No extractable manufacturing/process comparison or CQA outcome
Bonilla, Ximena (2023)	Mesenchymal Stromal Cells from Perinatal Tissues as an Alternative for Ex Vivo Expansion of Hematopoietic Progenitor and Stem Cells from Umbilical Cord Blood	10.3390/ijms242115544	MSC used as effector/support for another cell population
Kurenkova, Anastasiia D. (2023)	Resveratrol's Impact on the Chondrogenic Reagents' Effects in Cell Sheet Cultures of Wharton's Jelly-Derived MSCs	10.3390/cells12242845	No extractable manufacturing/process comparison or CQA outcome
Sadrifar S. (2023)	The effect of fresh frozen plasma (FFP) and human platelet lysate (HPL) on the proliferation rate of human mesenchymal stem cells (MSCs) derived from adipose tissue and umbilical cord blood	NR	Other cell product/source outside scope
Lamparelli, Erwin Pavel (2022)	3D in vitro cultures of human bone marrow and Wharton's jelly derived mesenchymal stromal cells show high chondrogenic potential	10.3389/fbioe.2022.986310	No extractable manufacturing/process comparison or CQA outcome
Bao, Bihui (2022)	Co-culture induces expression of female primordial germ cell-specific genes in human Wharton's jelly-derived mesenchymal stem cells	NR	MSC used as effector/support for another cell population
Huang, Jiawei (2022)	Human pluripotent stem cell-derived ectomesenchymal stromal cells promote more robust functional recovery than umbilical cord-derived mesenchymal stromal cells after hypoxic–ischaemic brain damage	10.7150/thno.57234	No extractable manufacturing/process comparison or CQA outcome
Nagamura-Inoue, Tokiko (2022)	Immunological influence of serum-free manufactured umbilical cord-derived mesenchymal stromal cells for steroid-resistant acute graft-versus-host disease	10.1007/s12185-022-03408-7	Therapeutic/clinical study without eligible manufacturing outcome

Rianti D. (2022)	The Characteristics and Potency of Limestone-based carbonate hydroxyapatite to Viability and Proliferation of Human Umbilical Cord Mesenchymal Stem Cell	10.52711/0974-360x.2022.00380	No extractable manufacturing/process comparison or CQA outcome
Zhang H. (2021)	Dual Effects of Hypoxia-Inducible Factors-1 Alpha in Bleomycin-Induced Pulmonary Fibrosis Treated by Human Umbilical Cord Mesenchymal Stem Cells	10.1155/2021/6658855	No extractable manufacturing/process comparison or CQA outcome
Kouchakian M.-R. (2021)	Human umbilical cord mesenchymal stem cells express cholinergic neuron markers during co-culture with amniotic membrane cells and retinoic acid induction	10.47176/mjiri.35.129	MSC used as effector/support for another cell population
Widowati W. (2021)	Hypoxic and normoxic-human Wharton's Jelly mesenchymal stem cell-free lysate for anticancer therapies	10.48048/wjst.2021.9270	No extractable manufacturing/process comparison or CQA outcome
Luo, Wei (2021)	NGF nanoparticles enhance the potency of transplanted human umbilical cord mesenchymal stem cells for myocardial repair	10.1152/ajpheart.00855.2020	Therapeutic/clinical study without eligible manufacturing outcome
Shilina M.A. (2020)	Characterization of Cultured Mesenchymal Stromal Cells Established from Human Chorion	10.1134/s1990519x20030050	No extractable manufacturing/process comparison or CQA outcome
Ling-Ling, E. (2020)	Combined Use of Recombinant Human Bone Morphogenetic Protein-7 and Osteogenic Media May Have No Ideal Synergistic Effect on Bone Regeneration of Human Umbilical Cord Mesenchymal Stem Cells Seeded on Nanohydroxyapatite/Collagen/ Poly (l-Lactide)	10.1089/scd.2020.0066	No extractable manufacturing/process comparison or CQA outcome
Liu X.C. (2020)	Effect of human Wharton's Jelly or adipose derived mesenchymal stem cells culture supernatant on endothelial cells angiogenesis	10.3760/cma.j.issn.0376-2491.2020.06.012	No extractable manufacturing/process comparison or CQA outcome
Trung Kien Do (2020)	Efficient Isolation and Long-term Red Fluorescent Nanodia-mond Labeling of Umbilical Cord Mesenchymal Stem Cells for the Effective Differentiation into Hepatocyte-like Cells	10.1590/1678-4324-2020200082	Differentiation/mechanistic study without eligible manufacturing outcome
Wu P.-P. (2020)	FOXO4 maintains senescence in human umbilical cord mesenchymal stem cells by	10.13294/j.aps.2020.0045	No extractable manufacturing/process

	repressing apoptosis; [FOXO4 通过抑制凋亡维持人脐带间充质干细胞衰老]		comparison or CQA outcome
Li Y. (2020)	Human umbilical cord mesenchymal stem cells differentiate into neuron-like cells after induction with B27-supplemented serum-free medium	10.12122/j.issn.1673-4254.2020.09.18	No extractable manufacturing/process comparison or CQA outcome
Huang Y. (2020)	Proliferation and apoptosis of chondrocytes co-cultured with TDP43 lentivirus transfected-humanumbilical cord mesenchymal stem cells	10.3969/j.issn.2095-4344.2027	No extractable manufacturing/process comparison or CQA outcome
Sun, Changbin (2020)	Single-cell RNA-seq highlights heterogeneity in human primary Wharton's jelly mesenchymal stem/stromal cells cultured in vitro; Single-cell RNA-seq highlights heterogeneity in human primary Wharton’s Jelly mesenchymal stem/stromal cells cultured in vitro	10.1186/s13287-020-01660-4	No extractable manufacturing/process comparison or CQA outcome
Sun, Xunsha (2019)	Altered expression of circular RNAs in human placental chorionic plate-derived mesenchymal stem cells pretreated with hypoxia	10.1002/jcla.22825	No extractable manufacturing/process comparison or CQA outcome
Weiwei L. (2019)	CD4+T and Treg immunomodulatory functions after culture with the supernatant of human placenta mesenchymal stem cells	10.3969/j.issn.2095-4344.0696	No extractable manufacturing/process comparison or CQA outcome
Van, Se Young (2019)	Human umbilical cord blood mesenchymal stem cells expansion via human fibroblast-derived matrix and their potentials toward regenerative application	10.1007/s00441-018-2971-2	Other cell product/source outside scope
Gounari, Eleni (2019)	Isolation of a novel embryonic stem cell cord blood-derived population with in vitro hematopoietic capacity in the presence of Wharton's jelly-derived mesenchymal stromal cells	10.1016/j.jcyt.2018.11.006	Other cell product/source outside scope
Svirshchevskaya, E. V. (2018)	Activation of NK Cells in Mixed Cultures of Wharton's Jelly Mesenchymal Stromal Cells and Peripheral Blood Lymphocytes	10.1007/s10517-018-3985-1	No extractable manufacturing/process comparison or CQA outcome

Yin J.-J. (2018)	Hypobaric Hypoxia Induces Autophagy of Umbilical Cord Mesenchymal Stem Cells	10.7534/j.issn.1009-2137.2018.04.041	Mechanistic/molecular study without direct manufacturing endpoint
Najar, Mehdi (2018)	Immunological impact of Wharton's Jelly mesenchymal stromal cells and natural killer cell co-culture	10.1007/s11010-018-3297-9	MSC used as effector/support for another cell population
Winkler, Tobias (2018)	Immunomodulatory placental-expanded, mesenchymal stromal cells improve muscle function following hip arthroplasty; Placental-expanded, mesenchymal cells improve muscle function following hip arthroplasty	10.1002/jcsm.12316	No extractable manufacturing/process comparison or CQA outcome
Ma, Caiyun (2018)	Isolation and Biological Characterization of Umbilical Cord Mesenchymal Stem Cells Derived from Goose Embryo	10.1166/jbt.2018.1824	Non-human/animal study
Mukai, Takeo (2018)	Umbilical Cord-Derived Mesenchymal Stromal Cells Contribute to Neuroprotection in Neonatal Cortical Neurons Damaged by Oxygen-Glucose Deprivation	10.3389/fneur.2018.00466	Therapeutic/clinical study without eligible manufacturing outcome
Fu X. (2017)	Anti-oxidative activities of supernatants of human fetal placental mesenchymal stem cells cultured in serum-free medium	10.3969/j.issn.2095-4344.2017.05.020	No extractable manufacturing/process comparison or CQA outcome
Wu, Hao (2017)	Comparison of nucleus pulposus stem/progenitor cells isolated from degenerated intervertebral disks with umbilical cord derived mesenchymal stem cells	10.1016/j.yexcr.2017.10.034	No extractable manufacturing/process comparison or CQA outcome
Poltavtsev, A. M. (2017)	Cytokine Production in Mixed Cultures of Mesenchymal Stromal Cells from Wharton's Jelly and Peripheral Blood Lymphocytes	10.1007/s10517-017-3759-1	Mechanistic/molecular study without direct manufacturing endpoint
Zhou B. (2017)	Effects of combined application of culture supernatant of human umbilical cord mesenchymal stem cells and ciprofloxacin on Staphylococcus aureus in vitro	10.3760/cma.j.issn.1009-2587.2017.06.014	No extractable manufacturing/process comparison or CQA outcome
Nam, Sang Min (2017)	Ex Vivo Expansion of Human Limbal Epithelial Cells Using Human Placenta-Derived and Umbilical Cord-Derived Mesenchymal Stem Cells	10.1155/2017/4206187	No extractable manufacturing/process comparison or CQA outcome
Wang, Jingjing (2017)	Expression of long non-coding RNAs in human bone marrow	10.3892/mmr.2017.7465	No extractable manufacturing/process

	mesenchymal stem cells co-cultured with human amnion-derived mesenchymal stem cells		comparison or CQA outcome
Gao, Liyang (2017)	Glycogen synthase kinase 3 (GSK3)-inhibitor SB216763 promotes the conversion of human umbilical cord mesenchymal stem cells into neural precursors in adherent culture	10.1007/s13577-016-0146-6	Differentiation/mechanistic study without eligible manufacturing outcome
Zhang, Chunli (2017)	Human amnion-derived mesenchymal stem cells protect against UVA irradiation-induced human dermal fibroblast senescence, in vitro	10.3892/mmr.2017.6795	No extractable manufacturing/process comparison or CQA outcome
Fajardo-Orduna, Guadalupe R. (2017)	Human Mesenchymal Stem/Stromal Cells from Umbilical Cord Blood and Placenta Exhibit Similar Capacities to Promote Expansion of Hematopoietic Progenitor Cells In Vitro	10.1155/2017/6061729	MSC used as effector/support for another cell population
Yang, Zhou Xin (2017)	Human umbilical cord mesenchymal stem cells increase interleukin-9 production of CD4+ T cells	10.3892/etm.2017.4952	No extractable manufacturing/process comparison or CQA outcome
Smith J.R. (2017)	Human umbilical cord mesenchymal stromal cell isolation, expansion, cryopreservation, and characterization	10.1002/cpsc.24	Methods/protocol/book chapter without eligible primary results
Caseiro A.R. (2017)	Neuro-muscular regeneration using scaffolds with mesenchymal stem cells (MSCs) isolated from human umbilical cord Wharton's jelly	10.1016/j.ctmat.2016.04.003	No extractable manufacturing/process comparison or CQA outcome
Poltavtsev, A. M. (2017)	Proliferation of Peripheral Blood Lymphocytes and Mesenchymal Stromal Cells Derived from Wharton's Jelly in Mixed and Membrane-Separated Cultures	10.1007/s10517-017-3846-3	No extractable manufacturing/process comparison or CQA outcome
Pawitan J.A. (2017)	Prospect of umbilical cord mesenchymal stem cell culture waste in regenerative medicine	NR	No extractable manufacturing/process comparison or CQA outcome
Cason, Carolina (2017)	SV40 Infection of Mesenchymal Stromal Cells From Wharton's Jelly Drives the Production of Inflammatory and Tumoral Mediators	10.1002/jcp.25723	No extractable manufacturing/process comparison or CQA outcome
Ruan G.-P. (2017)	Tree shrew umbilical cord mesenchymal stem cells:	10.3969/j.issn.2095-4344.2017.09.012	No extractable manufacturing/process

	Isolation, cultivation and osteogenic and adipogenic differentiation		comparison or CQA outcome
Khodabandeh Z. (2016)	Comparison of the expression of hepatic genes by human Wharton's Jelly Mesenchymal stem cells cultured in 2D and 3D Collagen culture systems	NR	No extractable manufacturing/process comparison or CQA outcome
Yang J. (2016)	Condition medium of HepG-2 cells induces the transdifferentiation of human umbilical cord mesenchymal stem cells into cancerous mesenchymal stem cells	NR	No extractable manufacturing/process comparison or CQA outcome
Yang, Juan (2016)	Condition medium of HepG-2 cells induces the transdifferentiation of human umbilical cord mesenchymal stem cells into cancerous mesenchymal stem cells	NR	No extractable manufacturing/process comparison or CQA outcome
Al Madhoun, Ashraf (2016)	Defined three-dimensional culture conditions mediate efficient induction of definitive endoderm lineage from human umbilical cord Wharton's jelly mesenchymal stem cells	10.1186/s13287-016-0426-9	Differentiation/mechanistic study without eligible manufacturing outcome
Wu Q. (2016)	Effects of culture supernatant of human amnion mesenchymal stem cells on biological characteristics of human fibroblasts	10.3760/cma.j.issn.1009-2587.2016.06.013	No extractable manufacturing/process comparison or CQA outcome
Ren, S. (2016)	Human umbilical cord derived mesenchymal stem cells promote interleukin-17 production from human peripheral blood mononuclear cells of healthy donors and systemic lupus erythematosus patients	10.1111/cei.12737	No extractable manufacturing/process comparison or CQA outcome
Yang, Hongna (2016)	Human umbilical cord-derived mesenchymal stem cells suppress proliferation of PHA-activated lymphocytes in vitro by inducing CD4+CD25highCD45RA+ regulatory T cell production and modulating cytokine secretion	10.1016/j.cellimm.2016.01.002	MSC used as effector/support for another cell population
Gu, Qiaoli (2016)	Hypoxia Promotes Osteogenesis of Human Placental-Derived Mesenchymal Stem Cells	10.1620/tjem.239.287	No extractable manufacturing/process comparison or CQA outcome
Fu, Xuejie (2016)	Improved osteogenesis and upregulated immunogenicity in	10.1186/s13287-016-0400-6	No extractable manufacturing/process

	human placenta-derived mesenchymal stem cells primed with osteogenic induction medium		comparison or CQA outcome
Xie B. (2016)	Therapeutic effects of human umbilical cord mesenchymal stem cells transplantation on hypoxic–ischemic encephalopathy	NR	No extractable manufacturing/process comparison or CQA outcome
Xie, Bingchuan (2016)	Therapeutic effects of human umbilical cord mesenchymal stem cells transplantation on hypoxic–ischemic encephalopathy	NR	No extractable manufacturing/process comparison or CQA outcome
Kadekar, Darshana (2015)	Differential ability of MSCs isolated from placenta and cord as feeders for supporting ex vivo expansion of umbilical cord blood derived CD34+ cells	10.1186/s13287-015-0194-y	MSC used as effector/support for another cell population
Watson N. (2015)	Discarded Wharton jelly of the human umbilical cord: A viable source for mesenchymal stromal cells	10.1016/j.jcyt.2014.08.009	Review/update rather than primary manufacturing study
Asgari, Hamid Reza (2015)	Human Wharton's jelly-derived mesenchymal stem cells express oocyte developmental genes during co-culture with placental cells	NR	Differentiation/mechanistic study without eligible manufacturing outcome
Asgari H.R. (2015)	Human Wharton's jelly-derived mesenchymal stem cells express oocyte developmental genes during co-culture with placental cells	NR	Differentiation/mechanistic study without eligible manufacturing outcome
Zhang, Y. J. (2015)	Importance of CD44 on umbilical cord mesenchymal stem cells for expansion of hematopoietic cells	10.14715/cmb/2015.61.2.4	MSC used as effector/support for another cell population
Graham C.D. (2015)	Isolation of mesenchymal stem cells from amniotic fluid and placenta	10.1002/9780470151808.sc01e02s35	No extractable manufacturing/process comparison or CQA outcome
Tsagias, Nikos (2015)	Pluripotent stem cells isolated from umbilical cord form embryonic like bodies in a mesenchymal layer culture	10.14670/hh-30.373	No extractable manufacturing/process comparison or CQA outcome
Wei X.-J. (2015)	Silymarin Protects Umbilical Cord-Derived Mesenchymal Stem Cells against Apoptosis Induced by Serum-Deprivation	10.7534/j.issn.1009-2137.2015.05.038	No extractable manufacturing/process comparison or CQA outcome
Yang, Y. (2014)	CD29 of human umbilical cord mesenchymal stem cells is required for expansion of CD34+ cells	10.1111/cpr.12130	MSC used as effector/support for another cell population

Thapsukhon, Boontharika (2014)	Effect of topology of poly(L-lactide-co-ε-caprolactone) scaffolds on the response of cultured human umbilical cord Wharton's jelly-derived mesenchymal stem cells and neuroblastoma cell lines	10.1080/09205063.2014.918457	No extractable manufacturing/process comparison or CQA outcome
Maslova, E. V. (2014)	Enrichment of Umbilical Cord Blood Mononuclears with Hemopoietic Precursors in Co-Culture with Mesenchymal Stromal Cells from Human Adipose Tissue	10.1007/s10517-014-2400-9	MSC used as effector/support for another cell population
Li M. (2014)	Human umbilical cord mesenchymal stem cells: Isolation, identification and multi-directional differentiation	10.3969/j.issn.2095-4344.2014.19.010	No extractable manufacturing/process comparison or CQA outcome
Li, Dongjie (2014)	Human umbilical cord-derived mesenchymal stem cells differentiate into epidermal-like cells using a novel co-culture technique	10.1007/s10616-013-9569-z	MSC used as effector/support for another cell population
Chatterjee, Debanjana (2014)	Human umbilical cord-derived mesenchymal stem cells utilise activin-A to suppress interferon-gamma production by natural killer cells	10.3389/fimmu.2014.00662	No extractable manufacturing/process comparison or CQA outcome
Choo, Kong Bung (2014)	Oxidative Stress-Induced Premature Senescence in Wharton's Jelly-Derived Mesenchymal Stem Cells	10.7150/ijms.8356	No extractable manufacturing/process comparison or CQA outcome
Huang X. (2014)	Umbilical cord-derived mesenchymal stem cell culture: Dyeing and tracer technique	10.3969/j.issn.2095-4344.2014.23.024	No extractable manufacturing/process comparison or CQA outcome
Yang J. (2013)	Comparative study on combined culture of human placenta-derived mesenchymal stem cells and human umbilical vein endothelial cells from same and different individuals	NR	No extractable manufacturing/process comparison or CQA outcome
Huang Z.-F. (2013)	Inhibitory effect of human umbilical cord-derived mesenchymal stem cells on interleukin-17 production in peripheral blood T cells from spondyloarthritis patients	10.7534/j.issn.1009-2137.2013.02.041	No extractable manufacturing/process comparison or CQA outcome
Ju, Xing-Ai (2013)	A slowly proliferating subpopulation in human umbilical cord mesenchymal stem cells in culture	10.1007/s11626-013-9654-9	No extractable manufacturing/process comparison or CQA outcome
Liu, Qiuying (2013)	Conditioned serum-free medium from umbilical cord	10.1007/s10529-013-1242-2	No extractable manufacturing/process

	mesenchymal stem cells has anti-photoaging properties		comparison or CQA outcome
Chon, Brian H. (2013)	Human umbilical cord mesenchymal stromal cells exhibit immature nucleus pulposus cell phenotype in a laminin-rich pseudo-three-dimensional culture system	10.1186/scrt331	No extractable manufacturing/process comparison or CQA outcome
Li H. (2013)	Human umbilical cord-derived mesenchymal stem cells co-cultured with hepatocytes can differentiate into hepatocyte-like cells	10.3969/j.issn.2095-4344.2013.32.005	Differentiation/mechanistic study without eligible manufacturing outcome
Lee, Youjin (2013)	Increased SCF/c-kit by hypoxia promotes autophagy of human placental chorionic plate-derived mesenchymal stem cells via regulating the phosphorylation of mTOR	10.1002/jcb.24303	Mechanistic/molecular study without direct manufacturing endpoint
Rossignoli, Filippo (2013)	Isolation, Characterization, and Transduction of Endometrial Decidual Tissue Multipotent Mesenchymal Stromal/Stem Cells from Menstrual Blood	10.1155/2013/901821	Other cell product/source outside scope
Li L.N. (2012)	Supportive effects of conditioned culture media of human umbilical cord mesenchymal stem cells on hematopoiesis in vitro.	NR	No extractable manufacturing/process comparison or CQA outcome
Lu, Guo-hui (2012)	Human placental decidua basalis-derived mesenchymal stem cells differentiate into dopamine neuron-like cells with no response to long-term culture in vitro	10.1097/wnr.0b013e328353fbb4	No extractable manufacturing/process comparison or CQA outcome
Ding, Dah-Ching (2012)	Human Umbilical Cord Mesenchymal Stem Cells Support Nontumorigenic Expansion of Human Embryonic Stem Cells	10.3727/096368912x647199	Other cell product/source outside scope
Kang, Mi-Na (2012)	Human Umbilical Cord-Derived Mesenchymal Stem Cells Differentiate into Ligament-Like Cells with Mechanical Stimulation in Various Media	10.1007/s13770-012-0333-9	No extractable manufacturing/process comparison or CQA outcome
Wang D. (2012)	IL-6 production stimulated by CD14 monocytes-paracrined IL-1β does not contribute to the immunosuppressive activity of human umbilical cord mesenchymal stem cells	10.1159/000338509	No extractable manufacturing/process comparison or CQA outcome
Yust-Katz, Shlomit (2012)	Placental mesenchymal stromal cells induced into neurotrophic	10.3109/14653249.2011.613928	No extractable manufacturing/process

	factor-producing cells protect neuronal cells from hypoxia and oxidative stress		comparison or CQA outcome
Grabowska I. (2012)	Restricted myogenic potential of mesenchymal stromal cells isolated from umbilical cord	10.3727/096368912x640493	No extractable manufacturing/process comparison or CQA outcome
Zhang, Xiaohong (2011)	Isolation and Characterization of Mesenchymal Stem Cells From Human Umbilical Cord Blood: Reevaluation of Critical Factors for Successful Isolation and High Ability to Proliferate and Differentiate to Chondrocytes as Compared to Mesenchymal Stem Cells From Bone Marrow and Adipose Tissue	10.1002/jcb.23042	Other cell product/source outside scope
Zhao Z.-Q. (2010)	Cardiomyogenesis of human umbilical cord mesenchymal stem cells induced by conditional culture in the presence of sphingosine-1-phosphate	10.3969/j.issn.1673-8225.2010.36.041	No extractable manufacturing/process comparison or CQA outcome
He S.-Q. (2010)	Isolation of human umbilical cord mesenchymal stem cells and differentiation into adipocytes and osteblasts	10.3969/j.issn.1673-8225.2010.14.005	No extractable manufacturing/process comparison or CQA outcome
Manochant S. (2010)	Isolation, characterization and neural differentiation potential of amnion derived mesenchymal stem cells	NR	Differentiation/mechanistic study without eligible manufacturing outcome
Jiang J. (2010)	Isolation, culture and differentiation of mesenchymal stem cells from Wharton's jelly of human umbilical cord	10.3969/j.issn.1673-8225.2010.10.005	No extractable manufacturing/process comparison or CQA outcome
Sun G.-D. (2010)	Isolation, culture and identification of mesenchymal stem cells from human umbilical cord as well as their osteogenic and adipogenic differentiation	NR	No extractable manufacturing/process comparison or CQA outcome
Sun G.-D. (2010)	Isolation, culture and osteogenic differentiation of mesenchymal stem cells from human umbilical cord: Ultrastructure of cell membrane observed using atomic force microscope	10.3969/j.issn.1673-8225.2010.01.008	No extractable manufacturing/process comparison or CQA outcome
Song, Chang Hoon (2010)	Stem Cell Scoring System for Selection Against Dominant Human Umbilical Cord-Derived Mesenchymal Stem Cell Undergoing Senescence	NR	Other cell product/source outside scope

Majore, Ingrida (2009)	Identification of subpopulations in mesenchymal stem cell-like cultures from human umbilical cord	10.1186/1478-811x-7-6	No extractable manufacturing/process comparison or CQA outcome
Miao Z.-N. (2009)	Location and isolation of mesenchymal stem cells in human placenta	10.3969/j.issn.1673-8225.2009.36.026	No extractable manufacturing/process comparison or CQA outcome
Liu D. (2009)	Optimal selection of culture systems for human umbilical cord bloos mesenchymal stem cells	NR	Other cell product/source outside scope
Nagase T. (2009)	Pericellular matrix of decidua-derived mesenchymal cells: A potent human-derived substrate for the maintenance culture of human ES cells	10.1002/dvdy.21944	No extractable manufacturing/process comparison or CQA outcome
Yang L.-M. (2008)	Biological characterization of human umbilical cord mesenchymal stem cells following tissue mass cell culture	NR	No extractable manufacturing/process comparison or CQA outcome
Kawasaki-Oyama, Rosa Sayoko (2008)	Blood mesenchymal stem cell culture from the umbilical cord with and without Ficoll-Paque density gradient method	NR	Other cell product/source outside scope
Kestendjieva S. (2008)	Characterization of mesenchymal stem cells isolated from the human umbilical cord	10.1016/j.cellbi.2008.02.002	No extractable manufacturing/process comparison or CQA outcome
Lü L.-L. (2008)	Cytokine production and hematopoiesis-supportive function of human umbilical cord mesenchymal stem cells	NR	Mechanistic/molecular study without direct manufacturing endpoint
Qiao, Chun (2008)	Human mesenchymal stem cells isolated from the umbilical cord	10.1016/j.cellbi.2007.08.002	No extractable manufacturing/process comparison or CQA outcome
Shyu K.-G. (2008)	Hyperbaric oxygen induces placental growth factor expression in bone marrow-derived mesenchymal stem cells	10.1016/j.lfs.2008.05.005	No extractable manufacturing/process comparison or CQA outcome
Tian X. (2008)	Isolation of multipotent mesenchymal stem cells from the tissue of umbilical cord for osteoblasts and adipocytes differentiation	NR	No extractable manufacturing/process comparison or CQA outcome
Steigman S.A. (2007)	Isolation of mesenchymal stem cells from amniotic fluid and placenta.	10.1002/9780470151808.sc01e02s1	No extractable manufacturing/process comparison or CQA outcome

Citation not available in source record	Stem Cell–Based Therapy for Coronavirus Disease 2019	NR	Therapeutic/clinical study without eligible manufacturing outcome
Citation not available in source record	Differentiation of mesenchymal stem cells into osteoblasts and immunohistochemical identification	NR	Differentiation/mechanistic study without eligible manufacturing outcome
Citation not available in source record	Histone H3K9 Methylation Features under Hypoxic Conditions after the HIF1A Knockdown in Mesenchymal Stromal Cells In Vitro	NR	Mechanistic/molecular study without direct manufacturing endpoint
Citation not available in source record	Isolation, Culture, Cryopreservation, and Preparation of Umbilical Cord-Derived Mesenchymal Stem Cells as a Final Cellular Product Under GMP-Compliant Conditions	NR	Methods/protocol/book chapter without eligible primary results
Citation not available in source record	Isolation and expansion of mesenchymal stromal/stem cells from umbilical cord under chemically defined conditions	NR	Methods/protocol/book chapter without eligible primary results
Citation not available in source record	Manufacture of clinical grade human placenta-derived multipotent mesenchymal stromal cells	NR	Methods/protocol/book chapter without eligible primary results
Citation not available in source record	An optimized procedure for preparation of conditioned medium from Wharton's jelly MSCs	NR	Other cell product/source outside scope
Citation not available in source record	Systemic delivery of large-scale manufactured Wharton's jelly MSC-derived extracellular vesicles improves cardiac function after myocardial infarction	NR	Other cell product/source outside scope
Citation not available in source record	Continuous collection of human MSC-derived extracellular vesicles from a stirred-tank reactor under xenogeneic-free conditions	NR	Other cell product/source outside scope
Koltsova A.M. (2020)	Isolation and characterization of mesenchymal stem cell lines from different parts of placenta of the same donor	10.31857/s0041377120090035	Duplicate/translated version of included report V0119
Fu, Jinhong (2026)	Deep Learning-Assisted Nanomechanical Evaluation of Replicative Senescence in Human Umbilical Cord Mesenchymal Stem Cells	10.1021/acs.langmuir.6c00374	Contextual biological/mechanistic study without an eligible manufacturing-process endpoint
Boonmuen, Nittaya (2026)	DPHD from Curcuma comosa Enhances the Expansion and Osteogenic Differentiation of	10.1021/acsomega.5c08638	Contextual biological/mechanistic study without an eligible

	Human Umbilical Cord-Derived Mesenchymal Stem Cells While Attenuating Senescence		manufacturing-process endpoint
Wang S. (2026)	PQQ Modulates Cell Cycle and DNA Replication Programs to Alleviate Replicative Senescence in Human Umbilical Cord Mesenchymal Stromal Cells	10.1007/s12015-025-11037-w	Contextual biological/mechanistic study without an eligible manufacturing-process endpoint
Wang, Li-Tzu (2026)	Three-dimensional (3D) culture-primed placental mesenchymal stem cells decrease cellular heterogeneity, significantly enhancing osteogenesis via improving mitochondrial function	10.1016/j.mtbio.2026.103012	Contextual biological/mechanistic study without an eligible manufacturing-process endpoint
Iwata, Yuma (2026)	Transcriptomic Profiling Reveals Mechanisms of Anoikis Resistance in Spheroid-Cultured Human Umbilical Cord Mesenchymal Stem Cells	10.1007/s13770-025-00779-w	Contextual biological/mechanistic study without an eligible manufacturing-process endpoint
Wang S.-Y. (2025)	Hydroxysafflor Yellow A Ameliorates the Replicative Senescence of Human Umbilical Cord Mesenchymal Stem Cells by Suppressing Oxidative Stress	10.19746/j.cnki.issn.1009-2137.2025.05.039	Contextual biological/mechanistic study without an eligible manufacturing-process endpoint
Liu, Hongjiang (2025)	Microencapsulated 3D culture of human umbilical cord-derived mesenchymal stem cells enhances their therapeutic effect on intrauterine adhesion	10.1093/stcltm/szaf044	Contextual biological/mechanistic study without an eligible manufacturing-process endpoint
Pochon, Cecile (2025)	Optimized GMP-grade Wharton's jelly's mesenchymal stromal cells manufacturing and administration protocol for Graft versus Host disease prevention	10.1016/j.retram.2025.103543	Contextual biological/mechanistic study without an eligible manufacturing-process endpoint
Rajendran, Ramya Lakshmi (2024)	Engineering Three-Dimensional Spheroid Culture for Enrichment of Proangiogenic miRNAs in Umbilical Cord Mesenchymal Stem Cells and Promotion of Angiogenesis	10.1021/acsomega.4c02037	Contextual biological/mechanistic study without an eligible manufacturing-process endpoint
Huang, Q. - M. (2024)	Long-term hypoxic atmosphere enhances the stemness, immunoregulatory functions, and therapeutic application of human umbilical cord mesenchymal stem cells	10.1302/2046-3758.1312.bjr-2024-0136.r2	Contextual biological/mechanistic study without an eligible manufacturing-process endpoint
Zhang Y. (2024)	Single cell RNA-sequencing reveals the cellular senescence	10.1016/j.placenta.2024.03.014	Contextual biological/mechanistic

	of placental mesenchymal stem/stromal cell in preeclampsia		study without an eligible manufacturing-process endpoint
Zhai, Huifang (2024)	Three-dimensional cultured human umbilical cord mesenchymal stem cells attenuate pulmonary fibrosis by improving the balance of mitochondrial fusion and fission	10.1093/stcltm/szae051	Contextual biological/mechanistic study without an eligible manufacturing-process endpoint
Carcopino, Charlotte (2024)	Understanding the Angiogenic Characteristics of Clinical-Grade Mesenchymal Stromal Cells Isolated from Human Umbilical Cord	10.1007/s12015-024-10712-8	Contextual biological/mechanistic study without an eligible manufacturing-process endpoint
Li, Hang (2023)	Hypoxia and inflammatory factor preconditioning enhances the immunosuppressive properties of human umbilical cord mesenchymal stem cells	10.4252/wjsc.v15.i11.999	Contextual biological/mechanistic study without an eligible manufacturing-process endpoint
Hejazi, Sajjad (2023)	Study of the cellular senescence process in human umbilical cord Wharton's jelly-derived mesenchymal stem cells	10.4103/aihb.aihb_19_23	Contextual biological/mechanistic study without an eligible manufacturing-process endpoint
Mo Y. (2022)	Biological characteristics of umbilical cord and menstrual blood-derived mesenchymal stem cells under physiological hypoxia	10.12307/2022.761	Contextual biological/mechanistic study without an eligible manufacturing-process endpoint
Gallo, Alessia (2022)	Changes in the Transcriptome Profiles of Human Amnion-Derived Mesenchymal Stromal/Stem Cells Induced by Three-Dimensional Culture: A Potential Priming Strategy to Improve Their Properties	10.3390/ijms23020863	Contextual biological/mechanistic study without an eligible manufacturing-process endpoint
Das, Monalisa (2022)	Comparative evaluation of proliferative potential and replicative senescence associated changes in mesenchymal stem cells derived from dental pulp and umbilical cord	10.1007/s10561-021-09926-8	Contextual biological/mechanistic study without an eligible manufacturing-process endpoint
Boss, Anna Leabourn (2022)	Full spectrum flow cytometry reveals mesenchymal heterogeneity in first trimester placentae and phenotypic convergence in culture, providing insight into the origins of placental mesenchymal stromal cells	10.7554/elife.76622	Contextual biological/mechanistic study without an eligible manufacturing-process endpoint

Pham, Viet Q. (2021)	Angiogenic potential of hypoxia preconditioned human adipose and umbilical cord-derived mesenchymal stem cells: a comparative study	10.23736/s2724-542x.21.02749-x	Contextual biological/mechanistic study without an eligible manufacturing-process endpoint
Feng, Xu-Dong (2021)	Hypoxia-inducible factor-1α-mediated upregulation of CD99 promotes the proliferation of placental mesenchymal stem cells by regulating ERK1/2	10.4252/wjsc.v13.i4.317	Contextual biological/mechanistic study without an eligible manufacturing-process endpoint
Wang, Quanlei (2021)	Single-cell transcriptome profiling reveals molecular heterogeneity in human umbilical cord tissue and culture-expanded mesenchymal stem cells	10.1111/febs.15834	Contextual biological/mechanistic study without an eligible manufacturing-process endpoint
Seok, Jin (2020)	Alteration of fatty acid oxidation by increased CPT1A on replicative senescence of placenta-derived mesenchymal stem cells	10.1186/s13287-019-1471-y	Contextual biological/mechanistic study without an eligible manufacturing-process endpoint
Silva A.D. (2020)	Comparison of senescence progression in mesenchymal cells from human umbilical cord walls measured by immunofluorescence and flow cytometry of p16 and p21	10.31744/einstein_journal/2020ao5236	Contextual biological/mechanistic study without an eligible manufacturing-process endpoint
Das, Ankita (2020)	Honey-incorporated nanofibre reduces replicative senescence of umbilical cord-derived mesenchymal stem cells	10.1049/iet-nbt.2019.0288	Contextual biological/mechanistic study without an eligible manufacturing-process endpoint
Kwon, Heejung (2020)	Hypoxia-Preconditioned Placenta-Derived Mesenchymal Stem Cells Rescue Optic Nerve Axons Via Differential Roles of Vascular Endothelial Growth Factor in an Optic Nerve Compression Animal Model	10.1007/s12035-020-01965-8	Contextual biological/mechanistic study without an eligible manufacturing-process endpoint
Keshtkar, Somayeh (2020)	Hypoxia-Preconditioned Wharton's Jelly-Derived Mesenchymal Stem Cells Mitigate Stress-Induced Apoptosis and Ameliorate Human Islet Survival and Function in Direct Contact Coculture System	10.1155/2020/8857457	Contextual biological/mechanistic study without an eligible manufacturing-process endpoint
Bobkov, Danila (2020)	Replicative senescence in MSCWJ-1 human umbilical cord mesenchymal stem cells is marked by characteristic changes in motility, cytoskeletal organization, and RhoA localization	10.1007/s11033-020-05476-6	Contextual biological/mechanistic study without an eligible manufacturing-process endpoint

Gong, Xiangcui (2020)	Three-Dimensional Culture of Umbilical Cord Mesenchymal Stem Cells Effectively Promotes Platelet Recovery in Immune Thrombocytopenia	10.1248/bpb.b19-01069	Contextual biological/mechanistic study without an eligible manufacturing-process endpoint
Hao, Dake (2019)	Hypoxic Preconditioning Enhances Survival and Proangiogenic Capacity of Human First Trimester Chorionic Villus-Derived Mesenchymal Stem Cells for Fetal Tissue Engineering	10.1155/2019/9695239	Contextual biological/mechanistic study without an eligible manufacturing-process endpoint
Silva-Cote, Ingrid (2019)	Strategy for the Generation of Engineered Bone Constructs Based on Umbilical Cord Mesenchymal Stromal Cells Expanded with Human Platelet Lysate	10.1155/2019/7198215	Contextual biological/mechanistic study without an eligible manufacturing-process endpoint
De Witte, Samantha F. H. (2018)	Epigenetic changes in umbilical cord mesenchymal stromal cells upon stimulation and culture expansion	10.1016/j.jcyt.2018.05.005	Contextual biological/mechanistic study without an eligible manufacturing-process endpoint
Taguchi, Takashi (2018)	Influence of hypoxia on the stemness of umbilical cord matrix-derived mesenchymal stem cells cultured on chitosan films	10.1002/jbm.b.33864	Contextual biological/mechanistic study without an eligible manufacturing-process endpoint
Lupatov A.Yu. (2017)	Comparison of the expression profile of surface molecular markers on mesenchymal stromal cell cultures isolated from human endometrium and umbilical cord	10.18097/pbmc20176301085	Contextual biological/mechanistic study without an eligible manufacturing-process endpoint
Sanap, Avinash (2017)	Herbal pre-conditioning induces proliferation and delays senescence in Wharton's Jelly Mesenchymal Stem Cells	10.1016/j.biopha.2017.06.107	Contextual biological/mechanistic study without an eligible manufacturing-process endpoint
Mathew, Suja Ann (2017)	Mesenchymal stromal cells isolated from gestationally diabetic human placenta exhibit insulin resistance, decreased clonogenicity and angiogenesis	10.1016/j.placenta.2017.09.002	Contextual biological/mechanistic study without an eligible manufacturing-process endpoint
Chatgilialoglu, Alexandros (2017)	Restored in vivo-like membrane lipidomics positively influence in vitro features of cultured mesenchymal stromal/stem cells derived from human placenta	10.1186/s13287-017-0487-4	Contextual biological/mechanistic study without an eligible manufacturing-process endpoint
Al-Sowayan, Balta (2017)	The effect of endothelial cell activation and hypoxia on placental chorionic	10.1016/j.placenta.2017.06.341	Contextual biological/mechanistic study without an eligible

	mesenchymal stem/stromal cell migration		manufacturing-process endpoint
Drela, Katarzyna (2016)	Enhanced neuro-therapeutic potential of Wharton's Jelly-derived mesenchymal stem cells in comparison with bone marrow mesenchymal stem cells culture	10.1016/j.jcyt.2016.01.006	Contextual biological/mechanistic study without an eligible manufacturing-process endpoint
Anastasiu D.M. (2016)	Explants-isolated human placenta and umbilical cord cells share characteristics of both epithelial and mesenchymal stem cells	NR	Contextual biological/mechanistic study without an eligible manufacturing-process endpoint
Du L. (2016)	Hypoxia enhances the healing effect of placental-derived mesenchymal stem cells on scald wound by promoting secretion of insulin-like growth factor-1	NR	Contextual biological/mechanistic study without an eligible manufacturing-process endpoint
Tong C. (2016)	Hypoxic pretreatment of human umbilical cord mesenchymal stem cells regulating macrophage polarization	10.11855/j.issn.0577-7402.2016.07.01	Contextual biological/mechanistic study without an eligible manufacturing-process endpoint
Mareschi, Katia (2016)	Immunoregulatory effects on T lymphocytes by human mesenchymal stromal cells isolated from bone marrow, amniotic fluid, and placenta	10.1016/j.exphem.2015.10.009	Contextual biological/mechanistic study without an eligible manufacturing-process endpoint
Zheng D. (2016)	Research on biological and genetic characteristics of human placenta mesenchymal stem cells cultured in vitro	10.3760/cma.j.issn.1003-9406.2016.04.009	Contextual biological/mechanistic study without an eligible manufacturing-process endpoint
Thaweesapphithak S. (2016)	The biological characteristics of placenta derived mesenchymal stem cells cultured in human serum	NR	Contextual biological/mechanistic study without an eligible manufacturing-process endpoint
Zhang, Li (2015)	Beneficial Effects of Hypoxic Preconditioning on Human Umbilical Cord Mesenchymal Stem Cells	10.4077/cjp.2015.bae369	Contextual biological/mechanistic study without an eligible manufacturing-process endpoint
Peltzer, Juliette (2015)	Heterogeneous Functions of Perinatal Mesenchymal Stromal Cells Require a Preselection Before Their Banking for Clinical Use	10.1089/scd.2014.0327	Contextual biological/mechanistic study without an eligible manufacturing-process endpoint
Koenig, Julia (2014)	Amnion-derived mesenchymal stromal cells show a mesenchymal–epithelial phenotype in culture	10.1007/s10561-013-9415-8	Contextual biological/mechanistic study without an eligible manufacturing-process endpoint

Yang, L. M. (2014)	Characterization of human umbilical cord mesenchymal stem cells following tissue mass culture	10.14715/cmb/2013.60.1.3	Contextual biological/mechanistic study without an eligible manufacturing-process endpoint
Du, Lili (2014)	Hypoxia Enhances Protective Effect of Placental-Derived Mesenchymal Stem Cells on Damaged Intestinal Epithelial Cells by Promoting Secretion of Insulin-Like Growth Factor-1	10.3390/ijms15021983	Contextual biological/mechanistic study without an eligible manufacturing-process endpoint
Shaer A. (2014)	Isolation and characterization of human mesenchymal stromal cells derived from placental decidua basalis; umbilical cord wharton's jelly and amniotic membrane	10.12669/pjms.305.4537	Contextual biological/mechanistic study without an eligible manufacturing-process endpoint
Roson-Burgo, Beatriz (2014)	Transcriptomic portrait of human Mesenchymal Stromal/Stem cells isolated from bone marrow and placenta	10.1186/1471-2164-15-910	Contextual biological/mechanistic study without an eligible manufacturing-process endpoint
Roszek, Katarzyna (2013)	Dramatic differences in activity of purines metabolizing ecto-enzymes between mesenchymal stem cells isolated from human umbilical cord blood and umbilical cord tissue	10.1139/bcb-2013-0050	Contextual biological/mechanistic study without an eligible manufacturing-process endpoint
Trivanovic, Drenka (2013)	Immunomodulatory capacity of human mesenchymal stem cells isolated from adipose tissue, dental pulp, peripheral blood and umbilical cord Wharton's jelly	10.5114/ceji.2013.39756	Contextual biological/mechanistic study without an eligible manufacturing-process endpoint
Feng K. (2013)	Isolation, culture and surface markers detection of human umbilical cord mesenchymal stem cells	10.3760/cma.j.issn.1009-9921.2013.06.011	Contextual biological/mechanistic study without an eligible manufacturing-process endpoint
Patel A.N. (2013)	Mesenchymal stem cell population isolated from the subepithelial layer of umbilical cord tissue	10.3727/096368912x655064	Contextual biological/mechanistic study without an eligible manufacturing-process endpoint
Trivanovic, Drenka (2013)	Mesenchymal Stem Cells Isolated from Peripheral Blood and Umbilical Cord Wharton's Jelly	10.2298/sarh1304178t	Contextual biological/mechanistic study without an eligible manufacturing-process endpoint
Zhu Y.Z. (2012)	[Effects of IFN-γ on immunomodulation of mesenchymal stem cells	NR	Contextual biological/mechanistic study without an eligible

	isolated from fetal side of human placenta tissues].		manufacturing-process endpoint
Koo, Bo Kyung (2012)	Isolation and Characterization of Chorionic Mesenchymal Stromal Cells from Human Full Term Placenta	10.3346/jkms.2012.27.8.857	Contextual biological/mechanistic study without an eligible manufacturing-process endpoint
Zeng, Hui-Lan (2011)	Hypoxia-mimetic agents inhibit proliferation and alter the morphology of human umbilical cord-derived mesenchymal stem cells	10.1186/1471-2121-12-32	Contextual biological/mechanistic study without an eligible manufacturing-process endpoint
Bacenkova, Darina (2011)	Isolation and basic characterization of human term amnion and chorion mesenchymal stromal cells	10.3109/14653249.2011.592522	Contextual biological/mechanistic study without an eligible manufacturing-process endpoint
Bai H. (2011)	Relations between reactive oxygen species and Raman spectral variations of Human Umbilical Cord Mesenchymal Stem cells with different viability	10.1134/s1054660x1111003x	Contextual biological/mechanistic study without an eligible manufacturing-process endpoint
Wang, Min (2010)	Clonal Isolation and Characterization of Mesenchymal Stem Cells from Human Amnion	10.1007/s12257-009-3147-4	Contextual biological/mechanistic study without an eligible manufacturing-process endpoint
Hollweck T. (2010)	Comparative analysis of adherence, viability, proliferation and morphology of umbilical cord tissue-derived mesenchymal stem cells seeded on different titanium-coated expanded polytetrafluoroethylene scaffolds	10.1088/1748-6041/5/6/065004	Contextual biological/mechanistic study without an eligible manufacturing-process endpoint
Macias, Maria I. (2010)	Isolation and characterization of true mesenchymal stem cells derived from human term decidua capable of multilineage differentiation into all 3 embryonic layers	10.1016/j.ajog.2010.06.045	Contextual biological/mechanistic study without an eligible manufacturing-process endpoint
Huang, Yong-Can (2009)	Isolation of Mesenchymal Stem Cells from Human Placental Decidua Basalis and Resistance to Hypoxia and Serum Deprivation	10.1007/s12015-009-9069-x	Contextual biological/mechanistic study without an eligible manufacturing-process endpoint
Barlow, Sarah (2008)	Comparison of Human Placenta- and Bone Marrow-Derived Multipotent Mesenchymal Stem Cells	10.1089/scd.2007.0154	Contextual biological/mechanistic study without an eligible manufacturing-process endpoint

Meyer, T. (2008)	Isolation and characterisation of mesenchymal stem cells in Wharton's jelly of the human umbilical cord: potent cells for cell-based therapies in pediatric surgery?	10.1007/s10353-008-0417-x	Contextual biological/mechanistic study without an eligible manufacturing-process endpoint
Lu L.-L. (2006)	Isolation and characterization of human umbilical cord mesenchymal stem cells with hematopoiesis-supportive function and other potentials	NR	Contextual biological/mechanistic study without an eligible manufacturing-process endpoint

Table S5A. Linked reports and study-family sensitivity handling.

Group	Reports	Evidence of overlap	Analytical handling
Study family 1	Clinical feasibility and cost efficiency of perinatal mesenchymal stem cell production under GMP conditions<br>Comprehensive evaluation of the clinical feasibility of using perinatal medical waste as a source for fetal mesenchymal stem cell banking under good manufacturing practice conditions	Full-text comparison showed the same three-author group, the same 32 term pregnancies and 160 perinatal samples, concordant recruitment structure, and overlapping manufacturing outcomes. The two reports were therefore judged to draw on the same underlying cohort.	Counted as one study family in the study-family sensitivity check; report-specific non-duplicated findings were retained narratively.
Translation duplicate	Isolation and Characterization of Mesenchymal Stem Cells Derived from Different Regions of the Placenta of the Same Donor<br>Isolation and characterization of mesenchymal stem cell lines from different parts of placenta of the same donor	The 2020 Tsitologiya article and the 2021 Cell and Tissue Biology article represent the Russian original and English translation of the same report. V0146 is excluded as a translated duplicate; V0119 is retained.	Translation duplicate excluded before synthesis; the original report was retained.
Study family 2	Human Umbilical Cord-Derived Mesenchymal Stem Cells Do Not Undergo Malignant Transformation during Long-Term Culturing in Serum-Free Medium<br>Monitoring the biology stability of human umbilical cord-derived mesenchymal stem cells during long-term culture in serum-free medium	Full-text comparison showed an identical seven-author team, the same serum-free long-term hUC-MSC culture program, and overlapping passage-series stability endpoints, consistent with a shared experimental series.	Counted as one study family in the study-family sensitivity check.
Potential overlap group 1	Comparative Bioactivity Analysis for Off-the-Shelf and Culture-Rescued Umbilical Cord-Derived Mesenchymal Stem/Stromal Cells in a Xeno- and Serum-Free Culture System	Full-text comparison identified related author/platform context but did not establish reuse of the same experimental units.	Retained as separate study units because full-text comparison did not establish shared experimental units.

	Standardized xeno- and serum-free culture platform enables large-scale expansion of high-quality mesenchymal stem/stromal cells from perinatal and adult tissue sources		
Potential overlap group 2	Large Scale Manufacturing of Wharton's Jelly Mesenchymal Stromal Cells for Clinical Use<br>Microcarrier-based clinical-grade manufacturing of therapeutic Wharton's jelly mesenchymal stromal cells	The reports originated from the same Soder/Dudley manufacturing group and concerned sequential development of a WJ-MSC clinical-grade platform. Full-text comparison did not establish reuse of the same donor/batch experimental units.	Retained as separate study units because full-text comparison did not establish shared experimental units.
Potential overlap group 3	Optimization of Culture Media for Human Umbilical Cord-Derived Mesenchymal Stem Cell Production<br>A GMP-compliant manufacturing method for Wharton's jelly-derived mesenchymal stromal cells	The reports came from the same Chu/Liang group and WJ/UC-MSC platform; the 2024 report described GMP manufacturing and the 2025 report focused on culture-media optimization. Shared platform context was evident, but reuse of the same experimental units was not established.	Retained as separate study units because full-text comparison did not establish shared experimental units.
Potential overlap group 4	Comparison of different methods for the isolation of mesenchymal stem cells from human umbilical cord Wharton's jelly<br>Effect of culture media on expansion properties of human umbilical cord matrix-derived mesenchymal cells	The reports came from the same research group and year and evaluated isolation and media/expansion questions in related hUC-MSC preparations. Full-text comparison did not establish reuse of the same experimental units.	Retained as separate study units because full-text comparison did not establish shared experimental units.
Potential overlap group 5	Immunogenicity of human umbilical cord mesenchymal stem cells during ex vivo expansion<br>Characteristics of human umbilical cord mesenchymal stem cells during ex vivo expansion	The reports involved an overlapping Ji/Guan author group and consecutive studies of ex vivo expansion characteristics and immunogenicity. A shared cell series was plausible but not demonstrated by full-text comparison.	Retained as separate study units because full-text comparison did not establish shared experimental units.

Table S5B. Manufacturing-domain frequencies at report and study-family levels.

Manufacturing domain	Reports, n (%) N = 238	Study families, n (%) N = 236
Expansion/passaging/senescence	94 (39.5%)	93 (39.4%)
Media/supplements	69 (29.0%)	68 (28.8%)
Isolation/tissue processing	67 (28.2%)	67 (28.4%)
GMP/quality/release	48 (20.2%)	46 (19.5%)
Starting material/source	36 (15.1%)	36 (15.3%)
Scale-up/3D/bioreactor	36 (15.1%)	36 (15.3%)
Cryopreservation/storage	35 (14.7%)	35 (14.8%)
Potency/functional CQA	31 (13.0%)	31 (13.1%)
Oxygen/process environment	22 (9.2%)	22 (9.3%)

Table S5C. Direct-comparison counts at report and study-family levels.

Manufacturing contrast	Reports, n	Study families, n
2D vs 3D/bioreactor	23	23
Explant vs enzymatic isolation	22	22
Fresh/native vs cryopreserved	19	19
HPL vs FBS	12	12
Hypoxia vs normoxia	10	10

Table S6. Direct-comparison mapping and heterogeneity assessment.

Contrast	Reports	Main outcome domains	Direction/consistency pattern	Key modifiers	Pooling decision/ design-robustness interpretation
HPL vs FBS	12	Proliferation, PDT, yield, CFU-F, phenotype/function	Human-derived supplementation often supported robust expansion, but superiority over FBS was not consistent across formulations and process stages.	HPL source/pooling, basal medium, concentration, heparin, seeding density, passage/timepoint	Descriptive mapping; no coherent common effect estimate. Within-process and same-donor/matched comparisons were weighted most strongly; unmatched formulation and donor comparisons were interpreted cautiously.
Explant vs enzymatic isolation	22	Primary yield, culture success, outgrowth time, phenotype	Mixed; neither approach was consistently superior across yield, outgrowth time, culture success, and phenotype.	Tissue compartment, explant geometry, enzyme identity/concentration, hybrid workflows, assessment timepoint	Descriptive mapping; no coherent common effect estimate. Same-tissue or split-sample comparisons were given greater interpretive weight than unrelated donor or protocol comparisons.

Fresh/native vs cryopreserved	19	Yield, viability, growth, phenotype, function	Identity/viability were often preserved, whereas growth, function, and recovery depended on cryopreservation stage and recovery interval.	Starting-tissue vs cell-bank vs final-product freezing, cryoprotectant, storage duration, recovery period	Descriptive mapping; interventions and endpoints were not commensurable. Same-donor or same-batch pre/post or split-manufacturing comparisons were weighted more strongly than unrelated cryopreservation cohorts.
2D vs 3D/bioreactor	23	Yield, fold expansion, PDT, harvest recovery, CQA comparability	Feasibility was demonstrated across evaluated platforms, but productivity and CQA differences were platform-specific and not directionally uniform.	Platform architecture, adherent/suspension format, microcarrier, mass transfer, harvest time, matched planar control	Descriptive mapping; platform definitions and endpoints were heterogeneous. Matched planar controls and same-process platform comparisons were weighted more strongly than scale-up reports without a directly matched 2D control.
Hypoxia vs normoxia	10	Proliferation, phenotype, senescence, immunomodulatory function	Mixed and context-dependent across proliferation, senescence, phenotype, and immunomodulatory outcomes.	Oxygen level, duration, culture format, co-interventions, endpoint/timepoint	Descriptive mapping; no coherent common effect estimate. Matched oxygen-condition comparisons within the same donor/process were weighted more strongly than studies differing simultaneously in culture format or co-interventions.

Table S7. Source-mapped quantitative audit for statements cited in the main process synthesis.

**Domain**	**Study (year)**	**Comparison/ context**	**Biological n**	**Endpoint**	**Intervention /result**	**Comparator/result**	**Unit/ timepoint**
Starting material	Subramanian et al. (2015)	Wharton's jelly vs other umbilical-cord compartments	5 cords	Fresh-cell yield	Wharton's jelly: 4.61 ± 0.57×10^6	Other compartments: lower	cells/cm tissue
Isolation	Smith et al. (2016)	Simplified closed enzymatic workflow vs preceding workflow	24 overall; ≥3 cords per variable	Cells per centimeter	>10-fold increase	Preceding workflow: reference	relative yield
Isolation	De Bruyn et al. (2011)	Enzyme-free whole-segment adhesion	50 cords	Primary isolation success	50/50 cords yielded MSCs	Not applicable	successful cords
Isolation	Lee et al. (2022)	2–4 mm minimal-cube explant vs enzymatic digestion	5 UC donors overall*	Primary yield	Explant: 3.92 ± 0.83×10^6	Enzymatic digestion: 10.2-fold lower	cells/g tissue
Isolation	Lee et al. (2022)	2–4 mm minimal-cube explant vs enzymatic digestion	5 UC donors overall*	CD146 expression	Explant: 73.4	Enzymatic digestion: 25.7	%
Isolation	Wang et al. (2014)	Explant vs enzyme digestion vs enzyme-assisted explant	10 cords overall*	Culture success	37 / 37 / 75	Three workflows	%; primary culture
Isolation	Wang et al. (2014)	Explant vs enzyme digestion vs enzyme-assisted explant	10 cords overall*	Primary outgrowth time	16.00 ± 3.65 / 5.50 ± 2.08 / 4.71 ± 1.97	Three workflows	days
Process standardization	Todtenhaupt et al. (2023)	Standardized isolation/expansion workflow	90 donors	Processing interval	Up to 48	Not applicable	hours after collection
Process standardization	Todtenhaupt et al. (2023)	Standardized isolation/expansion workflow	90 donors	Purity/ viability	Purity ≈98.9; viability >97	Not applicable	%
Media/xeno-free	Swamynathan et al. (2014)	Serum-/xeno-free system vs FBS	3 cords	Population doubling time	32.13 ± 3.95	45.24 ± 4.35	hours

Domain	Study (year)	Comparison/ context	Biological n	Endpoint	Intervention /result	Comparator/result	Unit/ timepoint
Media/xeno-free	Swamynathan et al. (2014)	Serum-/xeno-free system vs FBS	3 cords	Cumulative population doublings	18.32 ± 0.38	16.25 ± 0.26	CPD; end of P5
Scale-up	Neumann et al. (2014)	Disposable rotating-bed bioreactor	NR	Final cell output	24.6 ± 2.4×10^6 from 3×10^6 inoculum	Static comparator used for PDT	cells; day 5
Scale-up	Neumann et al. (2014)	Disposable rotating-bed bioreactor	NR	Fold expansion	8.2 ± 0.8	Not stated for static comparator in main text	fold; day 5
Scale-up	Neumann et al. (2014)	Rotating-bed vs static culture	NR	Population doubling time	39.6 ± 1.8	31	hours
2D vs 3D	López-Fernández et al. (2024)	3 L single-use stirred tank vs planar 2D	NR in full text	Final cell yield	3D: ≈9.48× 10^8	2D: ≈9.73×10^8	cells; 3D day 6 vs 2D day 8
2D vs 3D	López-Fernández et al. (2024)	3 L single-use stirred tank vs planar 2D	NR in full text	Viability	3D: >90	2D: >90 / comparable CQAs	%
Scale-up	Soder et al. (2025)	2 L to 50 L single-use scale-up	Assay n = 3; biological n unclear*	Final cell output	≈37×10^9	2D CQA/potency†	cells; 7-day 50 L stage†
Scale-up	Soder et al. (2025)	2 L to 50 L single-use scale-up	Assay n = 3; biological n unclear*	Expansio/ harvest efficiency	27-fold; 95%	2D CQA/potency†	7-day 50 L stage†
Cryopreservation	Zhang et al. (2022)	Before vs 12-month cryopreservation	3 UC donors	Cell count	Before: 1.00×10^7	12 months: 0.943×10^7	cells
Cryopreservation	Zhang et al. (2022)	Before vs 12 months cryopreservation	3 UC donors	Viability	Before: 96.34	12 months: 93.81	%

Biological n refers to independent cord donors or biological units, rather than the inoculum cell count, counting aliquots, wells, or technical assay repeats. * Overall study donor counts or reported assay repeats do not establish independent biological n for each endpoint; the endpoint-level evaluable donor/batch denominator is unclear in

these entries. † Soder et al. included 2D quality/function benchmarks, but a matched planar total-output/time comparison was not established. The seven-day 50 L stage was part of a 13-day seed-train workflow. Detailed donor, matching, and replication qualifications are given in Table S8.

Table S8. Source-level experimental-design features of key reports supporting the detailed process synthesis.

Study/process question	Within-study comparator/ design	Same-donor or matched status	Biological units/replication descriptors	Interpretation and remaining limits
Subramanian et al. (2015) [2] Starting compartment	Comparison of WJ, subamnion, perivascular, amniotic membrane, and mixed-cord preparations.	Compartments obtained from the same cords.	5 independent cords; within-cord compartment comparison.	Reduces donor confounding for the compartment contrast; small donor set and compartment-specific processing limit generalization.
Smith et al. (2016) [10] Isolation/medium	Procedural optimization with alternative processing conditions assigned to cord samples; medium comparisons also reported.	Within-cord procedural comparisons; not all 24 cords support each contrast.	24 cords overall; each procedural variable assessed in ≥3 different cords. Technical duplicates were averaged.	Replicated within-cord comparisons support the tested process changes; interpret each contrast using its own biological denominator.
Emnett et al. (2016) [12] Isolation	Tissue homogenization versus collagenase isolation; subsequent media comparisons.	Cord divided into segments for comparison of isolation approaches.	10 cords in the UC series; subsets used for different endpoints. Triplicate CFU-F wells are technical replicates.	Same-source material reduces donor confounding for isolation; multiple processing/media differences and endpoint-specific subsets constrain attribution.
De Bruyn et al. (2011) [13] Enzyme-free isolation	Non-comparative whole-segment adhesion workflow; no within-study alternative isolation method.	Not applicable to the isolation-success observation.	50 cords; MSC isolation successful in 50/50. Later assays need not share this denominator.	Supports feasibility and reproducible isolation in the tested series; does not establish superiority over enzymatic isolation.
Lee et al. (2022) [14] Explant geometry/ isolation	Minimal-cube explant conditions compared with other cube sizes and enzymatic digestion.	Donor pairing is not explicit for every reported endpoint.	5 UC donors overall; reported representative experiments performed in triplicate do not establish 3 independent donors per endpoint.	Provides direct process contrasts; biological n and pairing for each plotted endpoint remain important limits on confidence.
Wang et al. (2014) [15] Primary culture methods	Tissue explant, enzymatic digestion, and enzyme-assisted explant workflows compared.	Allocation and same-donor pairing not explicit in the charted quantitative results.	10 fresh cords stated for the study; evaluable denominator per method/endpoint unclear.	Comparison suggests workflow-dependent success and outgrowth; do not infer matched pairs or an endpoint n from percentages.
Todtenhaupt et al. (2023) [20] Workflow standardization	Multi-step optimization of collection, processing, isolation, and expansion;	Matching varies by experimental stage; the overall cohort is not a set	90 UC donors overall; endpoint-level experimental subsets should be distinguished from the full cohort.	Supports robustness within the defined workflow across donors; does not establish universal superiority of every selected condition.

	internal condition comparisons.	of 90 matched pairs for every contrast.		
Swamynathan et al. (2014) [25]<br>Serum-/xeno-free expansion	Defined serum-/xeno-free systems versus FBS-containing medium; large-scale comparison uses the same cells.	Same-cell comparison for the large-scale expansion contrast.	10 cords overall: 3 in initial media comparisons, 7 in additional isolation validation; 3 cords in the large-scale media comparison.	Biological replication supports the formulation-specific growth contrast; medium and attachment substrate differ, so it is not an isolated HPL-versus-FBS effect.
Nguyen et al. (2021) [26]<br>Thaw/recovery culture	Freshly thawed versus culture-rescued UC-MSCs; paired analyses.	Same starting preparations split between immediate testing and an additional recovery passage.	5 UC-MSC donor preparations for most assays; T-cell suppression uses 3 MSC preparations and 6 PBMC donors.	Paired design strengthens inference about the thaw/recovery workflow; different assay denominators and the extra passage limit attribution to thaw alone.
Neumann et al. (2014) [34]<br>Rotating-bed expansion	Dynamic rotating-bed expansion with a static-culture PDT comparator.	Donor matching not established in the quantitative audit.	Independent donor/batch n not specified in the source-mapped quantitative audit (Table S7).	Supports dynamic expansion feasibility. Reported PDT does not support faster growth than static culture; unclear biological replication limits comparative confidence.
López-Fernández et al. (2024) [31]<br>3 L STR versus planar culture	Direct platform comparability exercise assessing yield and predefined CQAs.	Same-donor pairing not established in the source-mapped audit.	Independent donor/batch n not specified in Table S7; inoculum and final cell counts are not biological replicate counts.	Supports platform-specific CQA comparability. Yield measured at different harvest times (3D day 6, 2D day 8) is not a standardized equal-time effect.
Soder et al. (2025) [32]<br>2 L to 50 L scale-up	Scale-up development with 2D quality/function benchmarks; total-output/time contrast is not a paired planar comparison.	Paired donor-level platform matching not established for the quoted 50 L output.	Several figures report n = 3; independent donor/batch denominator for the quoted output is unclear. Independent-donor 2 L runs are described.	Supports large-scale feasibility and product characterization; 37 × 10^9 cells refers to the 7-day 50 L stage within a 13-day seed train, rather than a matched planar productivity advantage.
Skiles et al. (2020) [37]<br>Split manufacturing/ cryopreservation	Fresh-tissue digestion pathway versus cryopreserved-tissue explant pathway.	Same-donor tissue split between manufacturing pathways.	10 input UC samples; evaluable n may differ across endpoints.	Reduces donor confounding but changes preservation and isolation together; differences cannot be attributed to freezing alone.
Zhang et al. (2022) [42]<br>Long-term cell storage	Fresh preparations versus cells stored for 12 months; pre/post count and viability assessment and later cell characterization.	Cells from the same donors split into fresh and cryopreserved preparations.	3 UC donors; assays performed in triplicate/repeated runs do not increase the donor n.	Supports within-process storage comparison. Phenotype, differentiation, and karyotype assessed after re-expansion to P4 cannot replace immediate post-thaw potency testing.

Note: CQAs, critical quality attributes; FBS, fetal bovine serum; HPL, human platelet lysate; PBMCs, peripheral blood mononuclear cells; PDT, population-doubling time; STR, stirred-tank reactor; UC, umbilical cord; WJ, Wharton's jelly.
